# Comparison of multiple obesity indices for cardiovascular disease risk classification in South Asian adults: The CARRS Study

**DOI:** 10.1371/journal.pone.0174251

**Published:** 2017-04-27

**Authors:** Shivani A. Patel, Mohan Deepa, Roopa Shivashankar, Mohammed K. Ali, Deksha Kapoor, Ruby Gupta, Dorothy Lall, Nikhil Tandon, Viswanathan Mohan, M. Masood Kadir, Zafar Fatmi, Dorairaj Prabhakaran, K. M. Venkat Narayan

**Affiliations:** 1 Rollins School of Public Health, Emory University, Atlanta, Georgia, United States of America; 2 Centre for Control of Chronic Conditions, Gurgaon, Haryana, India; 3 Madras Diabetes Research Foundation, Chennai, Tamil Nadu, India; 4 Public Health Foundation of India, Gurgaon, Haryana, India; 5 All India Institute of Medical Sciences, New Delhi, India; 6 Institute of Public Health Bengaluru, Bengaluru, Karnataka, India; 7 Aga Khan University, Karachi, Sindh, Pakistan; Medizinische Universitat Innsbruck, AUSTRIA

## Abstract

**Background:**

We comparatively assessed the performance of six simple obesity indices to identify adults with cardiovascular disease (CVD) risk factors in a diverse and contemporary South Asian population.

**Methods:**

8,892 participants aged 20–60 years in 2010–2011 were analyzed. Six obesity indices were examined: body mass index (BMI), waist circumference (WC), waist-height ratio (WHtR), waist-hip ratio (WHR), log of the sum of triceps and subscapular skin fold thickness (LTS), and percent body fat derived from bioelectric impedance analysis (BIA). We estimated models with obesity indices specified as deciles and as continuous linear variables to predict prevalent hypertension, diabetes, and high cholesterol and report associations (prevalence ratios, PRs), discrimination (area-under-the-curve, AUCs), and calibration (index χ^2^). We also examined a composite unhealthy cardiovascular profile score summarizing glucose, lipids, and blood pressure.

**Results:**

No single obesity index consistently performed statistically significantly better than the others across the outcome models. Based on point estimates, WHtR trended towards best performance in classifying diabetes (PR = 1.58 [1.45–1.72], AUC = 0.77, men; PR = 1.59 [1.47–1.71], AUC = 0.80, women) and hypertension (PR = 1.34 [1.26,1.42], AUC = 0.70, men; PR = 1.41 [1.33,1.50], AUC = 0.78, women). WC (mean difference = 0.24 SD [0.21–0.27]) and WHtR (mean difference = 0.24 SD [0.21,0.28]) had the strongest associations with the composite unhealthy cardiovascular profile score in women but not in men.

**Conclusions:**

WC and WHtR were the most useful indices for identifying South Asian adults with prevalent diabetes and hypertension. Collection of waist circumference data in South Asian health surveys will be informative for population-based CVD surveillance efforts.

## Introduction

Against the backdrop of the global nutrition and epidemiologic transitions [1,2], measuring and tracking overweight and obesity in adulthood is gaining importance in low- and middle-income countries. Epidemiologists and clinicians commonly employ anthropometric indices as convenient and informative body composition metrics to gauge population- and individual-level risk for cardiovascular disease (CVD) [3–7]. In addition, advancing technology has made alternative approaches for measuring percent body fat, such as bioelectric impedance analysis (BIA), feasible in large-scale field settings. In light of constraints of time, inconvenience, resources, and participant fatigue, identifying the best metric of obesity as it relates to particular health outcomes can allow investigators and clinicians to make informed decisions about which body composition measures to include.

The existing literature suggests that ethnicity is an important consideration when choosing anthropometric indicators of CVD risk. The relationship between anthropometric and BIA-based indices of body fat and true body fat vary by ethnicity, and additionally, the empirical correlation between anthropometrically-measured obesity and cardiovascular disease risk factors also differs by ethnicity. Specifically, Asians appear to have higher overall body fat per unit of body mass index (BMI) and higher truncal fat per kilogram of total body fat compared with Whites [8–11]. South Asians further stand apart from other ethnic groups by being more likely to have diabetes and cardiovascular conditions compared to non-South Asians at any given BMI [12–15]. There are relatively few recent data, however, simultaneously examining how multiple obesity indices relate to multiple domains of cardiovascular health and to overall cardiovascular risk in the apparently aberrant South Asian ethnic group.

We comparatively evaluated the performance of six indices of obesity—body mass index (BMI), waist circumference (WC), waist-height ratio (WHtR), waist-hip ratio (WHR), and log of the sum of triceps and subscapular skin fold thickness (LTS), and percent body fat derived from BIA (BIA)—as tools to identify adults with CVD risk factors in a diverse and contemporary population (2010–2012) of urban South Asian adults ages 20–60 years. The performance of each obesity indicator was assessed on the basis of predictive value, calibration, and discrimination in classifying individuals with prevalent diabetes, hypertension, and elevated total blood cholesterol. We also examined the relation between obesity indices and a composite unhealthy cardiovascular profile score.

## Methods

### Study population and data collection

The Center for Cardio-metabolic Risk Reduction in South Asia (CARRS) Surveillance Study is an ongoing multi-center, community-based cohort study designed to estimate the prevalence and incidence of cardio-metabolic risk factors and diseases in Chennai, India, New Delhi, India and Karachi, Pakistan [16]. These three diverse cities were selected to reflect the region’s rapid socioeconomic and epidemiologic transitions. In 2010–2011, 16,288 adults ages 20 years and older were enrolled in CARRS (response rates: 94.7% for questionnaire and 84.3% for bio-specimens). The CARRS study was approved by the Institutional Ethics Committee of the Public Health Foundation of India (TRC-IEC-34/09 (IRB no. IRB00006658) and the Institutional Review Board of Emory University (IRB00044159). Participants provided written consent to participate in the study. Data collection was conducted by trained field researchers. Bio-specimens were analyzed by accredited laboratories that were RIQAS (Randox International Quality Assessment Scheme) certified as an external quality assurance mechanism. Detailed information on sampling and data collection has been previously published [16]. See S1 File for the baseline questionnaire.

For the present analysis, participants ages > 60 years were excluded (n = 1,666) due to the potential loss of muscle mass related to age in older adults [17], 28% of participants were excluded due to missing anthropometric measures, 2% were excluded due to missing body fat percent, and 6% were excluded because of missing laboratory data needed for cardiovascular risk factor definitions. The primary analysis reported here used baseline data (2010–2011) from 8,892 participants with complete anthropometric and CVD risk factor data.

### Indices of obesity

Height, weight, and waist data were measured by trained field staff at the home of participants. Five anthropometric measures were examined. Body mass index (BMI; weight in kilograms divided by the square of height in meters) was used to measure general obesity. Central obesity was measured using waist-based measures: waist circumference (centimeters; WC), waist-height ratio (waist in meters divided by height in meters; WHtR), and waist-hip ratio (waist in meters divided by hip circumference in meters; WHR). The log of the sum of triceps and subscapular skinfold thickness (in centimeters; LTS) was examined as an alternative measure of body fat that is not often reported in large surveys [18,19]. Finally, BIA-derived percent body fat [20] was measured by portable Tanita body composition analyzers (Tanita BC-418 in Chennai and New Delhi and Tanita BC-545 in Karachi). BIA uses electrical impedance measured through specific body parts (right arm, left arm, right leg, left leg, trunk), height, weight, and “athletic build” (defined as individuals engaged in intense physical exercise ≥10 hours per week currently or in the past) to estimate fat free mass, which is then used to compute percentage body fat. To measure electrical impedance, participants stand on the instrument’s weighing platform with bare feet to make contact with electrodes while their bare hands are gripped around the instrument’s handles. The Tanita instruments used DXA as the reference method and Asian-specific equations to compute percentage body fat. While the Tanita Asian-specific equations are proprietary, independent validation studies have indicated that this method will underestimate fat mass and percent body fat (correlations ranging from .60 [men] to .64 [women] between BIA methods and the DXA the gold standard [21]). We add to this literature by providing correlations of BIA with other anthropometric measures and associations of BIA with cardiovascular risk factors in South Asians.

### Cardiovascular profiles

Three CVD risk factors were examined: elevated cholesterol, hypertension, and diabetes. Elevated cholesterol was defined as total blood cholesterol ≥ 200 mg/dL or taking cholesterol lowering medication. Hypertension was based on the average of up to three blood pressure readings and defined as systolic blood pressure ≥ 140 or diastolic blood pressure ≥ 90 mmHg or taking blood pressure lowering medication. Diabetes was defined as fasting blood glucose ≥ 126 mg/dl or HbA1c ≥ 6.5% or taking glucose lowering medication.

Unlike binary risk factors, continuously measured levels of metabolic biomeasures are not subject to differences in choice of cut-points and provide additional granularity of information beyond dichotomized disease states. We therefore also examined as an outcome a composite of seven metabolic biomeasures in their continuous form: total blood cholesterol (mg/dL), triglycerides (mg/dL), low-density lipoprotein cholesterol (mg/dL), systolic blood pressure (mmHg), diastolic blood pressure (mmHg), fasting blood glucose (mg/dL), and glycosylated hemoglobin (HbA1c, %). Because these biomeasures tend to be correlated with one another, we used principal components analysis to summarize them into a single composite unhealthy cardiovascular profile score. Principal components analysis is a technique to reduce multiple correlated variables to a smaller set of uncorrelated “components.” These components are the sum of the initial variables weighted to account for the empirical variability of each indicator. The first principal component by definition is a continuous variable that contains the largest amount of variance across the indicators and is per convention treated as the summary “index” used in further analysis [22]. In our analysis, the first principal component explained 39% of the variance among the seven indicators in the analytic sample. This level of variance explained indicated its suitability as a coherent summary risk score, and we describe this as the unhealthy cardiovascular profile score. The score was standardized to have mean = 0 and SD = 1, with a higher score indicating worse cardiovascular health. Correlations of individual cardiovascular indicators with the final cardiovascular risk factor score ranged from .56 to .70. Mean values of the seven indicators used to build the score by quartile of the score are shown in S1 Table. We note that mean levels of biomarkers were higher in progressively higher quartiles. For example, the difference in mean levels between the worst (highest) and best (lowest) quartiles were ~20 mmHg for diastolic blood pressure and ~40 mg/dL for fasting plasma glucose.

### Covariates

Sex, age in years, and city of residence were incorporated into the analyses as stratification (sex) or predictor (age, age-squared, and city of residence) variables in the models.

### Statistical analysis

Means, standard errors, and correlations of anthropometric indices and BIA-derived body fat were computed. In subsequent analyses, obesity measures were treated as exposures and individual CVD risk factors and the unhealthy cardiovascular profile score were treated as outcomes.

The first set of models estimated associations between deciles of obesity indices and CVD risk factors in order to facilitate comparisons across exposure measures, allow for potential non-linear relationships, and identify potential thresholds of CVD risk. These models directly estimated the prevalence ratios of binary cardiovascular risk factors at each decile relative to the lowest decile using Poisson regression with robust variance estimation [23]. Linear regression was used to estimate mean differences in the unhealthy cardiovascular profile score at each decile of obesity relative to the lowest decile. Deciles were included in outcome models as a 10-level categorical variable with the lowest decile as the referent.

A second set of models estimated associations of obesity indices specified as continuous exposures with outcomes. These models summarized the linear association between obesity indices and outcomes and included a squared term for each obesity index as this improved the fit of all estimated models. Model accuracy was assessed on the basis of “predictive value” (prevalence ratio associated with the obesity index and its square term), calibration (χ^2^ for the obesity index and its squared term), and discrimination (areas under the receiver operator characteristic curves, AUC) in each outcome model. The prevalence ratios, associated χ^2^ values, and predicted probabilities for the AUC calculation were obtained from Poisson regression models with robust variance estimation [23]. In models assessing accuracy, obesity indices were standardized to have M = 0, SD = 1 in order to compare prevalence ratio estimates across exposure measures.

All analyses were stratified by sex and were age-standardized to the South Asian population [24]. Models were also adjusted for continuous age in years, age-squared, and city of residence. Data were analyzed using SAS 9.4 statistical software (SAS Institute, Cary, NC).

Because 28% of the original sample was excluded due to missing anthropometry data, we conducted a sensitivity analysis by creating ten completed datasets using multiple imputation [25]. STATA statistical software was used to perform multiple imputation, and logistic regression models with continuous anthropometric measures were estimated using SAS software. Results largely remained the same and are shown in S5 Table.

## Results

A total of 3772 men and 5120 women ages 20–60 years were included in the primary analysis. Table 1 shows the age and residence of participants alongside the distribution (mean and standard error) of anthropometry and obesity measures in the sample. On average, men compared with women had lower BMI, WHtR, LTS, and BIA, and higher WC and WHR.

**Table 1:** Participant characteristics.

	Men (n = 3772)	Women (n = 5120)
	Means ± SD	
Demographic characteristics		
Mean Age, y	39.62 ±11.15	38.57 ±10.58
City of residence		
Chennai, %	42%	47%
Delhi, %	36%	30%
Karachi, %	23%	23%
Anthropometric measures		
Height, m	1.66 ± 0.07	1.52 ± 0.06
Weight, kg	67.15 ±14.95	61.46 ±14.22
WC, cm	87.85 ±13.57	83.82 ±13.40
Subscapular skinfold, mm	20.66 ± 9.01	23.60 ± 7.92
Triceps skinfold, mm	13.85 ± 6.90	21.41 ± 7.41
Obesity Indices*		
BMI	24.29 ± 4.96	26.49 ± 5.80
WHtR	0.53 ± 0.08	0.55 ± 0.09
WHR	0.94 ± 0.08	0.85 ± 0.08
LTS	3.45 ± 0.47	3.76 ± 0.36
BIA	21.93 ± 8.06	35.34 ± 9.31

BMI, body mass index; WC, waist circumference; WHtR, waist-height ratio; WHR, waist-hip ratio; LTS, log of the sum of triceps and subscapular skinfolds; BIA, bioelectric impedance analysis derived percent body fat.

*WC is shown under anthropometric measures but is examined as an obesity index throughout the paper.

Table 2 shows the correlation among the six examined measures of obesity. Correlations among the obesity indices ranged from 0.44 to 0.96 for men and 0.26 to 0.97 for women, and all correlations were statistically significantly greater than zero (statistical tests not shown). WC, WHtR, and BMI were most highly correlated (*r*>0.80 among men and women). WHR, despite being a waist-based measure, displayed lower correlations with WC and WHtR (*r* ranging from .61 to .63 for women and .68 to .73 for men) than correlations observed between WC, WHtR, and BMI. In general, correlations with LTS were stronger among men than women. BIA, which includes height and weight in the input equation, was most strongly correlated with BMI, but also showed reasonably high correlations with WC and WHtR (*r* ranging from .72 to .75 in the sample).

**Table 2:** Unadjusted inter-correlations of obesity indices.

Sex	*Obesity index*	BMI	WC	WHtR	WHR	LTS	BIA
Men	BMI	1.00	.	.	.	.	.
	WC	0.85	1.00	.	.	.	.
	WHtR	0.85	0.96	1.00	.	.	.
	WHR	0.48	0.73	0.74	1.00	.	.
	LTS	0.73	0.70	0.68	0.44	1.00	.
	BIA	0.78	0.74	0.75	0.49	0.65	1.00
Women	BMI	1.00	.	.	.	.	.
	WC	0.81	1.00	.	.	.	.
	WHtR	0.82	0.97	1.00	.	.	.
	WHR	0.25	0.64	0.63	1.00	.	.
	LTS	0.66	0.63	0.61	0.26	1.00	.
	BIA	0.81	0.73	0.72	0.26	0.65	1.00

BMI, body mass index; WC, waist circumference; WHtR, waist-height ratio; WHR, waist-hip ratio; LTS, log of the sum of triceps and subscapular skinfolds; BIA, bioelectric impedance analysis derived percent body fat.

**Fig 1:**
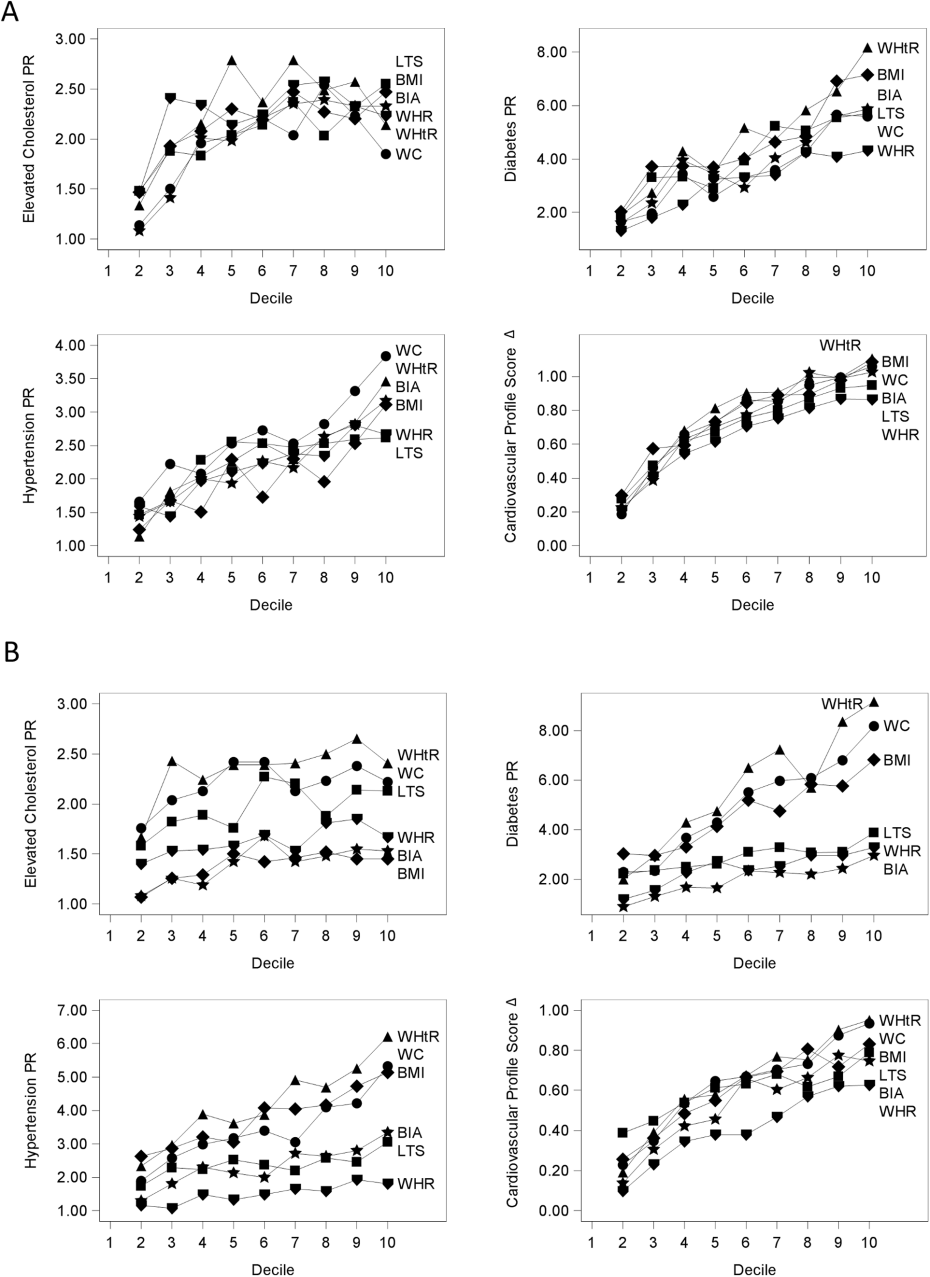
Associations (PRs or mean difference) between deciles of obesity indices and cardiovascular risk factors in urban South Asian men (panel A) and women (panel B). Models compare cardiovascular outcomes in higher compared to the lowest decile of each anthropometric index and were adjusted for age in years, age-squared, and city of residence. Deciles were included in the outcome models as a 10-level categorical variable with the first decile as the reference category. Prevalence ratios are shown for diabetes, elevated cholesterol, and hypertension and mean differences (betas) are shown for the cardiovascular risk index. The y-axis differs across the plots to allow for better visualization of the point estimates Data are available in table form in S2 Table and S3 Table. Abbreviations: BMI, body mass index; WC, waist circumference; WHtR, waist-height ratio; WHR, waist-hip ratio; LTS, log of the sum of triceps and subscapular skinfolds; BIA, bioelectric impedance analysis derived percent body fat.

Fig 1A and 1B display associations (PRs or mean difference) between deciles of obesity indices and cardiovascular risk factors in men and women, respectively; the first (lowest) decile is the reference category. Corresponding point estimates with 95% confidence intervals are reported in S2 Table and S3 Table for men and women, respectively. Across binary CVD risk factors, the PR point estimate was higher in each progressive decile of the specified obesity measure relative to the lowest decile. This positive gradient in PRs by decile of obesity measure followed a curvilinear pattern for elevated cholesterol and the unhealthy cardiovascular profile score in men, but a largely linear pattern in all other models. Among men and women, there were few statistically significant differences between PRs at any given decile across obesity indices. Among women, we found that WHtR demonstrated the strongest associations (point estimates) with all CVD risk factors and unhealthy cardiovascular profile score in nearly all deciles.

**Fig 2:**
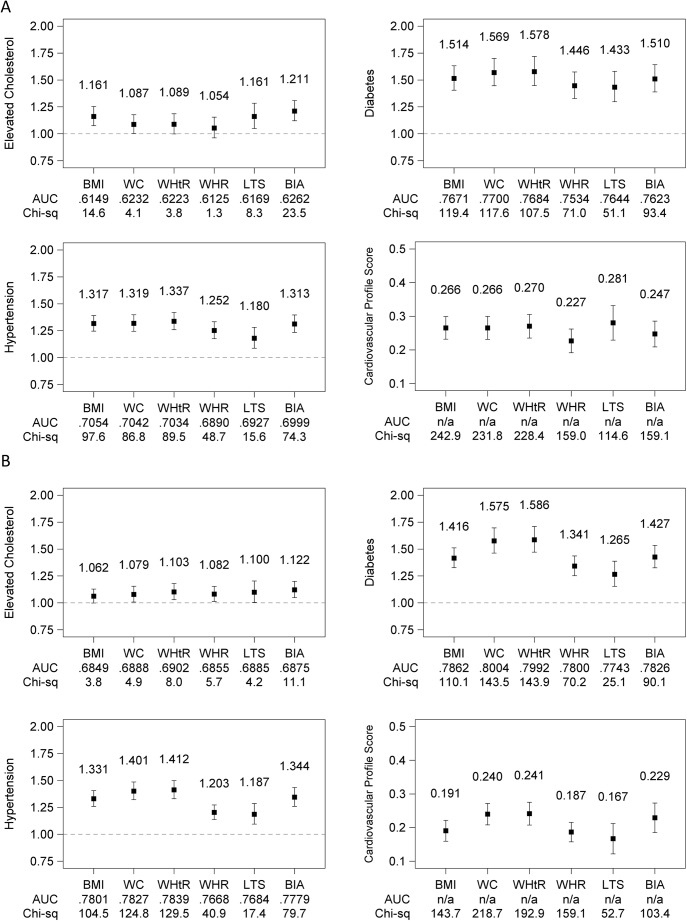
**Summary performance of obesity indices in classifying CVD risk factors in men (panel A) and women (panel B): Associations (prevalence ratios or mean difference), discrimination (area under the curve, AUC), and calibration (quasi-likelihood under the independence model criterion; lower value indicates better model fit).** All obesity indices were standardized to mean = 0 and SD = 1 to facilitate comparisons across measures. Associations were adjusted for age in years, the age-squared, and city of residence. Data are available in table form in S4 Table. Abbreviations: BMI, body mass index; WC, waist circumference; WHtR, waist-height ratio; WHR, waist-hip ratio; LTS, log of the sum of triceps and subscapular skinfolds; BIA, bioelectric impedance analysis derived percent body fat; AUC, area under the curve.

Fig 2A and 2B show associations (PRs or mean differences) between standardized, continuously measured obesity indices and prevalent CVD risk factors in men and women respectively adjusted for age and city of residence. Data are shown in table form in S4 Table. PR estimates represent the relative prevalence of each CVD risk factor associated with +1 SD difference in the obesity index. The mean difference in unhealthy cardiovascular profile score associated with +1 SD difference in the obesity index is also shown.

In models of elevated cholesterol, BIA had the largest PR point estimate of the 6 obesity indices in both men and women (Fig 2A and 2B), although the confidence intervals around the point estimate overlapped with those of the other obesity measures (S4 Table). The obesity indices were not distinguishable with respect to discriminating elevated cholesterol based on AUC. In men, BIA had the highest point estimates for all accuracy measures (PR = 1.211, AUC = 0.626, χ^2^ = 23.5). In women, BIA had the highest PR and χ^2^ (PR = 1.12, χ^2^ = 11.1) whereas the highest AUC was observed for WHtR (AUC = 0.690).

In models of diabetes, all obesity indices showed statistically significant associations (Fig 2A and 2B). Though the associations of the 6 obesity indices with diabetes were not statistically distinguishable among men, among women we observed WC and WHtR to have larger PRs, larger AUCs (indicating better discrimination), and larger index χ^2^ (indicating better model fit) than WHR and LTS. WHtR had the highest PR for diabetes (PR = 1.578 in men and PR = 1.586 in women), WC had the highest AUC (AUC = 0.770 in men and AUC = 0.800 in women), and BMI had the highest χ^2^ in men (χ^2^ = 119.4) and WHtR had the highest χ^2^ in women (χ^2^ = 143.9).

Models of hypertension followed a pattern similar to models of diabetes (Fig 2A and 2B). All obesity indices showed statistically significant associations with hypertension. WHtR had the highest PR for hypertension (PR = 1.337 in men and PR = 1.412 in women); BMI had the highest AUC in men (AUC = 0.7057) and WHtR had the highest AUC in women (AUC = 0.784); and BMI had the highest χ^2^ in men (χ^2^ = 97.6) and WHtR had the highest χ^2^ in women (χ^2^ = 129.5).

Models of the composite unhealthy cardiovascular profile score estimated the mean difference in the score (expressed as SDs) associated with a +1 SD of each obesity index; no AUC is computed for these models because the score was treated as a linear outcome. LTS was the most strongly correlated with the score in men (difference = 0.281 SD) while WHtR was most correlated with the score in women (difference = 0.241 SD), and variable χ^2^ in men was highest for BMI (χ^2^ = 242.9) and in women was highest for WC (χ^2^ = 192.9).

## Discussion

Based on a large contemporary cohort of regionally diverse urban South Asians, we found that commonly used indices of obesity were highly correlated. The associations of obesity indices with binary CVD risk factors and an unhealthy cardiovascular profile score were positive and largely linear across all levels of obesity indices. Our comprehensive analysis—considering strength of associations, discrimination, and calibration—found that no single obesity index consistently and statistically significantly performed better than the others. We note, however, that WC and WHtR tended toward the best performance in discriminating and classifying prevalent diabetes and hypertension based on point estimates of associations, AUCs, and model fit. Although BIA and LTS may be best-suited to identify elevated cholesterol among men, we did not find evidence that they were superior to the more commonly used metrics of BMI and WC. Finally, CVD risk factors were progressively more prevalent in each decile of anthropometry, with no threshold after which prevalence was markedly higher than the previous decile. This reiterates that anthropometric cutoffs to classify cardiovascular health are subject to arbitrary definitions of what level of cardiovascular risk is deemed adverse.

There has been much interest in whether measures of general obesity (e.g., BMI) or central obesity (e.g., WC-derived indicators) are superior at discriminating CVD risk factors [26]. BMI has long been recommended to assess adult overweight and obesity [27] and is the most commonly reported metric of obesity in the literature [7]. Indeed, the present study and a previous meta-analysis [26] demonstrate that BMI has strong potential to discriminate and classify CVD risk factors. WC and WHR have been advocated to complement BMI in chronic disease risk classification because it proxies abdominal adiposity [28–32]. With the caveat that the performance of obesity indices were statistically indistinguishable, among contemporary urban South Asian adults, WHtR tended to be most strongly related to CVD risk factors based on point estimates alone. Among men, +1 SD of WHtR (i.e., 0.08 unit of WHtR) was associated with a 9% higher probability of elevated cholesterol, 57% higher probability of diabetes, 32% higher probability of hypertension, and +0.27 SD of the unhealthy cardiovascular profile score; among women, the corresponding figures for a +1 SD (i.e., 0.09 unit of WHtR) were 8%, 59%, 41%, and +0.24 SD. WHtR was also the most strongly associated obesity indices with the composite unhealthy cardiovascular profile score in women.

Of all the outcomes, the strongest relationships were observed between obesity indices and diabetes, with +1 SD of an obesity index being associated with between 43% to 58% higher prevalence of diabetes. The strong, positive, and largely linear association between obesity indices and diabetes in South Asians has been described previously [33,34]. The weakest relationships were observed between obesity indices and elevated cholesterol, with +1 SD of an obesity index associated with between 6% to 21% higher prevalence of elevated cholesterol. The plots of PRs by deciles in Fig 1A showed that the association between obesity and elevated cholesterol may be curvilinear in men, and thus the summary linear models may not be appropriate when investigating this outcome.

The focus of this paper was classifying prevalent binary CVD risk factors and a composite measure of unhealthy cardiovascular profiles using simple indices of obesity. A strength of this analysis was our ability to simultaneously assess multiple anthropometric measures of obesity using recent, objectively collected data with uniform protocols from geographically diverse, population based samples of South Asians in urban Pakistan and North and South India. A limitation of the analysis includes the inability to report on future risk of disease associated with obesity indicators. Yet, even after excluding individuals with prior diagnoses of diabetes and hypertension in respective models, results were very similar (data not shown; results available from authors upon request). This strengthens the case for using anthropometric indicators to classify cardiovascular risk factors in individuals not yet diagnosed with these conditions. We are also unable to generalize to rural settings where the need for cost-effective approaches to CVD screening are even greater. A comparison of the performance of general and central obesity indicators in predicting future cardiovascular risk among both rural- and urban-dwelling South Asians will be a valuable addition to this literature as requisite data become available.

Clinicians and epidemiologists continue to debate which measure(s) of anthropometry should be used to define obesity and identify individuals at high risk for cardiovascular disease, particularly as they apply to South Asians. The lack of a robust and clear inflection point after which CVD factors escalate poses an ongoing challenge for defining cut-points to distinguish “obese” from “non-obese.” Our findings add further support to the utility of WC and WHtR to gauge the presence of CVD risk factors [12,28,35,36]. The results also suggest that reliance on BMI alone may hinder surveillance efforts due to its lower sensitivity in identifying CVD risk factors in the South Asian population. Yet, to our knowledge, WC data are not routinely collected in current national health surveys in the region [7]. In light of the mounting evidence that waist-based measures offer the best performance in classifying diabetes and hypertension—two leading causes of death and disability in South Asians—clinical assessments and population-based surveys of South Asians would do well to include WC in the battery of anthropometric measures.

## Supporting information

S1 Table Means of obesity indices and CVD risk factors by quartile of cardiovascular risk factor index. (DOCX)

S2 Table Associations of deciles of anthropometric indices with cardiovascular risk factors among men. Models compare cardiovascular outcomes in higher compared to the lowest decile of each anthropometric index and were adjusted for age in years, age-squared, city of residence. Deciles were included in the outcome models as a 10-level categorical variable with the first decile as the reference category. Prevalence ratios are shown for diabetes, elevated cholesterol, and hypertension and mean differences (betas) are shown for the cardiovascular risk index. Point estimates correspond to Fig 1A of the manuscript.
(DOCX)

S3 Table Associations of deciles of anthropometric indices with cardiovascular risk factors among women. Models compare cardiovascular outcomes in higher compared to the lowest decile of each anthropometric index and were adjusted for age in years, age-squared, and city of residence. Deciles were included in the outcome models as a 10-level categorical variable with the first decile as the reference category. Prevalence ratios are shown for diabetes, elevated cholesterol, and hypertension and mean differences (betas) are shown for the cardiovascular risk index. Point estimates correspond to Fig 1B of the manuscript.
(DOCX)

S4 Table Associations of obesity indices with cardiovascular risk factors and model accuracy measures. All obesity indices were standardized to mean = 0 and SD = 1 to facilitate comparisons across measures. Associations were adjusted for age in years, age-squared, and city of residence. Betas (mean adjusted differences) are shown for the cardiovascular risk index, and adjusted prevalence ratios are shown for diabetes, elevated cholesterol, and hypertension. Table data correspond to Fig 2 of the manuscript.
(DOCX)

S5 Table Associations of standardized BMI and WHtR with diabetes, cholesterol, and hypertension using multiply imputed data: A sensitivity analysis. The table shows results from models using multiply imputed data. Ten completed datasets were created using STATA to impute values for missing variables. Results are averaged across the analysis based on 10 completed datasets. Obesity indices were standardized to mean = 0 and SD = 1 to facilitate comparisons across measures, and all models adjusted for age in years, age-squared, and city of residence.
(DOCX)

S1 File Baseline questionnaires. (PDF)
