# Supplementary material for: Comparison of multiple obesity indices for cardiovascular disease risk classification in South Asian adults: The CARRS Study
Source: PLoS One. 2017 Apr 27;12(4):e0174251. doi: 10.1371/journal.pone.0174251 (PMC5407781; doi:10.1371/journal.pone.0174251)
Supplement: S1 Table — (DOCX) [file pone.0174251.s001.docx]

**Table S1**. Means of obesity indices and CVD risk factors by quartile of cardiovascular risk factor index

|  | Cardiometabolic Risk Index Quartile, Men | | | | Cardiometabolic Risk Index Quartile, Women | | | |
| --- | --- | --- | --- | --- | --- | --- | --- | --- |
| Obesity indices and CVD risk factors | Best quartile | 2^nd^ Quartile | 3^rd^ Quartile | Worst quartile | Best quartile | 2^nd^ Quartile | 3^rd^ Quartile | Worst quartile |
| Unhealthy cardiovascular profile score | -1.22 | -0.51 | 0.10 | 1.30 | -1.02 | -0.26 | 0.33 | 1.39 |
| Diastolic blood pressure (mmHg) | 70.40 | 76.68 | 82.13 | 90.38 | 73.57 | 80.82 | 85.33 | 92.14 |
| Glycosylated hemoglobin (HbA1c, %) | 5.38 | 5.68 | 5.99 | 7.43 | 5.41 | 5.68 | 6.07 | 7.39 |
| Fasting blood glucose (mg/dL) | 90.32 | 95.49 | 100.6 | 139.1 | 90.09 | 94.95 | 102.0 | 139.7 |
| Low-density lipoprotein cholesterol (mg/dL) | 83.29 | 103.3 | 119.3 | 135.1 | 81.14 | 103.5 | 116.2 | 131.6 |
| Systolic blood pressure (mmHg) | 103.8 | 112.6 | 120.7 | 136.7 | 112.5 | 121.4 | 127.9 | 138.8 |
| Total blood cholesterol (mg/dL) | 142.8 | 169.0 | 189.7 | 213.9 | 140.3 | 169.1 | 188.5 | 211.9 |
| Triglycerides (mg/dL) | 80.41 | 110.3 | 134.5 | 178.2 | 98.08 | 130.2 | 164.1 | 203.0 |
| Diabetes | 2% | 9% | 20% | 57% | 4% | 9% | 23% | 52% |
| Elevated Cholesterol | .2% | 8% | 34% | 65% | .8% | 8% | 35% | 65% |
| Hypertension | 3% | 10% | 28% | 63% | 6% | 19% | 39% | 63% |
