# Supplementary material for: Comparison of multiple obesity indices for cardiovascular disease risk classification in South Asian adults: The CARRS Study
Source: PLoS One. 2017 Apr 27;12(4):e0174251. doi: 10.1371/journal.pone.0174251 (PMC5407781; doi:10.1371/journal.pone.0174251)
Supplement: S3 Table — Models compare cardiovascular outcomes in higher compared to the lowest decile of each anthropometric index and were adjusted for age in years, age-squared, and city of residence. Deciles were included in the outcome models as a 10-level categorical variable with the first decile as the reference category. Prevalence ratios are shown for diabetes, elevated cholesterol, and hypertension and mean differences (betas) are shown for the cardiovascular risk index. Point estimates correspond to Fig 1B of the manuscript. (DOCX) [file pone.0174251.s003.docx]

Table S3. Associations of deciles of anthropometric indices with cardiovascular risk factors among women

|  | | BMI | | WC | | WHtR | | WHR | | LTS | | BIA% | |
| --- | --- | --- | --- | --- | --- | --- | --- | --- | --- | --- | --- | --- | --- |
|  | Decile | Mean | PR (95%CI) | Mean | PR (95%CI) | Mean | PR (95%CI) | Mean | PR (95%CI) | Mean | PR (95%CI) | Mean | PR (95%CI) |
| Cardiovascular risk index | 2 | 20.70 | 0.26 (0.17,0.35) | 70.50 | 0.23 (0.14,0.32) | 0.46 | 0.19 (0.10,0.29) | 0.78 | 0.10 (-0.03,0.22) | 3.41 | 0.39 (0.23,0.54) | 26.48 | 0.14 (0.05,0.22) |
|  | 3 | 22.72 | 0.36 (0.26,0.47) | 75.60 | 0.35 (0.25,0.45) | 0.50 | 0.39 (0.27,0.51) | 0.80 | 0.23 (0.12,0.34) | 3.57 | 0.45 (0.35,0.55) | 30.62 | 0.30 (0.20,0.41) |
|  | 4 | 24.35 | 0.48 (0.36,0.61) | 79.51 | 0.53 (0.42,0.65) | 0.52 | 0.56 (0.46,0.66) | 0.82 | 0.34 (0.22,0.47) | 3.68 | 0.54 (0.40,0.68) | 33.43 | 0.42 (0.32,0.52) |
|  | 5 | 25.70 | 0.55 (0.48,0.63) | 82.81 | 0.65 (0.55,0.74) | 0.54 | 0.58 (0.47,0.69) | 0.84 | 0.38 (0.23,0.53) | 3.76 | 0.61 (0.51,0.72) | 35.64 | 0.45 (0.35,0.56) |
|  | 6 | 26.97 | 0.67 (0.59,0.75) | 85.80 | 0.67 (0.57,0.77) | 0.56 | 0.67 (0.57,0.77) | 0.86 | 0.38 (0.28,0.48) | 3.84 | 0.63 (0.51,0.76) | 37.55 | 0.66 (0.58,0.74) |
|  | 7 | 28.33 | 0.69 (0.60,0.79) | 88.78 | 0.70 (0.60,0.81) | 0.58 | 0.77 (0.67,0.87) | 0.88 | 0.47 (0.34,0.60) | 3.91 | 0.68 (0.58,0.78) | 39.44 | 0.60 (0.46,0.75) |
|  | 8 | 29.95 | 0.81 (0.69,0.92) | 92.38 | 0.73 (0.61,0.85) | 0.61 | 0.75 (0.66,0.85) | 0.90 | 0.57 (0.45,0.69) | 3.99 | 0.62 (0.50,0.73) | 41.56 | 0.66 (0.54,0.79) |
|  | 9 | 32.11 | 0.72 (0.63,0.81) | 97.29 | 0.88 (0.74,1.01) | 0.64 | 0.90 (0.80,1.01) | 0.93 | 0.62 (0.51,0.72) | 4.08 | 0.67 (0.57,0.77) | 44.19 | 0.78 (0.66,0.90) |
|  | 10 | 36.85 | 0.83 (0.70,0.96) | 106.8 | 0.93 (0.81,1.06) | 0.70 | 0.95 (0.81,1.09) | 0.99 | 0.63 (0.47,0.79) | 4.21 | 0.79 (0.68,0.90) | 49.41 | 0.75 (0.62,0.87) |
| Diabetes | 1 | 17.70 | ref | 62.14 | ref | 0.41 | ref | 0.73 | ref | 3.04 | ref | 18.46 | ref |
|  | 2 | 20.70 | 3.02 (1.71,5.33) | 70.50 | 2.30 (1.19,4.47) | 0.46 | 1.99 (0.95,4.19) | 0.78 | 1.18 (0.66,2.10) | 3.41 | 2.23 (1.40,3.55) | 26.48 | 0.88 (0.51,1.52) |
|  | 3 | 22.72 | 2.96 (1.63,5.37) | 75.60 | 2.33 (1.25,4.33) | 0.50 | 2.95 (1.51,5.77) | 0.80 | 1.56 (0.95,2.58) | 3.57 | 2.37 (1.57,3.57) | 30.62 | 1.31 (0.79,2.15) |
|  | 4 | 24.35 | 3.30 (1.97,5.52) | 79.51 | 3.66 (2.07,6.48) | 0.52 | 4.29 (2.11,8.72) | 0.82 | 2.27 (1.32,3.90) | 3.68 | 2.50 (1.66,3.77) | 33.43 | 1.67 (1.13,2.45) |
|  | 5 | 25.70 | 4.13 (2.46,6.92) | 82.81 | 4.30 (2.25,8.20) | 0.54 | 4.76 (2.45,9.25) | 0.84 | 2.73 (1.62,4.58) | 3.76 | 2.62 (1.80,3.80) | 35.64 | 1.65 (1.09,2.49) |
|  | 6 | 26.97 | 5.19 (3.09,8.71) | 85.80 | 5.50 (2.95,10.26) | 0.56 | 6.49 (3.27,12.89) | 0.86 | 2.35 (1.43,3.88) | 3.84 | 3.09 (2.11,4.51) | 37.55 | 2.34 (1.63,3.35) |
|  | 7 | 28.33 | 4.75 (2.88,7.84) | 88.78 | 5.96 (3.38,10.53) | 0.58 | 7.23 (3.86,13.53) | 0.88 | 2.53 (1.55,4.14) | 3.91 | 3.27 (2.29,4.67) | 39.44 | 2.26 (1.61,3.18) |
|  | 8 | 29.95 | 5.82 (3.54,9.56) | 92.38 | 6.09 (3.55,10.47) | 0.61 | 5.70 (3.08,10.52) | 0.90 | 2.95 (1.81,4.81) | 3.99 | 3.08 (2.19,4.32) | 41.56 | 2.19 (1.44,3.33) |
|  | 9 | 32.11 | 5.77 (3.39,9.82) | 97.29 | 6.80 (3.89,11.90) | 0.64 | 8.36 (4.36,16.02) | 0.93 | 2.97 (1.69,5.21) | 4.08 | 3.09 (2.03,4.71) | 44.19 | 2.44 (1.62,3.69) |
|  | 10 | 36.85 | 6.81 (4.05,11.44) | 106.8 | 8.18 (4.76,14.05) | 0.70 | 9.16 (4.99,16.83) | 0.99 | 3.27 (1.96,5.47) | 4.21 | 3.88 (2.68,5.61) | 49.41 | 2.95 (2.00,4.36) |
| Elevated cholesterol | 1 | 17.70 | ref | 62.14 | ref | 0.41 | ref | 0.73 | ref | 3.04 | ref | 18.46 | ref |
|  | 2 | 20.70 | 1.07 (0.80,1.43) | 70.50 | 1.76 (1.23,2.52) | 0.46 | 1.67 (1.21,2.31) | 0.78 | 1.40 (1.09,1.80) | 3.41 | 1.58 (1.09,2.29) | 26.48 | 1.08 (0.85,1.37) |
|  | 3 | 22.72 | 1.26 (1.00,1.59) | 75.60 | 2.04 (1.50,2.77) | 0.50 | 2.43 (1.64,3.59) | 0.80 | 1.53 (1.12,2.10) | 3.57 | 1.82 (1.40,2.37) | 30.62 | 1.26 (0.93,1.71) |
|  | 4 | 24.35 | 1.29 (0.96,1.74) | 79.51 | 2.13 (1.57,2.88) | 0.52 | 2.24 (1.50,3.34) | 0.82 | 1.54 (1.15,2.08) | 3.68 | 1.89 (1.35,2.67) | 33.43 | 1.19 (0.82,1.73) |
|  | 5 | 25.70 | 1.50 (1.04,2.18) | 82.81 | 2.42 (1.81,3.25) | 0.54 | 2.39 (1.75,3.27) | 0.84 | 1.58 (1.15,2.19) | 3.76 | 1.76 (1.33,2.33) | 35.64 | 1.42 (1.11,1.81) |
|  | 6 | 26.97 | 1.42 (1.08,1.89) | 85.80 | 2.42 (1.74,3.38) | 0.56 | 2.39 (1.75,3.27) | 0.86 | 1.69 (1.36,2.11) | 3.84 | 2.27 (1.59,3.25) | 37.55 | 1.68 (1.31,2.16) |
|  | 7 | 28.33 | 1.46 (1.06,2.02) | 88.78 | 2.13 (1.63,2.78) | 0.58 | 2.41 (1.71,3.39) | 0.88 | 1.53 (1.18,1.98) | 3.91 | 2.20 (1.60,3.01) | 39.44 | 1.42 (1.04,1.96) |
|  | 8 | 29.95 | 1.52 (1.15,2.02) | 92.38 | 2.23 (1.64,3.03) | 0.61 | 2.50 (1.80,3.46) | 0.90 | 1.81 (1.38,2.36) | 3.99 | 1.88 (1.38,2.58) | 41.56 | 1.48 (1.11,1.98) |
|  | 9 | 32.11 | 1.45 (1.08,1.94) | 97.29 | 2.38 (1.76,3.22) | 0.64 | 2.65 (1.95,3.61) | 0.93 | 1.85 (1.44,2.38) | 4.08 | 2.14 (1.58,2.91) | 44.19 | 1.55 (1.18,2.02) |
|  | 10 | 36.85 | 1.45 (1.10,1.92) | 106.8 | 2.22 (1.65,2.99) | 0.70 | 2.41 (1.68,3.44) | 0.99 | 1.67 (1.31,2.14) | 4.21 | 2.13 (1.51,3.00) | 49.41 | 1.53 (1.13,2.08) |
| Hypertension | 1 | 17.70 | ref | 62.14 | ref | 0.41 | ref | 0.73 | ref | 3.04 | ref | 18.46 | ref |
|  | 2 | 20.70 | 2.63 (1.78,3.88) | 70.50 | 1.90 (1.11,3.27) | 0.46 | 2.34 (1.40,3.90) | 0.78 | 1.16 (0.81,1.68) | 3.41 | 1.73 (1.22,2.47) | 26.48 | 1.29 (0.92,1.81) |
|  | 3 | 22.72 | 2.86 (1.81,4.53) | 75.60 | 2.57 (1.51,4.39) | 0.50 | 2.96 (1.84,4.75) | 0.80 | 1.08 (0.74,1.57) | 3.57 | 2.29 (1.62,3.23) | 30.62 | 1.80 (1.34,2.42) |
|  | 4 | 24.35 | 3.20 (2.10,4.88) | 79.51 | 2.99 (1.87,4.78) | 0.52 | 3.89 (2.35,6.43) | 0.82 | 1.49 (1.13,1.98) | 3.68 | 2.24 (1.65,3.04) | 33.43 | 2.30 (1.70,3.11) |
|  | 5 | 25.70 | 3.05 (2.02,4.61) | 82.81 | 3.17 (2.03,4.94) | 0.54 | 3.62 (2.19,5.98) | 0.84 | 1.32 (1.01,1.74) | 3.76 | 2.52 (1.75,3.64) | 35.64 | 2.13 (1.67,2.72) |
|  | 6 | 26.97 | 4.08 (2.61,6.37) | 85.80 | 3.40 (2.09,5.53) | 0.56 | 3.87 (2.50,6.00) | 0.86 | 1.49 (1.13,1.96) | 3.84 | 2.37 (1.70,3.31) | 37.55 | 2.00 (1.52,2.62) |
|  | 7 | 28.33 | 4.05 (2.54,6.44) | 88.78 | 3.06 (1.92,4.85) | 0.58 | 4.92 (3.01,8.06) | 0.88 | 1.65 (1.27,2.13) | 3.91 | 2.20 (1.58,3.08) | 39.44 | 2.71 (2.10,3.49) |
|  | 8 | 29.95 | 4.16 (2.73,6.34) | 92.38 | 4.10 (2.61,6.45) | 0.61 | 4.70 (3.05,7.25) | 0.90 | 1.58 (1.23,2.03) | 3.99 | 2.58 (1.89,3.50) | 41.56 | 2.62 (2.02,3.41) |
|  | 9 | 32.11 | 4.72 (3.06,7.29) | 97.29 | 4.21 (2.66,6.67) | 0.64 | 5.25 (3.36,8.22) | 0.93 | 1.92 (1.53,2.40) | 4.08 | 2.45 (1.73,3.46) | 44.19 | 2.80 (2.22,3.54) |
|  | 10 | 36.85 | 5.14 (3.31,7.98) | 106.8 | 5.33 (3.31,8.58) | 0.70 | 6.22 (3.96,9.74) | 0.99 | 1.81 (1.40,2.35) | 4.21 | 3.06 (2.26,4.15) | 49.41 | 3.34 (2.58,4.33) |

PR, prevalence ratio; BMI, body mass index; WC, waist circumference; WHtR, waist-height ratio; WHR, waist-hip ratio; LTS, log of the sum of triceps and subscapular skinfolds; BIA, bioelectric impedance analysis derived percent body fat.

Models compare cardiovascular outcomes in higher compared to the lowest decile of each anthropometric index and were adjusted for age in years, age-squared, city of residence. Deciles were included in the outcome models as a 10-level categorical variable with the first decile as the reference category. Prevalence ratios are shown for diabetes, elevated cholesterol, and hypertension and mean differences (betas) are shown for the cardiovascular risk index. Point estimates correspond to Fig 1b of the manuscript.
