# Supplementary material for: Comparison of multiple obesity indices for cardiovascular disease risk classification in South Asian adults: The CARRS Study
Source: PLoS One. 2017 Apr 27;12(4):e0174251. doi: 10.1371/journal.pone.0174251 (PMC5407781; doi:10.1371/journal.pone.0174251)
Supplement: S4 Table — All obesity indices were standardized to mean = 0 and SD = 1 to facilitate comparisons across measures. Associations were adjusted for age in years, age-squared, and city of residence. Betas (mean adjusted differences) are shown for the cardiovascular risk index, and adjusted prevalence ratios are shown for diabetes, elevated cholesterol, and hypertension. Table data correspond to Fig 2 of the manuscript. (DOCX) [file pone.0174251.s004.docx]

Table S4. Associations of obesity indices* with cardiovascular risk factors and model accuracy measures

| Sex | Outcome | Obesity Index | Prevalence Ratio (95% CI) | Chi-Square | Area |
| --- | --- | --- | --- | --- | --- |
| Men | Elevated Cholesterol | BMI | 1.161 (1.076,1.254) | 14.6 | 0.615 |
|  |  | WC | 1.087 (1.003,1.177) | 4.1 | 0.623 |
|  |  | WHtR | 1.089 (1.000,1.186) | 3.8 | 0.622 |
|  |  | WHR | 1.054 (0.962,1.155) | 1.3 | 0.612 |
|  |  | LTS | 1.161 (1.049,1.284) | 8.3 | 0.617 |
|  |  | BIA | 1.211 (1.121,1.309) | 23.5 | 0.626 |
|  | Diabetes | BMI | 1.514 (1.406,1.631) | 119.4 | 0.767 |
|  |  | WC | 1.569 (1.446,1.702) | 117.6 | 0.770 |
|  |  | WHtR | 1.578 (1.447,1.720) | 107.5 | 0.768 |
|  |  | WHR | 1.446 (1.327,1.575) | 71.0 | 0.753 |
|  |  | LTS | 1.433 (1.298,1.581) | 51.1 | 0.764 |
|  |  | BIA | 1.510 (1.389,1.642) | 93.4 | 0.762 |
|  | Hypertension | BMI | 1.317 (1.247,1.391) | 97.6 | 0.705 |
|  |  | WC | 1.319 (1.244,1.398) | 86.8 | 0.704 |
|  |  | WHtR | 1.337 (1.259,1.420) | 89.5 | 0.703 |
|  |  | WHR | 1.252 (1.175,1.333) | 48.7 | 0.689 |
|  |  | LTS | 1.180 (1.087,1.282) | 15.6 | 0.693 |
|  |  | BIA | 1.313 (1.234,1.396) | 74.3 | 0.700 |
|  | Unhealthy cardiovascular profile score | BMI | 0.266 (0.232,0.299) | 242.9 | . |
|  |  | WC | 0.266 (0.231,0.300) | 231.8 | . |
|  |  | WHtR | 0.270 (0.235,0.306) | 228.4 | . |
|  |  | WHR | 0.227 (0.192,0.262) | 159.0 | . |
|  |  | LTS | 0.281 (0.229,0.332) | 114.6 | . |
|  |  | BIA | 0.247 (0.209,0.286) | 159.1 | . |
| Women | Elevated Cholesterol | BMI | 1.062 (1.000,1.128) | 3.8 | 0.685 |
|  |  | WC | 1.079 (1.009,1.155) | 4.9 | 0.689 |
|  |  | WHtR | 1.103 (1.030,1.180) | 8.0 | 0.690 |
|  |  | WHR | 1.082 (1.014,1.154) | 5.7 | 0.686 |
|  |  | LTS | 1.100 (1.005,1.204) | 4.2 | 0.688 |
|  |  | BIA | 1.122 (1.048,1.201) | 11.1 | 0.688 |
|  | Diabetes | BMI | 1.416 (1.327,1.511) | 110.1 | 0.786 |
|  |  | WC | 1.575 (1.462,1.697) | 143.5 | 0.800 |
|  |  | WHtR | 1.586 (1.471,1.711) | 143.9 | 0.799 |
|  |  | WHR | 1.341 (1.252,1.437) | 70.2 | 0.780 |
|  |  | LTS | 1.265 (1.154,1.387) | 25.1 | 0.774 |
|  |  | BIA | 1.427 (1.326,1.535) | 90.1 | 0.783 |
|  | Hypertension | BMI | 1.331 (1.260,1.405) | 104.5 | 0.780 |
|  |  | WC | 1.401 (1.321,1.487) | 124.8 | 0.783 |
|  |  | WHtR | 1.412 (1.331,1.499) | 129.5 | 0.784 |
|  |  | WHR | 1.203 (1.137,1.273) | 40.9 | 0.767 |
|  |  | LTS | 1.187 (1.095,1.286) | 17.4 | 0.768 |
|  |  | BIA | 1.344 (1.260,1.434) | 79.7 | 0.778 |
|  | Unhealthy cardiovascular profile score | BMI | 0.191 (0.159,0.222) | 143.7 | . |
|  |  | WC | 0.240 (0.208,0.272) | 218.7 | . |
|  |  | WHtR | 0.241 (0.207,0.276) | 192.9 | . |
|  |  | WHR | 0.187 (0.158,0.216) | 159.1 | . |
|  |  | LTS | 0.167 (0.122,0.212) | 52.7 | . |
|  |  | BIA | 0.229 (0.185,0.273) | 103.4 | . |

BMI, body mass index; WC, waist circumference; WHtR, waist-height ratio; WHR, waist-hip ratio; LTS, log of the sum of triceps and subscapular skinfolds; BIA%, bioelectric impedance analysis derived percent body fat; AUC, area under the curve

*The estimates reflect the obesity index and the square of the obesity index

**Estimates are mean differences. Because this is a continuous measure, no AUC is computed.

All obesity indices were standardized to mean=0 and SD=1 to facilitate comparisons across measures. Associations were adjusted for age, the square of age, and city of residence. Betas (mean adjusted differences) are shown for the cardiovascular risk index, and adjusted prevalence ratios are shown for diabetes, elevated cholesterol, and hypertension. Table data correspond to Figure 2a and 2b of the manuscript.
