# Supplementary material for: Comparison of multiple obesity indices for cardiovascular disease risk classification in South Asian adults: The CARRS Study
Source: PLoS One. 2017 Apr 27;12(4):e0174251. doi: 10.1371/journal.pone.0174251 (PMC5407781; doi:10.1371/journal.pone.0174251)
Supplement: S5 Table — The table shows results from models using multiply imputed data. Ten completed datasets were created using STATA to impute values for missing variables. Results are averaged across the analysis based on 10 completed datasets. Obesity indices were standardized to mean = 0 and SD = 1 to facilitate comparisons across measures, and all models adjusted for age in years, age-squared, and city of residence. (DOCX) [file pone.0174251.s005.docx]

Table S5. Associations of standardized BMI and WHtR with diabetes, cholesterol, and hypertension using multiply imputed data: A sensitivity analysis

|  | Men | | | |  | Women | | | |
| --- | --- | --- | --- | --- | --- | --- | --- | --- | --- |
| **Model** | PR | LB | UB |  |  | PR | LB | UB |  |
| Elevated Cholesterol |  | | | | | | | | |
| BMI | 1.154 | 1.086 | 1.227 |  |  | 1.069 | 1.014 | 1.127 |  |
| WHtR | 1.139 | 1.062 | 1.223 |  |  | 1.106 | 1.038 | 1.178 |  |
|  |  |  |  |  |  |  |  |  |  |
| *Diabetes* |  |  |  |  |  |  |  |  |  |
| BMI | 1.442 | 1.361 | 1.527 |  |  | 1.374 | 1.296 | 1.456 |  |
| WHtR | 1.512 | 1.420 | 1.610 |  |  | 1.531 | 1.442 | 1.627 |  |
|  |  |  |  |  |  |  |  |  |  |
| *Hypertension* |  |  |  |  |  |  |  |  |  |
| BMI | 1.312 | 1.257 | 1.370 |  |  | 1.299 | 1.240 | 1.361 |  |
| WHtR | 1.352 | 1.291 | 1.415 |  |  | 1.394 | 1.328 | 1.464 |  |
|  |  |  |  |  |  |  |  |  |  |

The table shows results from models using multiply imputed data. Ten completed datasets were created using STATA to impute values for missing variables. Results are averaged across the analysis based on 10 completed datasets. Obesity indices were standardized to mean=0 and SD=1 to facilitate comparisons across measures, and all models adjusted for age in years, age-squared, and city of residence.
