# Supplementary material for: Comparison of multiple obesity indices for cardiovascular disease risk classification in South Asian adults: The CARRS Study
Source: PLoS One. 2017 Apr 27;12(4):e0174251. doi: 10.1371/journal.pone.0174251 (PMC5407781; doi:10.1371/journal.pone.0174251)
Supplement: S1 File — (PDF) [file pone.0174251.s006.pdf]

CARRS: Surveillance Study

Instruction to the interviewer: HAS THE PARTICIPANT SIGNED THE INFORMED CONSENT? DO NOT PROCEED UNTIL THE CONSENT FORM HAS BEEN SIGNED.

Household ID ☐☐☐☐☐	Participant ID ☐☐☐☐☐
CEB Code ☐☐☐	Interviewer ID ☐☐☐☐
Date of interview: ☐☐☐☐☐☐ DD/ MM/ YY	Start Time [Hr:min] ☐☐ : ☐☐ HR MIN

**SECTION – 1: DEMOGRAPHIC, SOCIO-ECONOMIC AND RESIDENTIAL DETAILS**	
1.Name of the Participant:<br>First name:<br>Middle Name:<br>Surname:	 ______________________________________<br>______________________________________<br>______________________________________
2.Father's/Spouse's name:<br>First name:<br>Middle Name:<br>Surname:	 ______________________________________<br>______________________________________<br>______________________________________
3.Address/Details:<br>Street:<br>District:<br>State:<br>Postal Code:	
5.Telephone Number<br>Residence<br>Office<br>Mobile	 ______________________________________<br>______________________________________<br>______________________________________
6.Email ID	(1)<br>(2)
7.Place of Birth<br>District:<br>State:	
8.Age (in completed years)	☐☐☐
9.Date of birth (if available)	☐☐☐☐☐☐☐☐<br>DD / MM / YYYY

10.Sex	Male 1<br>Female 2<br>Trans-gender 3	
11.What is your marital status?	Single 1<br>Married 2<br>Widow/Widower 3<br>Separated/Divorced 4<br>Others 5	Others, specify<br>________________
12.What is your mother tongue? (State of origin)	Assamese 1<br>Balochi 2<br>Bengali 3<br>Gujarati 4<br>Hindi 5<br>Kannada 7<br>Kashmiri 8<br>Maithili 9<br>Malayalam 10<br>Marathi 11<br>Punjabi 14<br>Sindhi 15<br>Telugu 16<br>Tamil 17<br>Urdu 18<br>Others 19	Others, specify<br>________________
13.What religion do you follow? (Optional)	Hindu 1<br>Muslim 2<br>Sikh 3<br>Christian 4<br>Jain 5<br>Buddhism 6<br>No religion 7<br>Others (specify) 8<br>No response 9	Others, specify<br>________________
14.Do you belong to a particular caste or tribe? (Optional)	Yes 1<br>No 2<br>Don't know 3<br>Don't want to answer 4<br>Not applicable 5	
14.a. If "Yes"<br>What is your caste or tribe? (Optional)	Schedule caste 1<br>Schedule tribe 2<br>Other backward caste 3<br>Most backward 4<br>Others 5<br>Don't want to answer 6	Others (specify)<br>________________
15.Number of years of formal education*<br>* The total number of years the participant spent in any educational institution (schools, colleges, religious schools, etc.)		**years**

16. Educational status (highest attained degree)<br><br>** A person who can both read and write with understanding in any language without any formal education or passed any minimum educational standard.*<br><br>*** A person, who can neither read nor write or can only read but cannot write in any language.*	Professional degree/post graduate 1<br>Graduate (B.A/B.Sc/B.Com/Diploma) 2<br>Secondary School / Intermediary (ITI course, class XII/X or Intermediate) 3<br>High school (class V to IX) 4<br>Primary School (upto Class IV) 5<br>*Literate, no formal education 6<br>**Illiterate 7<br>Others 8	Others, specify<br>__________________<br>__________________
17. Your employment status?	Employed 1<br>Student 2<br>Housewife 3<br>Retired 4<br>Un-employed 5	**"1" go to 17.a Otherwise go to Q18**
17.a. If ***"Employed",*** what is your current occupation?<br>***[Use nearest applicable employment codes given below]***		
18. Have you been involved in any other occupation during past ten years?	Yes 1<br>No 2	**"2" go to Q. 19**
*18.a***.** If ***'YES'***, name the occupation?<br>***[Use nearest applicable employment codes given below]***		

Coding list for employment (for Q.17.a and Q.18.a)- refer to annexure for definition of skilled, semi-skilled, un-skilled

Professional, big business, landlord, university teacher, class 1 IAS/services officer, lawyer	1
Trained, clerical, medium business owner, middle level farmer, teacher, maintenance (in charge), personnel manager	2
Skilled manual labourer, small business owner, small farmer	3
Semi-skilled manual labourer, marginal landowner, rickshaw driver, army jawan, carpenter, fitter	4
Unskilled manual labourer, landless labourer	5

19. What is your total household income per month?<br><br>***Please include income from all members who contribute to the household***	<3000 1<br>3000-10,000 2<br>10,001-20,000 3<br>20,001-30,000 4<br>30,001-40,000 5<br>40,001-50,000 6<br>>50,000 7<br>Refuse 8<br>Don't know 9	
20. Do you have a separate room for cooking (Kitchen)?	Yes 1<br>No 2	

21.What is the fuel used for cooking?<br>***If more than one source is used then note the source that is most commonly used***	Coal/charcoal/kerosene 1<br>Electricity/gas (LPG)/solar/CNG (IGL) 2<br>Wood/dung 3<br>Others 4	Others (specify) ________________
22.What is the source of drinking water used at home?<br>***If more than one source is used then note the source that is most commonly used***	Public source 1<br>Private source (Shared) 2<br>Private source (Own) 3<br>Bottled water 4<br>Purified tap water 5<br>Others 6	Others (specify) ________________
23.What is the toilet facility you use?	Public toilet 1<br>Shared toilet 2<br>Own flush toilet 3<br>Others 4	Others (specify) ________________
24.Which of the following do you own?<br>***[Yes=1; No=2]***	a. Television<br>b. Refrigerator<br>c. Washing machine<br>d. Microwave / OTG<br>e. Mixer-grinder<br>f. Mobile phone<br>g. DVD player<br>h. Computer<br>i. Car<br>j. Motor Cycle /Scooter<br>k. Bicycle	
25.Are you likely to move from your current residence within a year or two?	Yes 1<br>No 2<br>Don't know 3	
26.In case you move from current residence, whom can we contact to obtain your new contact address or telephone numbers?<br>***Take details of two different contacts***	Neighbour 1<br>Relative 2<br>Friend 3<br>Employer 4<br>No one to contact 5<br>Others 6<br>Specify ____________________________	**1st**<br>**2nd**
27.Name of the 1st contact person<br>First Name:<br>Middle name:<br>Last Name:		

28. Address of the 1st contact person	
29. Phone number (home, office, mobile) of 1st contact person	Home ___________(area code)______________(number)<br>Office ___________(area code)______________(number)<br>Mobile__________________________________(number)
30. Name of the 2nd contact person<br>First Name:<br>Middle Name:<br>Last Name:	
31. Address of the 2nd contact person	
32. Phone number (home, office, mobile) of 2nd contact person	Home ___________(area code)______________(number)<br>Office ___________(area code)______________(number)<br>Mobile__________________________________(number)

SECTION – 2: TOBACCO AND ALCOHOL CONSUMPTION, DIETARY HABITS, PHYSICAL ACTIVITY AND SLEEP

PART – A: TOBACCO USE

1. Have you ever used tobacco in any form (smoking, chewing, snuff, etc)?	Yes 1<br>No 2	**"2" go to Q. 8**
2. In what forms have you consumed tobacco?<br>***[Yes=1; No=2]***	a. In a smoking form<br>b. In a chewed form<br>c. In any other form (snuff, toothpaste etc)	
3. Do you currently* consume tobacco?<br>* within past 6 months	Yes 1<br>No 2	**"2" go to Q. 5**
4. If Yes, how often?<br>***[Regularly (≥once a week)= 1; Occasionally (<once a week)= 2; No=3; Not applicable=9]***	Smoking form<br>Chewed form	Any other form

5. Quantity and duration of use (for both current and past users)

Type of tobacco use / used	***Brand name***	***Duration of use***		***Usage per month*** ****Number smoked*** *****Number of times*** ******Approximate amount in gms***	***If you have stopped using any of the following products, time in months/years since you have stopped***	
		Years	***Months***		***Years***	***Months***
1. Cigarette*						
2. Beedi*						
3. Cigar*						
4. Hukka/Chelum/Pipe **						
5. Tobacco chewing***						
6. Pan with Zarda***						
7. Pan masala with zarda***						
8. Snuff**						
9. Gutkha***						
10. Others: Specify __________						

6. At what age did you first start smoking regularly? ***[Not applicable – write '99' in the box]***	**years**	
7. At what age did you first start consuming smokeless tobacco product regularly? ***[Not applicable – write '99' in the box]***	**years**	
8. Are you exposed to tobacco smoke from others regularly*? (e.g. at home, at workplace regularly, while travelling, any other place) * At least once a day in a week	Yes 1<br>No 2	**"2" go to PART B**
9. **If Yes**:<br>How many days a week*?<br>How much time during a day*?	HR : MIN (Please provide approximate time)	

PART – B: ALCOHOL USE

1. Have you ever used alcohol?	Yes 1<br>No 2	**"2" go to PART C**

2. How often do you use alcoholic beverages?<br><br>***Occasionally means less than once a week**	Currently using alcohol regularly 1<br>Currently using alcohol occasionally* 2<br>Used alcohol in the past (stopped more than 6 months ago) 3<br>Recently stopped alcohol (less than 6 months ago) 4<br>Never used alcohol 5	**"5" go to PART C**

3. History of alcohol use for both present and past users

Type of alcohol used	***Duration of use***		***Frequency of use per week***	***Quantity**** *in ml/peg per occasion*	**If stopped, since how long**	
	**Years**	**Months**			**Years**	**Months**
a) Local spirits eg. Desi, arrack, toddy etc						
b) Spirits eg. whisky, rum, brandy, gin, vodka						
c) Beer						
d) Wine						

** Conversion

1 small peg = 30 ml; 1 large peg = 60 ml; 1 extra large peg = 90 ml

1 glass of beer = approx. 325 ml

1 glass of wine = 100 ml

Please use local measures in calculating the total consumption (in ml per occasion)

PART – C: PHYSICAL ACTIVITY (International Physical Activity Questionnaire – short)

We are interested in finding out about the kinds of physical activities that people do as part of their everyday lives. I am going to ask you about the time you spent being physically active in the usual 7 days of a week. Please answer each question even if you do not consider yourself to be an active person. Think about the activities you do at work, as part of your house and yard work, to get from place to place, and in your spare time for recreation, exercise or sport.

Now, think about all the *vigorous* activities that you did in a usual 7 days. Vigorous activities make you breathe much harder than normal and may include heavy lifting, digging, aerobics, or fast bicycling. Think only about those physical activities that you do for at least 10 minutes at a time.

1. During **a usual 7 days**, on how many days did you do **vigorous** physical activities?	Days per week = 1<br>Refused (go to Q.3) = 2<br>Don't Know/Not Sure (go to 3) = 3<br>Don't do any activity (go to 3) = 4	
2. How much time did you usually spend doing **vigorous** physical activities on one of those days? Think only about those physical activities you do for at least 10 minutes at a time.	Hours/ Minutes per day = 1<br>Refused (go to Q.2a) = 2<br>Don't Know/Not Sure (go to 2a) = 3	: HR MIN

2a. **Interviewer probe:** If the respondent can't answer because the pattern of time spent varies widely from day to day, say, "I am interested in the average time for one of the days on which you do vigorous activity. Can you tell me how much time in total would you spend **over a usual 7 days** doing vigorous physical activities?"	Hours/ Minutes per day = 1<br>Refused = 2<br>Don't Know/Not Sure = 3	HR : MIN
**Now think about activities which take *moderate physical effort* that you did in a usual 7 days. Moderate physical activities make you breathe somewhat harder than normal. Do not include walking. Again, think about only those physical activities that you did for at least 10 minutes at a time.**		
3. During a **usual 7 days**, on how many days did you do **moderate** physical activities for at least 10 minutes?<br>Examples: carrying loads, bicycling at a regular pace, tennis, badminton, cricket, hand washing clothes, sweeping the floor, gardening, taking care of children less than three years old, washing cars, motorcycles, or scooters, walking home while carrying vegetables and groceries from market, climbing stairs (three floors or more), and grinding chutney on stone.	Days per week =1<br>Refused (go to Q.5) = 2<br>Don't Know/Not Sure (go to Q.5) = 3<br>Don't do any activity (go to Q.5) = 4	
4. How much time did you usually spend doing **moderate** physical activities on one of those days? Think only about those physical activities that you do for at least 10 minutes at a time.	Hours/ Minutes per day=1<br>Refused (Go To Q.4a) = 2<br>Don't Know/Not Sure (Go To Q.4a)=3	HR : MIN
4a. **Interviewer probe**: If the respondent can't answer because the pattern of time spent varies widely from day to day, or includes time spent in multiple jobs, say, "I am interested in the average time for one of the days on which you do moderate activity. Can you tell me what is the total amount of time you spent over a **usual 7 days** doing moderate physical activities?"	Hours/ Minutes per day=1<br>Refused = 2<br>Don't Know/Not Sure = 3	HR : MIN
**Now think about the time you spent walking in a usual 7 days. This includes at work and at home, walking to travel from place to place. Also include any walking that you do solely for recreation, sport, exercise, or leisure, for example, walking to the bus stop, to workplace, to the market for at least 10 minutes.**		
5. During a **usual 7 days**, on how many days did you **walk** for at least 10 minutes at a time? Think only about the walking that you do for at least 10 minutes at a time.	Days per week = 1<br>Refused (go to Q. 7) = 2<br>Don't Know/Not Sure (go to Q. 7) =3<br>Don't do any activity (go to Q. 7) = 4	
6. How much time did you usually spend **walking** on one of those days?	Hours/ Minutes per day (go to Q. 7) =1<br>Refused (go to Q.6a) = 2<br>Don't Know/Not Sure (go to Q6a) =3	HR : MIN
6a. **Interviewer probe**: If the respondent can't answer because the pattern of time spent varies widely from day to day say, "I am interested in the average time for one of the days on which you walk. Can you tell me what is the total amount of time you spent walking over **a usual 7 days**?"	Hours/ Minutes per week =1<br>Refused =2<br>Don't Know/Not Sure =3	HR : MIN
**Now think about the time you spent sitting on week days during a usual 7 days. Include time spent at work, at home, while doing course work, and during leisure time. This may include time spent sitting at a desk, visiting friends, reading or sitting or lying down to watch television, cutting vegetables, sewing and knitting, or time spent in teaching children, performing religious prayers, chatting with friends, talking on the phone, or working in front of the computer.**		

Participant ID

7. During a usual 7 days, how much time did you usually spend ***sitting*** on a **weekday**? Include time spent lying down (awake) as well as sitting. *(*Exclude sleeping at night)*	Hours/ Minutes per week day (go to Q. 8)=1<br>Refused (go to Q. 7a) = 2<br>Don't Know/Not Sure(go to Q7a) = 3	HR : MIN
7a**. Interviewer probe**: If the respondent can't answer because the pattern of time spent varies widely from day to day, say "I am interested in the average time per day spent sitting. Can you tell me what is the total amount of time you spent *sitting* last **Wednesday**?"	Hours/ Minutes on Wednesday = 1<br>Refused =2<br>Don't Know/Not Sure =3	HR : MIN
8. Additional comments		

PART – D: SLEEP (Sleep Heart Health Study; NHLBI)

1. How many hours of sleep do you usually get at night (or your main sleep period)?<br>**Average hours of sleep per night**	On weekdays / workdays<br>No. of hrs	On weekends<br>No. of hrs
2. During a usual week, how many times do you nap for 5 minutes or more? (Write "00" if the participant does not take any naps)	No. of times	

3. Please indicate how often you experience each of the following **(refer to codes below)**
[Never=1; Rarely (1/month or less)=2; Sometimes (2-4/month)=3; Often (5-15/month)=4; Almost always (16-30/month)=5]

A. Have trouble falling asleep
B. Wake up during the night and have difficulty getting back to sleep
C. Wake up too early in the morning and be unable to get back to sleep
D. Feel unrested during the day, no matter how many hours of sleep you had
E. Do not get enough sleep
F. Take sleeping pills or other medication to help you sleep

Questions 4 to 10 are about snoring and breathing during sleep. To answer these questions please consider what other have told you and what you know about yourself

4. Have you ever snored (now or any time in the past)?	Yes<br>No<br>Don't know	1<br>2<br>8	**"2", "8" go to Q.7**
5. How often do you snore now?	Do not snore anymore<br>Rarely (<1 night/week)<br>Sometimes (1-2 nights/week)<br>Frequently (3-5 nights/week)<br>Always or almost always(6-7nights/week)<br>Don't know	0<br>1<br>2<br>3<br>4<br>8	**"0" go to Q.7**
6. How loud is your snoring?	Only slightly louder than heavy breathing<br>About as loud as mumbling or talking<br>Louder than talking<br>Extremely loud-can be heard through a closed door<br>Don't know	1<br>2<br>3<br>4<br>8	

7. Based on what you have noticed or household members have told you, are there times when you stop breathing during your sleep?	Yes 1<br>No 2<br>Don't know 8	**"2", "8" go to Q.9**
8. How often do you have times when you stop breathing during your sleep?	Rarely (<1 night/week) 1<br>Sometimes (1-2 nights/week) 2<br>Frequently (3-5 nights/week) 3<br>Always or almost always(6-7nights/week) 4<br>Don't know 8	
9. Have you ever been told by a doctor that you had sleep apnoea (a condition in which breathing stops briefly during sleep)?	Yes 1<br>No 2<br>Don't know 8	**"1" go to Q.11**<br>**"2", "8" go to Q.10**
10. Have you ever been told by a doctor that you had some other sleep disorder?	Yes 1<br>No 2<br>Don't know 8	**"2", "8" go to Q.11**
10.A. If response is "yes" to the above question, please specify the disorder<br>____<br>____		
11. Do you usually use oxygen therapy (oxygen delivered by a mask or nasal cannula) during your sleep?	Yes 1<br>No 2	
12. During the past year how often have one or more members of your household been in or near the room where you have slept?	Never 1<br>Sometimes 2<br>Usually 3	

13. **What is the chance that you would doze off or fall asleep (not just "feel tired") in each of the following situations? (refer to the codes below)**

[No chance=1; Slight chance=2; Moderate chance=3; High chance=4]

*If you are never or rarely in the situation, please give your **best guess** for the situation*

A. Sitting and reading

B. Watching TV

C. Sitting inactive in a public place (such as a theatre or a meeting)

D. Riding as a passenger in a car for an hour without a break

E. Lying down to rest in the afternoon when circumstances permit

F. Sitting and talking to someone

G. Sitting quietly after a lunch

H. In a car, while stopped for a few minutes in traffic

I. At the dinner table

J. While driving

14. How often do you take aspirin or aspirin-containing medicines?	Never 1<br>Less often than once a week 2<br>Once or twice a week 3<br>Every other day (one day out of two) 4<br>Every day 5<br>Don't know 8	
15. Do you drive?	Yes 1<br>No 2	**"2" go to Part E**

16. **If the response to the above question is "yes" please answer the following questions, else go to Part-E (Diet)**

A. No. of years of driving

B. How often do you drive?
[Everyday=1; sometimes=2; rarely/never=3]

C. Since you began driving, how many accidents have you had while you were the driver?

D. How many accidents have you had in the last year while you were the driver?

PART – E: DIET

1. Are you a vegetarian?	Yes 1<br>No 2	
2. Do you take eggs?	Yes 1<br>No 2	
3. Are you on any special diet?	Yes 1<br>No 2	**"2" go to Q.6**
4. If ***YES***, what diets are you currently following<br>***[Yes = 1; No = 2]***	Diabetic diet<br>Low fat diet<br>High fibre diet<br>Low salt diet<br>Weight reducing diet<br>Others (Specify)<br>___________________________	

5. Since how many years are you on this special diet? Yrs Mnts [Enter the the longest duration]

6. How frequently do you use reheated oil?	Every day 1<br>Every other day (one day out of two) 2<br>Once or twice a week 3<br>Less often than once a week 4<br>Never 5	

5. In the past one year, how often have you consumed foods from the following food groups? **[write the frequency of consumption in the appropriate column]**

Sl. No.	Food groups	Consumed never/less than once /month [√]	Consumed monthly	Consumed weekly	Consumed daily
**1**	**Meats**				
**2**	**Poultry**				
**3**	**Organ meats**				

**4**	**Fish**				
**5**	**Shell fish and crustaceans**				
**6**	**Eggs**				
**7**	**Milk and milk products**				
**8**	**Milk based desserts**				
**9**	**Deep fried foods: western style**				
**10**	**Deep fried foods: desi style**				
**11**	**Western style desserts/sweet snacks**				
**12**	**Mithai**				
**13**	**cold beverages**				
**14**	**Fruits (1)**				
**15**	**Fruits (2)**				
**16**	**Fruit juices**				
**17**	**Nuts/seeds**				
**18**	**Leafy greens**				
**19**	**Other raw vegetables**				
**20**	**Legumes and pulses**				
**21**	**Use of pickles, pickled foods**				
**22**	**Other cooked vegetables**				
**23**	**Refined cereals with less fibre**				
**24**	**Whole grain**				
**25**	**Tea consumption**				
**26**	**Coffee consumption**				

**Annex for food groups [showing items in each group]**
**Meat** [lamb, mutton, goat, veal, rabbit, beef, pork; their curries]
**Poultry** [chicken, turkey, duck, pheasant, quail; their curries]
**Organ meats** [liver, kidney, brain, spleen, heart and sausages nihari, paya]
**Fish** [fresh-water and sea-water fish; preserved fish such as salted fish, canned fish, dried fish]
**Shell fish and crustaceans** [crab, squid, prawns, molluscs, caviar]
**Eggs** [Includes preserved eggs, duck eggs]
**Milk and milk products:** [milk, yogurt, cheese, curd, raita, lassi, milk based drinks]
**Milk based desserts** [custard, khoya, firni, kheer, milk puddings, rasgullah/rasmalai, ice creams]all milk based desserts
**Deep fried foods: western style** [french fries, potato chips, onion rings, chicken nuggets]
**Deep fried foods: desi style** [samosas, papad, pakoras, sev, namak paray, egg rolls, poori, kachori]
**Western style desserts/sweet snacks** [cakes; pies; chocolate; candy; biscuits]
**Mithai** [burfi/ladoo; gulab jamun; halwa; shameia, mohalabeia]
**Cold beverages** [carbonated beverages, sherbets, and other soft drinks]
**Fruits (1)** [strawberries, pine apples, jumbo berries (jamuns), apples]
**Fruits (2) all seasonal fruits except the ones above**
**Fruit juices** [any type, homemade, purchased, fresh, frozen]
**Nuts/seeds** [Includes peanuts, almonds, sunflower seeds, cashews, walnuts]
**Leafy greens** [all fresh leafy green vegetables: spinach, mustard or turnip greens; asparagus either raw or cooked]
**Other raw vegetables** [any raw vegetables not included in the preceding categories]
**Legumes and pulses** [includes all daals, chickpeas, lentils]
**Use of pickles, pickled foods** [achar, chutneys, pickled vegetables etc]
**Other cooked vegetables** [any cooked vegetables not included in the preceding categories]
**Refined cereals with less fibre** [boiled rice, fried rice, biryani, pulao, idli, dosa, semolina,sago, pearl barley, pasta, sheermal, taftan, white bread slice]
**Whole grain (cereal dished with more fibre)** [Roti made with whole meal flour, brown rusk, whole wheat porridge, bread slice whole meal/brown]
**Tea consumption** [black tea, coffee with and without milk and sugar and any other tea]
**Coffee consumption** [coffee with and without milk and/sugar]

SECTION – 3: FEMALE REPRODUCTIVE HISTORY

THIS SECTION IS TO BE FILLED ONLY FOR THE FEMALE PARTICIPANTS, FOR MALE PARTICIPANTS SKIP THIS SECTION AND GO TO SECTION – 4.

1. Number of pregnancies so far?<br>***Not Applicable = 99***	

Participant ID

2. At what age did you start menstruating?	Years	
3. Are you having menstrual cycles?	Yes 1<br>No 2	**"1" go to Q. 6**
4. If '**No'** what is the reason?	Pregnancy 1<br>Lactation 2<br>Natural menopause 3<br>Surgical menopause 4<br>Other reasons(specify) 5	***Others, specify***____________________<br>_______________
5. If postmenopausal, since how long?	Years Months	

6. Hormonal drugs or oral contraceptive pills?<br>***[Yes = 1; No = 2]***	Ever used in the past		If Yes, duration in years/month<br>Yrs Mnts
	Currently using		If Yes, duration in years/month<br>Yrs Mnts

SECTION – 4: QUALITY OF LIFE (EQ-5D) © 1990 EuroQol Group. EQ-5D™ is a trade mark of the EuroQol Group.

By writing a code from the options in the box, please indicate which statements best describe your own state of health today.

1. Mobility	I have no problems in walking about=1<br>I have some problems in walking about=2<br>I am confined to bed=3	
2. Self-Care	I have no problems with self-care=1<br>I have some problems washing or dressing myself=2<br>I am unable to wash or dress myself=3	
3. Usual Activities<br>*(e.g. work, study, housework, family or leisure activities)*	I have no problems with performing my usual activities=1<br>I have some problems with performing my usual activities=2<br>I am unable to perform my usual activities=3	
4. Pain/ Discomfort	I have no pain or discomfort=1<br>I have moderate pain or discomfort=2<br>I have extreme pain or discomfort=3	
5. Anxiety/ Depression	I am not anxious or depressed=1<br>I am moderately anxious or depressed=2<br>I am extremely anxious or depressed=3	

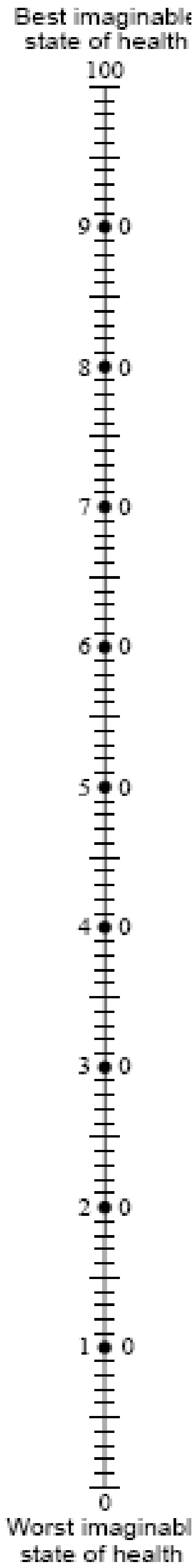

To help people say how good or bad their state of health is, we have drawn a scale (rather like a thermometer) on which the best state you can imagine is marked 100 and the worst state you can imagine is marked 0.

I would like you to indicate on this scale how good or bad your own health is today, in your opinion. Please do this by drawing a line from the box below to whichever point on the scale indicates how good or bad your state of health is today.

Your own
state of health
today

Participant ID ☐☐☐☐☐

**SECTION – 5: MEDICAL HISTORY**		
**PART-A: CARDIOMETABOLIC DISEASES AND THEIR RISK FACTORS**		
1. Have you ever been told by a doctor that you have any of the following diseases?<br><br>***[Yes =1; No =2; Don't know=3]***	Hypertension (High blood pressure)<br>Diabetes (High Blood Sugar)<br>Hyperlipidemia (High Cholesterol)<br>Heart Disease<br>Stroke (Paralytic Attack)<br>Chronic Kidney Disease	☐<br>☐<br>☐<br>☐<br>☐<br>☐
**Exclude pregnancy induced Hypertension and High Blood Sugar*<br>*If the answer is* ***'YES'*** *to any of the choices in Q. 1, then go to PART – B* ***'OTHERWISE'*** *skip the entire section and go to PART-C.*		
**PART - B: DISEASE SPECIFIC QUESTIONS**		
**I. HYPERTENSION (High Blood Pressure)**<br>***Fill this section if the answer for high blood pressure is "YES" in PART - A, Q.1.***		
a. Since how many years have you had high blood pressure?		Duration in years/month<br>Yrs ☐☐ Mnts ☐☐
b. What treatment are you taking for it currently?<br>***[Yes=1; No=2]***<br>****Traditional medicine / therapy include yoga, ayurveda, unani, homeopathy, Tibetan, naturopathy, meditation***	Prescribed dietary modifications<br>Prescribed physical exercise<br>Traditional medicine / therapy*<br>Allopathic drugs (English / modern)<br>None	☐<br>☐<br>☐<br>☐<br>☐
c. How regular are you in taking your medicines?	Taking Regularly 1<br>Forget to take occasionally 2<br>Take medicines only when I feel the blood pressure is high 3<br>Discontinued for more than a month at a time 4<br>Never taken any medication 5	☐
If "4" go Q.d otherwise go to Q.e.		
d. What is the reason for discontinuation?	Cannot afford 1<br>Cannot tolerate 2<br>I have recovered 3<br>No reason 4<br>Don't remember 5<br>Others (specify) 6	☐<br>***Others, specify***<br>________________
e. Do you think your blood pressure is under good control?	Yes 1<br>No 2<br>Don't Know 3	☐

f. Does your doctor say that your blood pressure is under good control?	Yes 1<br>No 2<br>Don't Know 3	
g. What was your last blood pressure recording (when your doctor checked you)? = 1<br>Don't know = 2<br>Can't remember = 3	____________(systolic) / _____________(diastolic) mmHg	
h. When was the last time you consulted your doctor?	Less than 1 month 1<br>More than 1 month 2<br>More than 3 months 3<br>Less than 6 months 4<br>More than 6 months 5	
i. Do you have medical records or prescriptions related to high blood pressure?	Yes 1<br>No 2<br>Don't Know 3	
*j. If the answer is **YES,** ask the participant to show the medical records and note the diagnosis below*		
k. Note the recorded blood pressure from the most recent medical record / prescription<br>____________(systolic) / _____________(diastolic) mmHg		
**II. DIABETES**<br>Fill this section if the answer for high blood sugar is **"YES"** in PART-A, Q.1		
a. For how long have you had high blood sugar / diabetes?	Duration in years/month<br>Yrs Mnts	
b. What treatment are you taking for it currently?<br>***[Yes=1; No=2]***<br>****Traditional medicine / therapy include yoga, ayurveda, unani, homeopathy, Tibetan, naturopathy, meditation***	Prescribed dietary modifications<br>Prescribed physical exercise<br>Traditional medicine / therapy*<br>Allopathic drugs (English / modern)<br>None	
c. How regular are you in taking your medicines?	Taking Regularly 1<br>Forget to take occasionally 2<br>Take medicines only when I feel the blood sugar is high 3<br>Discontinued for more than a month at a time 4<br>Never taken any medication 5	
If "4" go Q.d otherwise go to Q.e.		

Participant ID

d. What is the reason for discontinuation?	Cannot afford 1<br>Cannot tolerate 2<br>I have recovered 3<br>No reason 4<br>Don't remember 5<br>Others (specify) 6	***Others, specify***<br>_________________________
e. Do you think your diabetes/high blood sugar is under good control?	Yes 1<br>No 2<br>Don't Know 3	
f. Does your doctor say that your diabetes /high blood sugar is under good control?	Yes 1<br>No 2<br>Don't Know 3	
g. What was your fasting blood sugar and after meal blood sugar when you got it checked last time?	Fasting __________________mg/dl<br>After meal __________________mg/dl	
h. When was the last time you consulted your doctor?	Less than 1 month 1<br>More than 1 month 2<br>More than 3 months 3<br>Less than 6 months 4<br>More than 6 months 5	
i. Do you have medical records or prescriptions related to diabetes/high blood sugar?	Yes 1<br>No 2<br>Don't Know 3	

j. *If the answer is **YES,** ask the participant to show the medical records and note the diagnosis below*

k. ***Note the recorded fasting blood sugar and after meal blood sugar level from the most recent medical record / prescription***

Fasting____________________________mg/dl

After meal____________________________mg/dl

Also complete PART – D

Participant ID

III. HYPERLIPIDEMIA or High Blood Cholesterol<br>Fill this section if the answer for high blood cholesterol is "yes" in PART-A, Q.1		
a. For how long have you had high blood cholesterol?	Duration in years/month<br>Yrs Mnts	
b. What treatment are you taking for it currently?<br>***[Yes=1; No=2]***<br>****Traditional medicine / therapy include yoga, ayurveda, unani, homeopathy, Tibetan, naturopathy, meditation***	Prescribed dietary modifications<br>Prescribed physical exercise<br>Traditional medicine / therapy*<br>Allopathic drugs (English / modern)<br>None	
c. How regular are you in taking your medicines?	Taking Regularly 1<br>Forget to take occasionally 2<br>Take medicines only when I feel the blood cholesterol is high 3<br>Discontinued for more than a month at a time 4<br>Never taken any medication 5	
If "4" Q.d otherwise go to Q.e		
d. What is the reason for discontinuation?	Cannot afford 1<br>Cannot tolerate 2<br>I have recovered 3<br>No reason 4<br>Don't remember 5<br>Others (specify) 6	***Others, specify***<br>_____________________
e. Do you think your cholesterol is under good control?	Yes 1<br>No 2<br>Don't Know 3	
f. Does your doctor say that your cholesterol is under good control?	Yes 1<br>No 2<br>Don't Know 3	
g. What was total cholesterol level when you last checked it?	____________________________ mg/dl	
h. When was the last time you consulted your doctor?	Less than 1 month 1<br>More than 1 month 2<br>More than 3 months 3<br>Less than 6 months 4<br>More than 6 months 5	

i. Do you have medical records or prescriptions related to high blood cholesterol?	Yes 1<br>No 2<br>Don't Know 3	
*j. If the answer is **YES,** ask the participant to show the medical records and note the diagnosis below*		
k. Note the recorded total cholesterol from the most recent medical record / prescription		
**IV. HEART DISEASE**<br>**Fill this section if the answer for heart trouble is "YES" in PART-A, Q.1**		
a. When did you first come to know that you have heart disease?	<1 year 1<br>1-5 years 2<br>>5 years 3	
b. What did the doctor say it was?	Heart attack 1<br>Angina 2<br>Heart failure 3<br>Valve disease 4<br>Hole in the heart 5<br>Others* 6<br>Not informed about the nature of the problem 7	Use separate boxes for more than one option<br>***Others, specify***<br>______________________
If "1" go to Q.c otherwise go to Q. g.		
c. At what age did you have your 1st heart attack?	Years	
d. Were you hospitalized for treatment?	Yes 1<br>No 2	
e. Did you have any repeat attacks	Yes 1<br>No 2	
f. Were you hospitalized for the subsequent attacks	Yes 1<br>No 2	

g. What treatment are you taking for heart disease currently? ***[Yes=1; No=2]*** ****Traditional medicine / therapy include yoga, ayurveda, unani, homeopathy, Tibetan, naturopathy, meditation***	Prescribed dietary modifications<br>Prescribed physical exercise<br>Traditional medicine / therapy*<br>Allopathic drugs (English / modern)<br>None	
h. How regular are you in taking your medicines?	Taking Regularly 1<br>Forget to take occasionally 2<br>Take medicines only when I feel unwell 3<br>Discontinued for more than a month at a time 4<br>Never taken any medication 5	
If "4" go to Q.i question otherwise go to Q.j.		
i. What is the reason for discontinuation?	Cannot afford 1<br>Cannot tolerate 2<br>I have recovered 3<br>No reason 4<br>Don't remember 5<br>Others (specify) 6	***Others, specify***<br>______________
j. When was the last time you consulted your doctor?	Less than 1 month 1<br>More than 1 month 2<br>More than 3 months 3<br>Less than 6 months 4<br>More than 6 months 5	
k. Do you have medical records or prescriptions related to heart trouble?	Yes 1<br>No 2<br>Don't Know 3	
l. ***If the answer is 'YES', ask the participant to show the medical records and note the diagnosis below***		

**V.**	**STROKE (Paralytic attack)**<br>**Fill this section if the answer for stroke (paralytic attack) is "yes" in PART-A, Q.1**		
a.	What was your age when you had stroke (Paralytic attack)?	Years	
b.	Is there a residual disability in any part of the body?	Yes 1<br>No 2	
c.	If **'YES'**, does it involve the following?<br>***[Yes=1; No=2]***	Paralysis of leg/foot<br>Paralysis of arm/hand<br>Weakness of leg/foot<br>Weakness of arm/hand<br>Defect of speech<br>Defect of vision<br>Urinary incontinence<br>Any other weakness (specify)______________________	
d.	Are you advised to continue any medication after your paralytic attack?	Yes 1<br>No 2	
e.	If **YES**, how regular are you in taking your medicines?	Taking Regularly 1<br>Forget to take occasionally 2<br>Take medicines only when I feel unwell 3<br>Discontinued for more than a month at a time 4<br>Never taken any medication 5	
If "4" go to Q.f otherwise go to Q.g.			
f.	What is the reason for discontinuation?	Cannot afford 1<br>Cannot tolerate 2<br>I have recovered 3<br>No reason 4<br>Don't remember 5<br>Others (specify) 6	***Others, specify***<br>________________
g.	When was the last time you consulted your doctor?	Less than 1 month 1<br>More than 1 month 2<br>More than 3 months 3<br>Less than 6 months 4<br>More than 6 months 5	
h.	Do you have medical records or prescriptions related to Stroke?	Yes 1<br>No 2<br>Don't Know 3	

i. *If the answer is **YES**, ask the participant to show the medical records and note the diagnosis below*		
**VI. CHRONIC KIDNEY DISEASE** **Fill this section if the answer for chronic kidney disease is "YES" in PART-A, Q.1**		
a. At what age were you diagnosed with chronic kidney disease?	Years	
b. What treatment are you taking for it currently? ***[Yes=1; No=2]*** ****Traditional medicine / therapy include yoga, ayurveda, unani, homeopathy, Tibetan, naturopathy, meditation***	Prescribed dietary modifications<br>Prescribed physical exercise<br>Traditional medicine / therapy*<br>Allopathic drugs (English / modern)<br>None	
c. How regular are you in taking your medicines?	Taking Regularly 1<br>Forget to take occasionally 2<br>Take medicines only when I feel unwell 3<br>Discontinued for more than a month at a time 4<br>Never taken any medication 5	
If "4" go Q.d otherwise go to Q. e.		
d. What is the reason for discontinuation?	Cannot afford 1<br>Cannot tolerate 2<br>I have recovered 3<br>No reason 4<br>Don't remember 5<br>Others (specify) 6	***Others, specify*** ___________
e. When was the last time you consulted your doctor?	Less than 1 month 1<br>More than 1 month 2<br>More than 3 months 3<br>Less than 6 months 4<br>More than 6 months 5	
f. Do you have medical records or prescriptions related to chronic kidney disease?	Yes 1<br>No 2<br>Don't Know 3	
g. *If the answer is **YES,** ask the participant to show the medical records and note the diagnosis below*		

Participant ID

PART - C: ANGINA, PERIPHERAL VASCULAR DISEASE AND HEART FAILURE		
**I. ANGINA**		
a. Do you have any of the following symptoms?<br>***[Yes=1; No=2]***	Palpitation<br>Chest pain<br>Breathlessness<br>Fatigue/weakness<br>Chest discomfort/heaviness/pressure	**"2" for all, skip to Q. m**
b. With exertion*, have you ever had any of the following symptoms in and around the chest, arms, shoulders, neck, lower jaw, abdomen or upper back?<br>*walking fast, climbing stairs, lifting weights, etc<br>***[Yes=1; No=2]***	Pain<br>Heaviness<br>Pressure<br>Discomfort<br>Numbness	**"2" for all, skip to Q. d**
c. Where did you mostly feel the (symptoms noted in Q.b)?<br>***[Yes=1; No=2]***<br>(Please specify the location from the numbered diagram below)<br>***Additional numbers: back of chest = 11, back of neck = 12)***	***Symptom***<br>Pain<br>Heaviness<br>Discomfort<br>Numbness	***Location***

Right

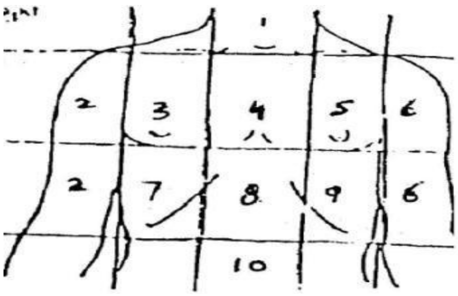

Left

d. Do you feel any of the above symptoms anywhere else?	Yes 1<br>No 2<br><br>*If* ***'YES'****, specify:*<br>Symptom:_________________<br>Location:__________________	
Fill Q.e to Q.l only if you have noted "1" for any of the symptoms in Q.b and Q.c, OTHERWISE GO TO Q.m		

e. Do you get the above symptoms, or breathlessness, or palpitation when you walk uphill or climb steps or walking fast?	Yes 1<br>No 2<br>Never walk uphill/hurry 3	
f. Do you get it when you walk at an ordinary pace on the level ground?	Yes 1<br>No 2	
g. Do you get a similar symptoms while you are resting or after a meal?	Yes 1<br>No 2	
h. What do you usually do if you get it while you are exerting?	Stop 1<br>Slow down 2<br>Carry on at the same pace 3	
i. Does it go away if you slow down or stand still?	Yes 1<br>No 2	**"2" go to Q.k**
j. If `***YES'*** to Q. i, how soon does it usually go away?	< 3 mins 1<br>3-20 mts 2<br>>20 mts 3	
k. Do you take usually a pill under the tongue to get relief?	Yes 1<br>No 2	**"2" go to Q.m**
l. If `***YES'***, how soon does it go away?	< 2mts 1<br>2-5 mts 2<br>6-10 mts 3<br>>10 mts 4	
m. Have you ever had a severe pain or discomfort in the front of your chest lasting for half an hour or more?	Yes 1<br>No 2	**"2", go to the next section**
n. If **'*YES'*,** was the pain or discomfort accompanied by - ***[Yes=1; No=2]***	Cold clammy skin<br>Breathing difficulty<br>Sweating	
o. How old were you when you had such a severe pain in the chest?	Years	
p. How many of these attacks have you had?		
q. Have you ever had an ECG done?	Yes 1<br>No 2	
r. Did you see a doctor because of the pain?	Yes 1<br>No 2	

II. PERIPHERAL VASCULAR DISEASE		
a. Do you get pain in either leg on walking?	Yes 1<br>No 2	**"2" go to the Part III**
b. If **YES**, in what part of your leg do you feel it?	Pain includes calf / calves 1<br>Pain does not include calf/claves 2	**"2" go to the Part III**
c. Do you get it if you climb stairs or walking fast?	Yes 1<br>No 2<br>Not Applicable 3	**"2" go to the Part III**
d. Do you get it if you walk at an ordinary pace on the level ground?	Yes 1<br>No 2	
e. Does the pain ever disappear while you are still walking?	Yes 1<br>No 2	**"1" go to the Part III**
f. What do you do if you get it when you are walking?	Stop or slacken pace 1<br>carry on 2	**"2" go to the Part III**
g. What happens to it if you stand still?	Relieved 1<br>Not Relieved 2	**"2" go to the Part III**
h. If relieved, how soon?	10 minutes or less 1<br>more than 10 minutes 2	
**III. HEART FAILURE**		
a. Are you unable to walk due to physical disability?	Yes 1<br>No 2	**"1" skip to Q. e**
b. Do you ever get short of breath while walking with other people of your own age on level ground?	Yes 1<br>No 2	
c. On walking uphill or upstairs, do you get more breathless than people of your own age?	Yes 1<br>No 2	**"2" go to Q. e**
d. Do you ever have to stop walking because of breathlessness?	Yes 1<br>No 2	
e. In the past years have you at any time awoken at night by an attack of shortness of breath?	Yes 1<br>No 2	**"2" go to Q. g**

Participant ID

f.	For how long have you had this problem?	Less than one year 1<br>More than one year 2	
g.	Do you have swelling in your ankles?	Yes 1<br>No 2	
h.	Have you been told by your doctor at any time that you are suffering from any lung disease (COPD, Asthma,etc)?	Yes 1<br>No 2	
i.	Do you have a cardiac device?	Yes 1<br>No 2	
j.	If "**YES**", name the device	Standard pacemaker 1<br>Implantable Cardioverter defibrillator (ICD) 2<br>Cardiac resynchronisation therapy device with defibrillator (CRT-D) 3	

PART - D: COMPLICATIONS

Complete the following sections only if you have filled the "diabetes section" (2) in PART-B

I. FOOT ULCERS AND AMPUTATION

a.	Have you ever had a non healing ulcer/sore in the foot that took more than 4 weeks to heal?	Yes 1<br>No 2	
b.	Do you walk around bare foot?	Yes 1<br>No 2	
c.	Have you had an amputation?	Yes 1<br>No 2	**"2" go to Part II**
d.	If '**YES'** When?	years before	(or) months before
e.	Level of amputation	Toe 1<br>Below ankle 2<br>Below knee 3<br>Above Knee 4	
f.	What was the cause for amputation?	Injury 1<br>Diabetes 2<br>Infection 3<br>Other 4	Others specify ____________________
g.	Do you have medical records or prescriptions?	Yes 1<br>No 2<br>Don't Know 3	
h.	If the answer is '**YES'**, ask the participant to show the medical records and note the *diagnosis below*		

**II. EYES**		
a. Do you have difficulty with your eyesight other than your ordinary power glasses (spectacles)?	Yes 1<br>No 2	**"2" skip the section**
b. If '**YES**', were you told that your poor eyesight is due to complications of diabetes?	Yes 1<br>No 2	**"2" skip the section**
c. If '**YES**', what was the diagnosis?		
d. Have you undergone laser therapy (Photocoagulation) at anytime	Yes 1<br>No 2	
e. Do you have medical records or prescriptions?	Yes 1<br>No 2<br>Don't know 3	
f. If the answer is **YES**, ask the participant to show the medical records and note the diagnosis below		

**PART – E: RESPIRATORY DISEASE**	
1. In the past 12 months, have you had chronic **cough** and chronic mucous production on **most** days or nights of the week (during at least three months in a row)? ***[Yes=1; No=2]***<br>**Cough** means cough even when you are not suffering from cold<br>**Most** means at least 4 days or nights per week	
a. If '**YES**' i. How many episodes of such cough have you had in the past 12 months?<br>ii. Have you suffered from any infections that required medical attention in the past 12 months? ***[Yes=1; No=2]***<br>iii. How many times did you seek medical attention in the past 12 months?	
2. Have you seen a doctor or health practitioner for a chest infection (excluding TB) in the past 12 months? ***[Yes=1; No=2]***	
a. If '**YES**' i. How many episodes in the past 12 months?<br>ii. How many were doctor-diagnosed?<br>iii. For how long have you had such infection?<br>iv. Did you take antibiotics for these infections?<br>***[Yes=1; No=2; Don't know=3]***	Yrs Mnts
3. Have you been hospitalized for a chest infection/pneumonia in the past 12 months? ***[Yes=1; No=2]***	
a. If '**YES**', Length of stay	days<br>weeks<br>months

4. Do you currently suffer from asthma?

[Yes=1; No=2]

a. IF '**YES**'

i. How many attacks of asthma have you had in the past 12 months?

ii. Have you suffered from any infections that required medical attention in the past 12 months? ***[Yes=1; No=2]***

iii. How many times did you seek medical attention in the past 12 months?

5. Have you ever been diagnosed with TB in past 5 years?

[Yes=1; No=2; Don't remember=3]

PART – F: FAMILY HISTORY

1. Has anyone in your family suffered from any of the following diseases, **before the age of 60 years**?

[Yes=1; No=2; Don't know=3]

High blood pressure

Heart disease*

Diabetes mellitus (High Blood Sugar)

Stroke (paralytic attack)

***Angina/ heart attack/heart failure**

2. Fill the table below

**Relationship to the family member**	**Disease condition (refer to the codes below)***	**Age at diagnosis (in years)**	**If dead, age at which the family member died**
Father			
Mother			
Son			
Daughter			
Paternal Grandfather			
Paternal Grandmother			
Maternal Grandfather			
Maternal Grandmother			
Brother			
Sister			
Paternal uncle			
Paternal aunt			
Maternal uncle			
Maternal aunt			
**For others, please write the relationship to the participant and provide the required details below**			

***Disease condition: Diabetes = 1, heart disease = 2, high blood pressure = 3, Stroke = 4**

Participant ID

SECTION – 6: TREATMENT HISTORY AND EXPENDITURES

PART A: OUTPATIENT

1. Are you undergoing treatment as an out-patient for any of the following reasons? ***[Yes=1; No=2]***	Heart disease	
	Stroke	
	Diabetes	
	Diabetic complications (infections, retinopathy, nephropathy, etc.)	
	High blood pressure	
	Chronic Kidney disease	

*If the answer to any of the above is **"YES"** go to the next section **OTHERWISE** skip to PART B*

In the following questions ask the details of treatment and cost only for the last 12 months

2. How many times did do you visit a health facility/doctor/therapist in past 12 month?			
3. Type of health facility/doctor/therapist	Government	1	***Others, specify*** ___________
	Private	2	
	Charity	3	
	Others	4	

4. List the expenditures incurred towards the above mentioned conditions (Q.1) separately in each table

4.i. Disease _______________________

Nature of expenditure	Frequency	Amount spent in Rs per visit/ test/remuneration to home nurse or carer
Visit to Doctor (fees)		
No. of months home nurse / carer was hired		
Tests		
Physical or occupational rehabilitation		
Others (Specify)________________________________		
Medications ***(average amount spent in last 12 months for the above mentioned condition)***		
**Total expenditure in past 12 months**		

4.ii. Disease ________________________

Nature of expenditure	Frequency	Amount spent in RS per visit / test/remuneration to home nurse or carer
Visit to Doctor (fees)		
No. of months home nurse / career was hired		
Tests		
Physical or occupational rehabilitation		
Others (Specify)________________________________		
Medications ***(average amount spent in last 12 months for the above mentioned condition)***		
**Total expenditure in past 12 months**		

4.iii. Disease ________________________

Nature of expenditure	Frequency	Amount spent in RS per visit/ test/remuneration to home nurse or carer
Visit to Doctor (fees)		
No. of months home nurse / career was hired		
Tests		
Physical or occupational rehabilitation		
Others (Specify)________________________________		
Medications ***(average amount spent in last 12 months for the above mentioned condition)***		
**Total expenditure in past 12 months**		

4.iv. Disease ________________________

Nature of expenditure	Frequency	Amount spent in RS per visit/ test/remuneration to home nurse or carer
Visit to Doctor (fees)		
No. of months home nurse / career was hired		
Tests		
Physical or occupational rehabilitation		
Others (Specify)________________________________		
Medications ***(average amount spent in last 12 months for the above mentioned condition)***		
**Total expenditure in past 12 months**		

Participant ID

5. Did you get any reimbursement from insurance?	Yes 1<br>No 2<br>Don't know 3<br>Don't have any insurance 4	
6. If **YES**, of the above mentioned expenditure how much was reimbursed (in RS)?	RS _______________	
7. Time taken to reach the health facility/doctor/therapist?	HR : MIN	
8. Transport cost to visit the above mentioned health facility/doctor/therapist*	RS ________________	
***If the participant has a private vehicle, ask him to give you an estimate of the amount spent on fuel to travel**		
9. Average time spent at health facility	HR : MIN	
10. Are you getting proper medical attention? ***[Yes=1; No=2]***		
10.a. If **"No"** What has prevented you from getting medical attention?	Not available 1<br>No one to help me get there 2<br>Too far 3<br>Too expensive 4<br>Don't want to spend money 5<br>Complicated procedures for care seeking 6<br>Too long a wait 7<br>Too sick to make the trip 8<br>Do not trust medical care 9<br>Do not know where to go 10<br>Others (Specify) 11	If other, specify ________________
11. How did you pay for your treatment and visits? ***[Yes=1; No=2]***	Own saving<br>Family members paid<br>Employer paid<br>Borrowed from friend, relatives & employer<br>Borrowed from bank<br>Sold house, land or other assets<br>Health insurance<br>Others (specify)________________	
12. On an average what proportion of money in percentage (%) did you spent from the above mentioned source for your treatment and visits?	Own saving<br>Family members paid<br>Employer paid<br>Borrowed from friend, relatives & employer<br>Borrowed from bank<br>Sold house, land or other assets<br>Health insurance<br>Others (Specify)____________________	__________%<br>__________%<br>__________%<br>__________%<br>__________%<br>__________%<br>__________%<br>__________%

Participant ID

PART B: INPATIENT		
1. Were you hospitalized for any illness in the **past 12 months**?	Yes 1<br>No 2<br>Don't remember 3	**"2 & 3" go to Q.4**
2. If **YES**, how many times?		
3. Were you admitted for any of the following reasons?<br><br>***[Yes=1; No=2]***	Heart disease<br>Stroke<br>Diabetes<br>Diabetic complications (infections, retinopathy, nephropathy, etc.)<br>High blood pressure<br>Chronic Kidney disease	
4. Have you undergone any surgical procedure in the **past 12 months**?	Yes 1<br>No 2<br>Don't remember 3	**"2 & 3" go to Q.6**
5. If yes, what was the procedure?<br><br>***[Yes=1; No=2]***	Revascularisation / bypass<br>Valve repair/replacement<br>Pacemaker<br>Amputation<br>Abscess<br>Renal transplantation<br>Heart transplant<br>Retinal photocoagulation<br>Others (Specify______________________)	
6. Do you have medical records related to hospitalization / surgical procedure?	Yes 1<br>No 2	

*If the answer is **YES,** ask the participant to show the medical records and note the diagnosis in a chronological order separately for hospitalisation due to illness and surgical procedures mentioned above in the space provided below*

Hospitalisation

____

____

____

____

____

Surgical procedure

Comments

PART C: HOSPITALISATION COST
Fill this section only if the participant has undergone hospitalisation due to illness or procedure mentioned in question 3 and 5 of part B, otherwise end the interview and thank the participant. ***For each hospitalisation note the following details, starting with the first hospitalisation in past 12 months. If the number of hospitalisation is more than three then use a second form to complete the history.***

Sl. No	Questions	1	2	3
1	When were you hospitalized?	MM YYYY	MM YYYY	MM YYYY
2	How many days did you stay in the hospital?	Days	Days	Days
3	Type of hospital? ***[Yes=1; No=2]***	Government<br>Private<br>Charity<br>Other	Government<br>Private<br>Charity<br>Other	Government<br>Private<br>Charity<br>Other
4	Name of hospital (Address)			

5	What type of treatment/procedure/ surgery did you undergo?<br><br>***(Cross-check with the medical records and information in PART-A)***<br><br>***[Yes=1; No=2]***	Medicines<br>Thrombolysis<br>Angiogram<br>Angioplasty<br>Bypass surgery<br>Brachytherapy<br>Pacemaker<br>Heart transplant<br>Amputation<br>Echocardiography<br>Neuro-imaging<br>Dialysis<br>Kidney-transplant<br>For observation<br>Other procedure<br>Specify ___________	Medicines<br>Thrombolysis<br>Angiogram<br>Angioplasty<br>Bypass surgery<br>Brachytherapy<br>Pacemaker<br>Heart transplant<br>Amputation<br>Echocardiography<br>Neuro-imaging<br>Dialysis<br>Kidney-transplant<br>For observation<br>Other procedure<br>Specify ___________	Medicines<br>Thrombolysis<br>Angiogram<br>Angioplasty<br>Bypass surgery<br>Brachytherapy<br>Pacemaker<br>Heart transplant<br>Amputation<br>Echocardiography<br>Neuro-imaging<br>Dialysis<br>Kidney-transplant<br>For observation<br>Other procedure<br>Specify _________
6	Total amount spent on treatment (hospitalisation expenses + medicines purchased during the stay)	Rs ________________	Rs ________________	Rs ________________
7	Number of days attendant stayed with you in the hospital	Days	Days	Days
8	Cost of attendant's stay (include food accommodation and travel)	Rs _______________	Rs ____________	Rs ____________
9	Distance from home to hospital?	Kms	Kms	Kms
10	Cost of travel from home to hospital (excluding ambulance cost, if any)	Rs __________________	Rs __________________	Rs __________________

Participant ID

11	What type of medical insurance do you have?<br><br>***[Yes=1; No=2]***	Free medical treatment<br>Commercial Insurance<br>None<br>Self-pay<br>Other<br>(_______________)<br>Specify	Free medical treatment<br>Commercial Insurance<br>None<br>Self-pay<br>Other<br>(_______________)<br>Specify	Free medical treatment<br>Commercial Insurance<br>None<br>Self-pay<br>Other<br>(___________)<br>Specify
12	Amount reimbursed from health insurance, if any?	Rs __________________	Rs __________________	Rs __________________
13	How do you pay for your hospitalisation costs?<br><br>***[Yes=1; No=2]***	Own saving<br>Family members paid<br>Employer paid<br>Borrowed from friends , relatives, employer<br>Borrowed from bank<br>Sold house, land, or other assets<br>Health insurance<br>Other<br>(Specify__________)	Own saving<br>Family members paid<br>Employer paid<br>Borrowed from friends , relatives, employer<br>Borrowed from bank<br>Sold house, land, or other assets<br>Health insurance<br>Other<br>(Specify__________)	Own saving<br>Family members paid<br>Employer paid<br>Borrowed from friends , relatives, employer<br>Borrowed from bank<br>Sold house, land, or other assets<br>Health insurance<br>Other<br>(Specify__________)
14	Proportion of money in percentage (%) did you spent from the above mentioned source for your hospitalisation?	Own savings____________%<br>Family members paid__________________%<br>Employer paid__________%<br>Borrowed from friends, relatives, employer ______%<br>Borrowed from bank _____%<br>Sold house, land, or other assets ________________%<br>Health insurance________%<br>Other ________________%<br>(Specify ___________)	Own savings____________%<br>Family members paid__________________%<br>Employer paid__________%<br>Borrowed from friends, relatives, employer ______%<br>Borrowed from bank _____%<br>Sold house, land, or other assets ________________%<br>Health insurance________%<br>Other ________________%<br>(Specify ___________)	Own savings___________%<br>Family members paid_________________%<br>Employer paid_________%<br>Borrowed from friends, relatives, employer _____%<br>Borrowed from bank ____%<br>Sold house, land, or other assets _______________%<br>Health insurance_______%<br>Other _______________%<br>(Specify ___________)

15. Time interview ended: __ __ : __ __
HR MIN

Participant ID

कार्स: सर्विलेंस स्टडी

साक्षात्कारकर्ता के लिए निर्देश: क्या प्रतिभागी ने सूचना सहमति प्रपत्र पर हस्ताक्षर कर दिए है? तब तक आगे न बढे जब तक सहमति पत्र पर हस्ताक्षर न हो जाए।

परिवार ID	प्रतिभागी ID
CEB कोड	सक्षात्कारकर्ता ID
सक्षात्कार की तिथि: DD/ MM/ YY	साक्षात्कार शुरू करने का समय [Hr:min]

SECTION – 1: DEMOGRAPHIC, SOCIO-ECONOMIC AND RESIDENTIAL DETAILS	
1.प्रतिभागी का नाम:<br>प्रथम नाम:<br>मध्य नाम:<br>उपनाम:	____<br>____<br>____
2.Father's/Spouse's name:<br>प्रथम नाम:<br>मध्य नाम:<br>उपनाम:	____<br>____<br>____
3.पता/विवरण<br>गली:<br>जिला:<br>राज्य:<br>पिन कोड:	____<br>____<br>____<br>____
5. टेलीफोन न.<br>निवास:<br>कार्यालय:<br>मोबाइल:	____<br>____<br>____
6. ई–मेल ID	(1)<br>(2)
7. जन्म स्थान:<br>जिला:<br>राज्य	____<br>____<br>____
8. आयु (पूर्ण वर्षो में)	
9. जल्न तिथि: (यदि उपलब्ध है)	DD / MM / YYYY

10. *लिंग*	*पुरूषः* 1<br>*स्त्रीः* 2<br>*लिंग परिवर्तन* 3	
11. *वैवाहिक स्थितिः*	*अविवाहित* 1<br>*विवाहित* 2<br>*विधवा/विधुर* 3<br>*अलग/तलाकशुदा* 4<br>*अन्य* 5	*यदि अन्य (विवरण दें)*<br>____________
12. *आपकी मात्र भाषा क्या हैं?*	*असामी* 1 *मलयालम* 10<br>*बालोची* 2 *मराठी* 11<br>*बंगाली* 3 *पंजाबी* 14<br>*गुजराती* 4 *सिंधी* 15<br>*हिन्दी* 5 *तेलगु* 16<br>*कन्नड* 7 *तामिल* 17<br>*कश्मीरी* 8 *उर्दू* 18<br>*मैथिली* 9 *अन्य* 19	*यदि अन्य (विवरण दें)*<br>____________
13. *आप किस धर्म को मानते हैं?* (Optional)	*हिन्दु* 1<br>*मुस्लिम* 2<br>*सिक्ख* 3<br>*ईसाई* 4<br>*जैन* 5<br>*बौध* 6<br>*कोई धर्म नहीं* 7<br>*अन्य (विवरण दें)* 8<br>*कोई उत्तर नहीं* 9	*यदि अन्य (विवरण दें)*<br>____________
14. *आप किसी जाति या जनजाति से सबंध रखते हैं?* (Optional)	*हॉ* 1<br>*नही* 2<br>*जानते नही* 3<br>*उत्तर नही देना चाहते* 4<br>*लागू नही* 5	
14.a. *यदि हॉ*<br>*आपकी जाति/जनजाति क्या है?*(Optional)	*अनुसूचित जाति* 1<br>*अनुसूचित जनजाति* 2<br>*अन्य पिछडे वर्ग* 3<br>*सर्वाधिक पिछडे वर्ग* 4<br>*अन्य* 5	*यदि अन्य (विवरण दें)*<br>____________
15. *आपने औपचारिक शिक्षा में कुल कितने वर्ष बिताए?**<br>* *कुल वर्ष जो प्रतिभागी ने किसी शिक्षा संस्थान में बिताए। (विद्यालय, कॉलिज, धार्मिक विद्यालय आदि)*		***वर्ष***

16. शिक्षा का स्तर (उच्चतम डिग्री प्राप्त)<br><br>* एक मनुष्य जो बिना किसी औपचारिक या निम्न शिक्षा के कोई भी भाषा समझ के साथ पढ़ और लिख सकता है।<br><br>** एक मनुष्य जो न पढ़ सकता है न ही लिख सकता हे या केवल पढ़ सकता है किंतु कोई भी भाषा लिख नही सकता।	Professional degree/post graduate 1<br>Graduate (B.A/B.Sc/B.Com/Diploma) 2<br>Secondary School / Intermediary (ITI course, class XII/X or Intermediate) 3<br>High school (class V to IX) 4<br>Primary School (upto Class IV) 5<br>*Literate, no formal education 6<br>**Illiterate 7<br>Others 8	यदि अन्य (विवरण दें)<br>________________<br>________________
17. आपकी व्यवसायिक स्थिति?	कर्मचारी 1<br>विद्यार्थी 2<br>गृहणी 3<br>सेवानिवृत 4<br>बेरोजगार 5	**"1" प्र॰ 17.a पर जाएं अन्यथा प्र॰ 8 पर जाएं**
17.a. यदि "कर्मचारी" हो तो आपका वर्तमान व्यवसाय क्या है?<br>**{नीचे दिए गए नजदीकी उपयुक्त व्यवसायिक कोड प्रयोग करें}**		
18. क्या आप पिछले 10 साल से दूसरे व्यवसाय में थे?	हाँ 1<br>नहीं 2	**"2" तो प्र॰. 19 पर जाएं**
18.a. यदि "हॉं" व्यवसाय का नाम?<br>**{नीचे दिए गए नजदीकी उपयुक्त व्यवसायिक कोड प्रयोग करें}**		

Coding list for employment (for Q.17.a and Q.18.a)- refer to annexure for definition of skilled, semi-skilled, un-skilled

प्रोफेशनल, बडा व्यापार, ज़मीदार, विश्वविद्यालय अध्यापकर, प्रथम श्रेणी के IAS वकील,	1
प्रशिक्षित क्लर्क, मध्यम व्यवसायिक, मध्यम स्तर के किसान, अध्यापकर, रखरखाव–प्रभारी, पर्सोनल मैनेजर,	2
कुशल श्रमिक, लधु व्यवसायिक, छोटे किसान,	3
अर्ध कुशल श्रमिक, हाशि, के किसान,	4
रिक्शा चालक, आर्मी जवान, बढ़ई, फिट्र, अकुशल श्रमिक, भूमिहीन किसान	5

19.आपके परिवार की प्रतिमाह क्या आय है?<br><br>***कृपया परिवार में को उन सभी सदस्यों की आय शामिल करे जो सदस्य परिवार में अपनी आय का योगदान देते हैं।***	<3000 1<br>3000-10,000 2<br>10,001-20,000 3<br>20,001-30,000 4<br>30,001-40,000 5<br>40,001-50,000 6<br>>50,000 7<br>*उत्तर नही देना चाहते* 8<br>*जानते नही* 9	
20.*क्या आपके पास खाना बनाने के लिए अलग से रसोई है?*	*हाँ* 1<br>*नहीं* 2	
21.*आप खाना बनाने के लिए कौन सा ईधंन प्रयोग करते हैं?*<br>***अगर आप एक से ज्यादा साधन प्रयोग करते हैं तब जो आप समान्यता जो साधन ज्यादा प्रयोग करते हैं वो लिखें।***	*कोयला/चारकोल का बना हुआ/ मिट्टी का तेल* 1<br>*बिजली/एल.पी.जी गैस/ सौर ऊर्जा/स.एन.जी (आई.जी.एल)* 2<br>*लकडी/गोबर* 3<br>*अन्य* 4	*अन्य, विवरण दें।*<br>________________
22.*घर मे प्रयोग होने वाले पिने के पानी का साधन क्या हैं?*<br>***अगर आप एक से ज्यादा साधन प्रयोग करते हैं तब जो आप समान्यता जो साधन ज्यादा प्रयोग करते हैं वो लिखें।***	*सार्वजनिक साधन* 1<br>*निज़ी साधन (सहभाजी)* 2<br>*निज़ी साधन (स्वयं का)* 3<br>*बोतल का पानी* 4<br>*नल का परिष्कृत पानी* 5<br>*अन्य* 6	*अन्य, विवरण दें।*<br><br>________________
23.*आप किस प्रकार का शौचालय सुविधा प्रयोग करते हैं?*	*सार्वजनिक शौचालय* 1<br>*सहभाजी शौचालय* 2<br>*निजी शौचालय* 3<br>*अन्य* 4	*अन्य, विवरण दें।*<br>________________
24.*निम्नलिखित में से आपका स्वयं का क्या है?*<br><br>***[हाँ =1; नहीं =2]***	a. *टेलीविज़न*<br>b. *रेफ्रिजरेटर*<br>c. *वाशिंग मशीन*<br>d. *माइक्रोवेव*<br>e. *ग्राइंडर*<br>f. *मोबाइल फोन*<br>g. *डी.वी.डी प्लेअर*<br>h. *कम्पयूटर*<br>i. *कार*<br>j. *मोटर साइकल/स्कूटर*	

Participant ID

	k. साइकल	
25. संभवतः क्या आप अपने वर्तमान निवास स्थान को एक या दो वर्ष छोडकर चले जाएंगे?	हाँ 1<br>नहीं 2<br>पता नहीं 3	
26. यदि आप अपने वर्तमान निवास स्थान को छोडकर चले जाते है तो हम किससे आपका नया पता और टेलीफोन न॰ प्राप्त कर सकते हैं?<br>***दो संपर्क चुनिए***	पड़ोसी 1<br>रिश्तेदार 2<br>दोस्त 3<br>नियोक्ता 4<br>किसी सक संपर्क 5<br>अन्य 6<br>विवरण दें ______________________________	1st<br>2nd
27. प्रथम संपर्क व्यक्ति का नाम<br>प्रथम नाम:<br>मध्य नाम:<br>उपनाम:		
28. प्रथम संपर्क व्यक्ति का पता		
29. प्रथम संपर्क व्यक्ति का फोन न॰ (निवास, कार्यालय, मोबाइल)	निवास ___________(एरिया कोड)______________(न॰)<br>कार्यालय ___________(एरिया कोड)______________(न॰)<br>मोबाइल ____________________________________(न॰)	
30. दूसरे संपर्क व्यक्ति का नाम<br>प्रथम नाम:<br>मध्य नाम:<br>उपनाम:		
31. दूसरे संपर्क व्यक्ति का पता		
32. दूसरे संपर्क व्यक्ति का फोन न॰ (निवास, कार्यालय, मोबाइल)	निवास ___________(एरिया कोड)______________(न॰)<br>कार्यालय ___________(एरिया कोड)______________(न॰)<br>मोबाइल ____________________________________(न॰)	

भाग– 2: तंबाकू और शराब का उपभोग, खान–पान संबधी आदते, शारीरिक क्रियाशीलता और नींद।		
**PART – A: तंबाकू का प्रयोग**		
1. क्या आपने कभी किसी भी रूप मे तंबाकू प्रयोग किया है?	हाँ 1<br>नहीं 2	**"2" go to Q. 8**
2. *आप किस रूप मे तंबाकू का सेवन करते है?*<br>***[हाँ=1; नहीं =2]***	a. *धूम्रपान के रूप में*<br>b. *चबाने के रूप में*<br>c. *किसी ओर रूप में (सुघंने, टूथपेस्ट, सुंघना आदि)*	
3. *क्या आप अभी भी तंबाकू का सेवन कर रहे है?*<br>* *पिछले 6 महीनों से*	हाँ 1<br>नहीं 2	**"2" go to Q. 5**
4. *यदि हाँ प्रायः कितनी बार?*<br>***[निरंतर (≥ हफ्ते मे एक बार से ज़्यादा) – 1; कभी–कभी; (हफ्ते मे एक बार से कम) – 2; न॰–3 ; नहीं – 9]***	*धूम्रपान के रूप में* *चबाने के रूप में*	*किसी ओर रूप में*

5. *मात्रा और प्रयोग की अवधि*

तंबाकू प्रयोग करने के प्रकार	*ब्रांड नाम*	*प्रयोग करने की अवधि*		*महिने में प्रयोग करने की मात्रा*<br>**कितना धूम्रपान किया*<br>*** *कितनी बार धूम्रपान किया*<br>**** *लगभग कितनी मात्रा (ग्राम में)*	*यदि आपने निम्नलिखित पदार्थो का सेवन बंद बंद कर दिया है (समय में) महिने/वर्ष जब से सेवन बंद किया है।*	
		वर्ष	*महिने*		*वर्ष*	*महिने*
1. *सिगरेट**						
2. *बीड़ी**						
3. *सिगार**						
4. *हुक्का *****						
5. *चबाने वाला तम्बाकू ******						
6. *पान ज़र्दे के साथ******						
7. *पान मसाला ज़र्दे के साथ ******						
8. *नसवार***						
9. *गुटखा****						
10. *अन्यः*<br>*विवरण दें* ________						

6. *जब आपने पहली बार रोज़ाना धूम्रपान करना शुरू किया था तब आपकी उम्र क्या थी?* **[Not applicable – write '99' in the box]**	*वर्ष*	
7. *जब आपने पहली बार रोज़ाना धूम्रपान रहित पदार्थो का सेवन शुरू किया था तब आपकी उम्र क्या थी?* **[Not applicable – write '99' in the box]**	*वर्ष*	
8. *क्या आप अन्य व्यक्तियों के द्वारा तम्बाकू धूम्रपान प्रयोग करने से रोज़ाना घर या कार्य क्षेत्र में प्रभावित होते है*?* ** कम से कम हफ्ते में एक दिन।*	*हाँ* 1<br>*नहीं* 2	**"2" go to PART B**
9. *यदि हाँ*<br>*हफ्ते में कितने दिन*?*<br>*दिन के दौरान कितना समय*?*	:<br>*घंटे* *मिनट*	

PART – B: शराब (मदिरा) का सेवन

1. क्या आपने कभी शराब का सेवन किया है?	*हाँ* 1<br>*नहीं* 2	**"2" PART C पर जाएं**
2. आप शराब का सेवन कितनी बार करते है?<br><br>***कभी–कभी हफ्ते में एक बार**	वर्तमान समय में रोज़ाना शराब का सेवन करते हैं 1<br>वर्तमान समय में कभी–कभी (अवसर पर) शराब का सेवन करते हैं * 2<br>पहले शराब का सेवन करते थे (6 महीने से ज़्यादा समय पहले) 3<br>हाल ही में शराब का सेवन बंद कर दिया (6 महीने से कम समय पहले) 4<br>शराब का सेवन कभी नहीं किया 5	**"5" PART C पर जाएं**

3. **शराब का सेवन करने वालों का वर्तमान व भूतपूर्व विवरण**

प्रयोग की गई शराब के प्रकार	*प्रयोग करने की अवधि*		*आवृती प्रति सप्ताह*	*मात्र*** *ml*/पैग में (अवसर में)	*यदि बंद कर दिया तो कितने समय से)वर्षो में*	
	वर्ष	*महिने*			*वर्ष*	*महिने*
a) स्थानीय शराब जैसे–देसी, अरक, टोडी आदि						
b) शराब जैसे– विहस्की, रम, बराण्डी, जिन, वोडका						
c) बीयर						
d) वाईन						

** रूपांतरण

1 small peg = 30 ml; 1 large peg = 60 ml; 1 extra large peg = 90 ml

1 glass of beer = approx. 325 ml

1 glass of wine = 100 ml

कृपया कुल खपत जानने के लिए साधारण नाप तोल का साहारा ले! (प्रत्येक अवसर पर मि.ली. में)

PART – C: *शारिरिक क्रियाशीलता (संक्षिप्त अंर्तराष्ट्रीय शारिरिक क्रियाशीलत प्रश्नोत्तर)*

हम उन विभिन्न क्रियाओं को जानने के इच्छुक हैं जिन्हें लोग अपनी रोज़–मर्रा की जिदंगी मे अपने काम में शामिल करते हैं। मैं आपसे एक हफ्ते में बिताए गए ऐसे समय के बारे में पूछने जा रहा/रही हूँ जिसमें आप सामान्य रूप से क्रियाशील हों। यदि आप अपने आपको क्रियाशील मनुश्य नहीं मानते तो कृपया इन प्रश्नों का उत्तर दें। ऐसी क्रियाओं पर विचार करें जिन्हें आप अपने कार्य क्षेत्र, एक स्थान से दूसरे स्थान पर आने–जाने और खाली समय में मनोरंजन या शरीर को चुस्त रखने के लिए करते हैं।

अब आप पिछले 7 दिनों में अपने काम के दौरान की गई प्रबल मेहनत वाली शारिरिक क्रिया के बारे मे विचार करेंगे। प्रबल शारिरिक क्रिया का मतलब यह है कि जिसमें श्वास सामान्य कि अपेक्षा अधिक तेज़ हो जाती है जैसे कि भारी वज़न उठाना, खुदाई करना, ऐअरोबिक्स या तेज़ साईकल चलाना। केवल उन्हीं शारिरिक क्रियाओं के बारे में विचार करें जो कम से कम 10 मिनट लगातार की हो।

1. *सामान्य 7 दिनों में आपने कितने दिन प्रबल मेहनत वाली शारिरिक क्रिया की है?*	दिन प्रति सप्ताह =1<br>मना करना (Q.3 पर जाएं) = 2<br>पता नहीं/पक्का पता नहीं (3 पर जाएं) = 3<br>कोई शारिरिक क्रिया नहीं करते= 4	
2. *जिन दिनों आप भारी शारिरिक क्रिया करते है उन दिनों मे से एक सामान्य दिन में आप कितना लगातार 10 मिनट भारी शारीरिक क्रिया करते हैं?*	घंटे/मिनट प्रति दिन = 1<br>मना करना (Q.2a पर जाएं) = 2<br>पता नहीं/पक्का पता नहीं (2a पर जाएं)=3	HR : MIN
2a. *साक्षात्कारकर्त्ता की जांचः यदि प्रतिवादी अपने बिताए गए समय के दिन–प्रतिदिन परिवर्तित होने के कारण उत्तर नहीं दे सकता है, कहिए, "मै आपके द्वारा उन दिनो मे से एक सामान्य दिन में से प्रबल मेहनत वाली शारिरिक क्रिया को जानने का इच्छुक हूँ जो आप औसतन करते है। क्या आाप मुझे यह बता सकते हैं कि आपने सामान्य 7 दिनों में कुल कितना समय प्रबल शारिरिक क्रिया की है"?*	घंटे/मिनट प्रति दिन = 1<br>मना करना = 2<br>पता नहीं/पक्का पता नहीं = 3	HR : MIN

अब आप मध्यम मेहनत वाली शारिरिक क्रियाओं के बारे में विचार किजिए जो आपने पिछले सामान्य 7 दिनों में की है। मध्यम मेहनत वाली शारिरिक क्रिया आपकी श्वास की गति को सामान्य कि अपेक्षा कुछ–कुछ तेज कर देती है। इन क्रियाओं में चलने वाली क्रियाओं को शामिल नहीं किजिए फिर से केवल उन ही क्रियाओं को शामिल नहीं किजिए फिर से केवल उन ही 10 मिनट तक करते है।

3. *सामान्य 7 दिनों के दौरान आपने कितने दिन कम से कम 10 मिनट लगातार मध्यत मेहनत वाली शारिरिक क्रिया की है?*<br>*जैसे : बोझ उठाना, धीरे–धीरे साईकल चलान, टेनिस, बैडमिंटन, क्रिकेट, हाथों से कपड़े धोना, झाडू मारना, बागबानी करना, अपने 3 साल से कम उम्र के बच्चे की देखभाल करना, कार, मोटर कार और स्कूटर की सफाई करना, बज़ार से सब्ज़ियाँ और किराने का सामान पैदल घर तक लाना, सीढ़ियाँ चढ़ना (3 फ्लोर या ज़्यादा) पत्थर पर चटनी पीसना*	घंटे/मिनट प्रति दिन = 1<br>मना करना (Q.5 पर जाएं) = 2<br>पता नहीं/पक्का पता नहीं (5 पर जाएं) = 3	

4. *जिन दिनों आप मध्यम मेहनत वाली शारिरिक क्रिया करते है उन दिनों मे से एक सामान्य दिन में आप कितने समय लगातार 10 मिनट तक मध्यम मेहनत वाली शारिरिक क्रिया करते हैं?*	घंटे/मिनट प्रति दिन = 1<br>मना करना (Q.4a पर जाएं) = 2<br>पता नहीं/पक्का पता नहीं (4a पर जाएं) = 3	HR : MIN
4a. *साक्षात्कारकर्त्ता की जांचः यदि प्रतिवादी अपने बिताए गए समय के दिन–प्रतिदिन परिवर्तित होने के कारण या अलग–अलग कार्यों में व्यस्त होने के कारण उत्तर नहीं दे सकता है , कहिए, "मै आपके द्वारा उन दिनो मे से एक सामान्य दिन में से मध्यम मेहनत वाली शारिरिक क्रिया को जानने का इच्छुक हूँ जो आप औसतन करते है। क्या आाप मुझे यह बता सकते हैं कि आपने सामान्य 7 दिनों में कुल कितना समय मध्यम शारिरिक क्रिया की है"?*	घंटे/मिनट प्रति दिन = 1<br>मना करना = 2<br>पता नहीं/पक्का पता नहीं = 3	HR : MIN
अब आप उस समय के बारे में विचार करें किजिए जो आपने पिछले सामान्य 7 दिनों में चलने में बिताया है। इसमें कार्य स्थल से घर तक आना–जाना, यात्रा के लिए आना–जाना शामिल है। इसमें किसी भी तरह चलने वाली क्रिया शामिल है जिसे आप अकेले घूमकर, खेलकर, व्ययाम या फुर्सत में करते हैं जैसेः कम से कम लगातार 10 मिनट तक बस स्टाप, कार्य स्थल और बाज़ार चलकर जाते हैं।		
5. *सामान्य 7 दिनों के दौरान आप कितने दिन कम से कम 10 मिनट तक चले हैं?*	*दिन प्रति सप्ताह* =1<br>मना करना (Q.7 पर जाएं) = 2<br>पता नहीं/पक्का पता नहीं (Q.7 पर जाएं) = 3	
6. *जिन दिनों आप सामान्य चलते हैं उन दिनों में से एक सामान्य दिन में आप कितना समय चलने में बिताते हैं?*	घंटे/मिनट प्रति दिन (Q.7 पर जाएं) =1<br>मना करना (Q.6a पर जाएं) = 2<br>पता नहीं/पक्का पता नहीं (6a पर जाएं) = 3	HR : MIN
6a. *साक्षात्कारकर्त्ता की जांचः यदि प्रतिवादी अपने बिताए गए समय के दिन–प्रतिदिन परिवर्तित होने के कारण उत्तर नहीं दे सकता है, कहिए, "मै आपके द्वारा उन दिनो मे से एक सामान्य दिन में से चलने वाली क्रिया को जानने का इच्छुक हूँ जो आप औसतन करते है। क्या आाप मुझे यह बता सकते हैं कि आपने सामान्य 7 दिनों में कुल कितना समय चलने वाली क्रिया की है?"*	घंटे/मिनट प्रति दिन = 1<br>मना करना = 2<br>पता नहीं/पक्का पता नहीं = 3	HR : MIN
अब आप पिछले सप्ताह 7 दिनों के दौरान बैठकर बिताए गए समय के बारे में बताएं। इसमे आप कार्य स्थल पर, घर पर, पाठ्यक्रम और खाली समय में बैठने वाले समय को भी शामिल कर सकते है। इसमें डे चलने में बिताया है। इसमें कार्य स्थल से घर तक आना–जाना, यात्रा के लिए आना–जाना शामिल है। इसमें किसी भी तरह चलने वाली क्रिया शामिल है जिसे आप अकेले घूमकर, खेलकर, व्ययाम या फुर्सत में करते हैं जैसेः कम से कम लगातार 10 मिनट तक बस स्टाप, कार्य स्थल और बाज़ार चलकर जाते हैं।		
7. *सामान्य 7 दिनों के दौरान किसी एक दिन आमतौर पर आप कितना समय बैठकर बिताते हैं? इसमें लेटे हुए (जागे रहना) और इसी तरह बैठे हुए समय बिताए को भी शामिल करें। (*रात को सोने को शामिल न करें)*	*घंटे/मिनट प्रति कार्य दिवस {प्र. 8 (1) पर जाएं}*<br>*मना करने पर (प्र. 7 a पर जाएं)=2*<br>*नहीं जानते/पक्का नहीं (प्र. 7 a पर जाएं)=3*	HR : MIN

7a. साक्षात्कारकर्त्ता की जांचः यदि प्रतिवादी अपने बिताए गए समय के दिन–प्रतिदिन परिवर्तित होने के कारण उत्तर नहीं दे सकता है, कहिए, "मै आपके द्वारा उन दिनो मे से एक सामान्य दिन में से बैठे हुए बिताए गए समय को जानने का इच्छुक हूँ जो आप औसतन करते है। क्या आप मुझे यह बता सकते हैं कि आपने सामान्य 7 दिनों में कुल कितना समय बैठे हुए बिताया है?"	घंटे/मिनट (बुधवार में)<br>मना करना=2<br>नहीं जानते/पक्का नहीं=3	HR : MIN
8. Additional comments		

PART – D: सोए हुए ह्रदय का चिकित्सीय अध्ययन

1. सामान्यतः आप रात को कितने घंटे सोते है?<br>**आपकी रात को सोने की मुख्य समयावधि?**	कार्य दिवस पर<br>No. of hrs	सप्ताहांत पर<br>No. of hrs
2. एक सामान्य हफ्ते के दौरान आप 5 मिनट के लिए या उससे ज्यादा कितनी बार ऊंघते है? ("00" लिखे यदि आप एक भी बार नहीं ऊंघते)	No. of times	

3. कार्यदिवस, सप्ताहांत (सप्ताह का अंतिम दिन), कितने घंटे निम्नलिखित में से प्रत्येक के लिए प्रायः आपने कितना अनुभव किया है। कृपया संकेत करें। **(नीचे दिए गए कोड से उल्लेख करें)**

[कभी नहीं=1; कभी–कभार (1/महीना या कम)=2; कभी– कभी (2-4/महीनें)=3; प्रायः (5-15/महीनें)=4; अधिकतर हमेशा (16-30/महीनें)=5]

A. क्या सोते हुए परेशानी होती है

B. रात्री के दौरान जाग लाना और फिर से नींद आने में परेशानी होना

C. सुबह–सुबह जाग जाना और फिर से सोने में असमर्थ होना।

D. दिन के दौरान अशांती (बैचानी) महसूस करना इसकी परवाह किये बिना कि आपने कितनी नींद ली है।

E. प्रयाप्त रूप से नींद नहीं लेंते।

F. सोने के लिए नींद की गोली या अन्य कोई दवाई लेते है।

प्र. 6–10 नींद के दौरान खर्राटे लेने और श्वास लेने के विषय मे है। इन प्रश्नो का उत्तर देते समय कृपया गोर किजिए कि आप अपने बारे मे क्या जानते है।

4. क्या आपने कभी खर्राटे लिए हैं? (आजकल या पहले किसी समय)?	हाँ<br>नहीं<br>पता नहीं	1<br>2<br>8	**"2", "8" go to Q.7**
5. प्रायः आप कितना खर्राटे लेते हैं?	अब खर्राटे नहीं लेते<br>कभी–कभार (<1 रात/सप्ताह)<br>कभी– कभी (1-2 रातें/सप्ताह)<br>बार–बार (3-5 रातें/सप्ताह)<br>हमेशा या अधिकतर (6-7 राते/सप्ताह)<br>पता नहीं	0<br>1<br>2<br>3<br>4<br>8	**"0" go to Q.7**

6. आप कितनी ज़ोर से खर्राटे लेते हैं?	ज़ोर से ज़्यादा सांस लेने की अपेक्षा थोड़ा ज़्यादा खर्राटे लेते हैं? 1<br>लगभग इतनी आवाज़ से जितना बड़बड़ाते या बातें करते हैं? 2<br>बाते करने की अपेक्षा ज़्यादा जोर से 3<br>इतनी ज़ोर से लेते है कि बंद दरवाज़े के बाहर सुनाई देता है 4<br>जानते नहीं 8	
7. क्या आपने कभी ध्यान दिया है या घर वालों ने आपको बताया है कि सोने के दौरान आप किसी समय सांस लेना बंद करते हैं?	हाँ 1<br>नहीं 2<br>पता नहीं 8	**"2", "8" go to Q.9**
8. प्रायः कितने अंतराल मे आप सोने के दौरान आप किसी समय सांस लेना बंद करते हैं?	कभी–कभार (<1 रात/सप्ताह) 1<br>कभी– कभी (1-2 रातें/सप्ताह) 2<br>बार–बार (3-5 रातें/सप्ताह) 3<br>हमेशा या अधिकतर (6-7 राते/सप्ताह) 4<br>पता नहीं 8	
9. क्या आपको डाक्टर के द्वारा बताया गया है कि आपको sleep aroma है? (एसी अवस्था जिसमें सोने के दौरान समय समस पर सांस बंद होती रहती है)	हाँ 1<br>नहीं 2<br>पता नहीं 8	**"1" go to Q.11**<br>**"2", "8" go to Q.10**
10. क्या आपको कभी डाक्टर के द्वारा यह बताया गया है कि आपको कोई नींद संबंधी रोग (बीमारी) है।	हाँ 1<br>नहीं 2<br>पता नहीं 8	**"2", "8" go to Q.11**
10.A. यदि ऊपर दिए गए प्रश्न का उत्तर हाँ है तो कृपया बीमारी का विवरण दें।<br>____<br>____		
11. क्या आप समान्यतः सोने के दौरान आक्सीजन थैरपी (मास्क या नासिका नली के द्वारा आक्सीजन पहँचाना) प्रयोग करते हैं?	हाँ 1<br>नहीं 2	
12. पिछले साल के दौरान जहाँ आप सोते है आपके साथ या आपके कमरे के पास परिवार के ण्क या एक से अधिक सदस्य सोएं हैं?	कभी नहीं 1<br>कभी–कभी 2<br>कभी–कभार 3	

13. निम्नलिखित परिस्थितियों में से प्रत्येक परिस्थिति मे आप कितना ऊंघे हो या नींद महसूस कि है? (नीचे दिए काड से संकेत करे)। यदि
[No chance=1; Slight chance=2; Moderate chance=3; High chance=4]
आप ऐसी परिस्थितियों में कभी नहीं या कभी–कभार आए हैं तो कृप्या उतम अनुमान लगाएं।

A. बैठे हुए या पढ़ते हुए
B. टी.वी. देखते हुए
C. सार्वजनिक जगह पर निष्क्रिय बैठे हुए
D. यात्री के रूप में कार में लगातार एक घंटे सफर करते हुए
E. परिस्थिति अनुसार दोपहर बाद आराम करते हुए
F. बैठकर किसी से बात करते हुए
G. दोपहर खाने बाद बैठे हुए
H. कार के अंदर जब आप कुछ देर के लिए ट्रैफिक मे रूके हुए।
I. रात के खाने की मेज पर
J. गाड़ी चलाते समय

14. क्या आप प्रायः ऐझपरिन युक्त औषधि जैसे–बफरीन, ऐस्परिन या ऐस्ककरपरटीन लेते हैं?	कभी नहीं	1	
	प्रायः हफ्ते में एक बार से कम	2	
	हफ्ते में एक या दो बार	3	
	प्रत्येक दूसरे दिन (दो में से एक दिन)	4	
	प्रतिदिन	5	
	नहीं जानते	8	
15. क्या आप ड्राइव (चालन) करते हैं?	हाँ	1	
	नहीं	2	

16. यदि आपका ऊपर दिए गए प्रश्न का उत्तर हाँ है तो निम्नलिखित प्रश्नों का उत्तर दिजिए अन्यथा पार्ट **E** डाईट पर जाएं।

A. आपने कितने वर्ष ड्राइविंग की है?
B. अक्सर आप ड्राइव करते है?
[everyday=1; sometimes=2; rarely/never=3]
C. जब से आपने ड्राइव शुरू की है आप कितनी दुर्घटना कर चुके है जब आप स्वयं चालक थे?
D. आपने गत वर्ष मे कितनी दुर्घटनाए की है जब आप स्वयं चालक थे?

PART – E: आहार (खुराक)

1. क्या आप कोई विशेष आहार (खुराक) ले रहे है?	हाँ	1	
	नहीं	2	**"2" go to Q.4**

2. यदि हाँ तो वर्तमान मे क्या आहार (खुराक) ले रहें है?<br>**[हाँ = 1; नहीं = 2]**	मधुमेह संबधी आहार<br>कम वसा युक्त आहार<br>अतिरिक्त रेशे युक्त आहार<br>कम नमक युक्त आहार<br>वज़न कम करने वाला आहार<br>अन्य (विवरण दें)<br>________________________	
3. कितने वर्षों से आप विशेष आहार ले रहें है?	**वर्ष** **महिने**	
4. आप फिर से पकाए हुए तेल का कितना प्रयोग करते हैं	रोज़ाना 1<br>हर दूसरे दिन (दो मे से एक दिन) 2<br>हफ्ते मे एक या दो बार 3<br>हफ्ते मे एक बार से भी कम 4<br>कभी नहीं 5	

5. आप निम्नलिखित आहार कितनी बार लेते हैं? **[आहार की आवृति उपयुक्त कॉलम में लिखिए]**

**क्र॰ सं॰**	**आहार समूह**	**आहार बिल्कुल भी नहीं/ एक बार से भी कम/ महीने में[√]**	**उपभोग प्रतिमास**	**उपभोग प्रतिमाह**	**उपभोग प्रतिदिन**
1	मांस				
2	मुर्गी				
3	जार्गन मांस				
4	मछली				
5	सीपदार मछली और केकंडा				
6	अंडे				
7	दूध और दूघ के पदार्थ				
8	दूध आधारित मिठाई				
9	ज़्यादा तले पदार्थ– वेर्स्टन स्टाईल				
10	ज़्यादा तले पदार्थ– देसी स्टाईल				
11	वेर्स्टन स्टाईल – मिठाई/नैक्स				
12	मिठाई				
13	ठंडी बीयर				
14	फल (1)				
15	फल (2)				
16	फलों का जूस				
17	बदाम/बीज				
18	हरी पतेदार सब्ज़ियां				
19	अन्य कच्ची सब्जियां				

20	छाल और फलियां				
21	आपार और मुरब्बा				
22	अन्य पकी हुई सब्जियां				
23	परिष्कृत अनाज कम रेशे के साथ				
24	पूर्ण अनाज				
25	चय का अपभोग				
26	कॉफी का अपभोग				

खाद्य पदार्थों का समूह (प्रत्येक समूह की प्रदर्शित मद)
मांस/मुर्गी– मुर्गा, भेड का मांस, बछडे का मांस, खरगोश का मांस, टर्की का मांस, बमख का मांस, तीतर का मांस, इन सभी की कढी, गाँय और भैंस का मांस, सुअर का मांस
आर्गन मांस– कलेजा, गुर्दा, तिल्ली, सांसज (मांसादि से भरी हुई ख्ज्ञ्ज्ञ्य वस्तु),पांवे। मछली– नदी, और समुंद्र की मछली, परिरक्षित (प्रिर्जव) मछली जैसे – नमक युक्त मछली, टिन के डब्बे में सुरक्षित रखी हुई मछली, सूखी मछली
सीपदार मछली और केंकडा– केंकडा, समुद्रफेंनी, झींगा, घेगा, बडी मछली
अंडे–प्रतिारक्षित अंडे, बतख के अंडे
दूध और दूध के पदार्थ –दूध, दूध से जमाकर बनाया गया खाद्य पदार्थ पनीर, दही, रायता, लस्सी,
दूध आधारित मिठाई –––– कस्डर्ड (पकाया हुआ दूध) खोया, फिर्नी, खीर, दूध की बनी खीर।
ज्यादा तले हुए पदार्थ –––– वेर्स्टन स्टाईल –– चिप्स, आलु की चिप्स, प्याज के छल्ले, चिकन बडी।
ज़्यादा तले हुए पदार्थ ––– देशी स्टाईल –––– समोसे, पापड़, पकोटे, सेब, मतकपारे, ऐग रोल, पूरी, कचौडी।
वेर्स्टन स्टाईल मिठाई, स्नैकस ––– केक, पाई, चॉकलेट, टॉफी, बिस्कुट।
मिठाई ––– बर्फी, लड्डू, रसगुल्ले, गुलाब जामुन, हलवा, सबईया, मोहालबीया।
ठंडे पेय पदार्थ ––– कार्बोनेटड पेय पदार्थ, शरबत और अन्य सॉफट पेय पदार्थ (अल्कोहल रहित पेय पदार्थ)
फल (1) ––– स्ट्राबेरी, अन्नास, जामुन, सेब
फल (2) ––– सभी मौसमी फल उपरोक्त फलो को छोड़कर।
फलों का रस ––– किसी भी किस्म का रस, घर में बना रस, खरीदा हुआ रस, ताजा रस, ठंडा रस।
बादाम/बीज़ ––– मूँगफली, बादाम, सूरजमुखी के बीज़, काजू, अखरोट।
हरी पत्तेदार सब्जी –––– सभी ताजी हरी पत्तेदार सब्जियाँ, पालक (साग), सरसों या शलगम, कच्चा या पका साग।
अन्य कच्ची सब्जियाँ ––– कोई भी कच्ची सब्जियाँ इसमें पिछली श्रेणी की सब्जियाँ शामिल नही है।
फलिया और दालें ––– इनमें सभी प्रकार की दालें, छोले और मसूर की दाल शामिल है।
आचार और मुरब्बा ––– अचार, चटनी, सब्जियों का मुरब्बा आदि।
अन्य पकी हुई सब्जियाँ ––– कोई भी पकी हुई सब्जियाँ इनमें पिछली श्रेणी की सब्जियाँ शामिल नही है।
परिष्कृत (शुद्ध) अनाज कम रेशो के साथ ––– उबले हुए चावल, तले हुए चावल, बिरयानी, पुलाव, इडली, डोसा, सूजी, साबूदाना, जौ, पास्ता, शीरमल नान, तफवाल, ब्रैड स्लाइस।
पुर्ण अनाज (ज़्यादा रेशे वाला खाना) ––– मोटे आटे की बनी रोटी, भरी मठरी, पूर्ण गेहूँ का दलिया, ब्रेड स्लाठस, पूर्ण भोजन।
चाय और कॉफी ––– काली चाय, कॉफी डूध और चीनी रहित और रहित और अन्य चाय।

SECTION – 3: स्त्री प्रजन्न संबंधी विवरण

यह भाग केवल महिला प्रतिभागी ही भरे, पुरूष प्रतिभागी यह भाग छोड़ दें और भाग 4 पर जाएं

1. आप कितनी बार गर्भवती हो चुकी है?<br>लागू नहीं=99	

Participant ID

2. जब आपको मासिक धर्म या महीना आना शुरू हुआ था, मब आपकी उम्र क्या थी?	Years			
3. क्या आपको मासिक धर्म/महीना होता है?	हाँ 1<br>नहीं 2	**"1" go to Q. 6**		
4. यदि नहीं तो कारण क्या है?	गर्भावस्था 1<br>बच्चा दूध पिता है 2<br>प्राकृतिक रजोनिवृति 3<br>शहयक रजोनिवृति 4<br>अन्य (विवरण दें) 5	***अन्य, विवरण दें।*** ________		
5. यदि आपको मासिक धर्म/महीना आना बंद हो चुका है, तो कितने समय से बंद है?	वर्ष	महिने		
6. क्या आप हारमोनल ड्रग्स या गर्भनिरोधक दवाएँ इस्तेमाल करती हैं?<br>***[हाँ = 1; नहीं = 2]***	पहले करती थी		यदि हाँ अवधि वर्षो में महीनों में<br>वर्ष महीने	
	आजकल करती हैं		यदि हाँ अवधि वर्षो में महीनों में<br>वर्ष महीने	

SECTION – 4: जीवन का स्वरूप (EQ-5D) © 1990 EuroQol Group. EQ-5D™ is a trade mark of the EuroQol Group.

बाक्स में दिए गए विकल्पों में से एक विकल्प की नियुक्ति कर कृपया उस कथन की और संकेत करे जो आपके आज के दिन की स्वास्थ्य की स्थिति का भली प्रकार वर्णन करता है।

1. गतिशीलता (चलना फिरना)	मुझे इधर–उधर घुमने में कोई समस्या नहीं है। =1<br>मुझे इधर–उधर घुमने में कोई समस्या है। =2<br>मै बिस्तर तक सीमित हूँ। =3	
2. स्वयं देखभाल	मुझे स्वयं की देखभाल करने में कोई समस्या नहीं है। =1<br>मुझे स्वयं को साफ रखने या तैयार करने में समस्या है। =2<br>मैं स्वयं को साफ रखने या तैयार करने में असक्षम हूँ? =3	
3. सामान्य क्रियाशीलता जैसे–कार्य करना, पढ़ाई करना, गृहकार्य, परिवारिक या फुरसत से की हुई क्रियाएँ	मुझे सामान्य क्रियाएँ करने में समस्या नहीं होती। =1<br>मुझे सामान्य क्रियाएँ करने में कुछ समस्या होती हैं। =2<br>मैं अपनी सामान्य क्रियाएँ करने में असक्षम हूँ? =3	
4. दर्द/ असुविधा	मुझे दर्द या असुविधा नहीं है।=1<br>मुझे मध्यमवर्ती दर्द या असुविधा है। =2<br>मुझे अत्याधिक दर्द या असुविधा है। =3	
5. दुश्चिंता उत्कंठा/दवाब	मैं उत्कंठा या दवाब में नहीं हूँ =1<br>मैं मध्यमवर्ती उत्कंठा या दवाब में हूँ =2	

	मैं अत्याधिक उत्कंठा या दवाब में हूँ =3	

To help people say how good or bad their state of health is, we have drawn a scale (rather like a thermometer) on which the best state you can imagine is marked 100 and the worst state you can imagine is marked 0.

I would like you to indicate on this scale how good or bad your own health is today, in your opinion. Please do this by drawing a line from the box below to whichever point on the scale indicates how good or bad your state of health is today.

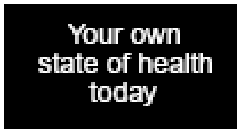

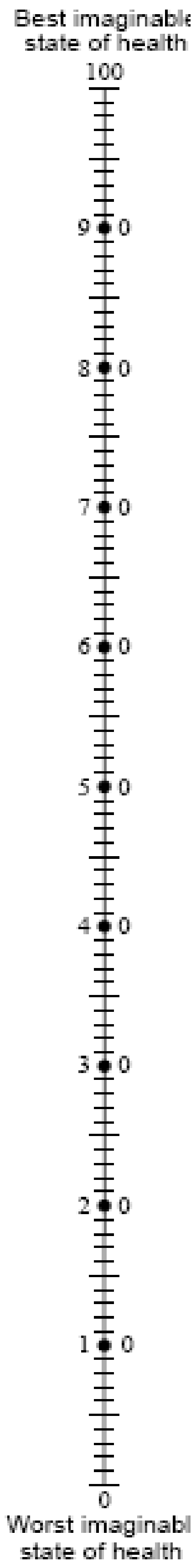

Participant ID

SECTION – 5: चिकित्सीय (मेडिकल) विवरण

PART-A: कार्डियोमेटाबॉलिक डिज़ीज़ और सनके जोखिक वाले कारक

1. क्या आपको डाक्टर के द्वारा बताया गया है की आपको निम्नलिखित मे से कोई बीमारी है?<br><br>**[हाँ =1; नहीं =2; पता नहीं=3]**	उच्चरक्तचाप<br>मधुमेह<br>उच्च कोलेस्ट्राल<br>हृदय रोग<br>स्ट्रोक या घात (लकवे का अटैक)<br>किड़नी डिज़ीज	

*गर्भावस्था को छोड़कर गर्भावस्था के दौरान उच्चरक्तचाप और मधुमेह से प्रेरित होना।

यदि प्र.1 के विकल्पों में से एक भी उत्तर हाँ मे है तो भाग- B पर जाइए अन्यथा पूरे भाग B को छोड़कर भाग C पर जाइए

PART - B: बीमारी के विष्शिट (विशेष) प्रश्न

I. उच्चरक्तचाप

यह भाग भरिए यदि भाग A (प्र.1) उच्चरक्तचाप का उत्तर हाँ मे है।

a. आपको कितने वर्षों से उच्चरक्तचाप है?		अवधि वर्षो में महीनों में<br>**वर्ष** **महिने**
b. वर्तमान में आप इसका क्या इलाज ले रहे हैं?<br>**[हाँ=1; नहीं=2]**<br>***पौराणिक औषधि, थैरपी के साथ योगा, आर्युवेद, यूनानी, होमियोपैथिक चीकित्सा, तिब्बतन, प्रकृतिक चीकित्सा, मेडिटेशन्**	निर्धारित खान–पान (आहार) संबंधी सुधार<br>निर्धारित शारीरिक व्यायाम<br>पौराणिक औषधि/ थैरपी*<br>एलोपैथीक ड्रग्स (इंगलिश/माफडर्न)<br>कोई नहीं	
c. आप कितना नियमानुसार दवाई लेते हैं?	नियमित रूप से 1<br>अक्सर भूल जाते हैं 2<br>जब उच्चरक्तचाप महसूस होता है तब ही दवाई लेते हैं 3<br>एक समय में एक महीने से ज़्यादा अनियमित हो जाते है 4<br>कभी भी दवाई नहीं लेते 5	

यदि आपके उपरोक्त प्रश्न का उत्तर "4" है तो अगले प्रश्न पर जाएं अन्यथा प्र. e पर जाएं।

d. अनियमितता का कारण क्या है?	खर्च वहन नहीं कर पाते 1<br>सहन नहीं कर पाते 2<br>स्वस्थ हो गए हैं 3<br>कोई कारण नहीं है 4<br>याद नहीं 5<br>अन्य (विवरण दें) 6	**अन्य, विवरण दें।**<br>________________

e. क्या आपको लगता है कि आपका रक्तचाप अच्छे नियंत्रण में है?	हाँ 1<br>नहीं 2<br>पता नहीं 3	
f. क्या आपको डाक्टर कहता है कि आपका रक्तचाप अच्छे नियंत्रण में है?	हाँ 1<br>नहीं 2<br>पता नहीं 3	
g. आपके पिछले रक्तचाप की रिकॉर्डिंग क्या थी? (जब आपकी डाक्टर ने जांच की थी)	____________(systolic) / _____________(diastolic) mmHg	
h. आपने अपने डाक्टर से आखिरी सलाह कब ली थी?	एक महीने से कम 1<br>एक महीने से ज़्यादा 2<br>3 महीने से ज़्यादा 3<br>6 महीने से कम 4<br>6 महीने से ज़्यादा 5	
i. क्या आपके पास उच्चरक्तचाप संबधी चिकित्सीय विवरण या औषध विधि है?	हाँ 1<br>नहीं 2<br>पता नहीं 3	
j. यदि उत्तर हाँ है तो प्रतिभागी से चिकित्सीय विवरण दिखाने को कहिए और नीचे रोग निदान लिखिए?		
k. अत्यंत हाल ही में चिकित्सीय विवरण या चिकित्सा से परतापित रक्तचाप पर संक्षिप्त लेख लिखिए?		
**II. मधुमेह**<br>**यह भाग भरिए यदि भाग A (प्र.1) मधुमेह का उत्तर हाँ मे है।**		
a. आपको कितने वर्षों से मधुमेह है?	अवधि वर्षो/महिनो में<br>वर्ष महिन`	
b. वर्तमान में आप इसका क्या इलाज ले रहे हैं?<br>**[हाँ=1; नहीं=2]**<br>***पौराणिक औषधि, थैरपी के साथ योगा, आर्युवेद, यूनानी, होमियोपैथिक चीकित्सा, तिब्बतन, प्रकृतिक चीकित्सा, मेडिटेशन्**	निर्धारित खान–पान (आहार) संबंधी सुधार<br>निर्धारित शारीरिक व्यायाम<br>पौराणिक औषधि/ थैरपी*<br>एलोपैथीक ड्रग्स (इंगलिश/माफडर्न)<br>कोई नहीं	
c. आप कितना नियमानुसार दवाई लेते हैं?	नियमित रूप से 1<br>अक्सर भूल जाते हैं 2<br>जब उच्चरक्तचाप महसूस होता है तब ही दवाई लेते हैं 3<br>एक समय में एक महीने से ज़्यादा अनियमित हो जाते है 4<br>कभी भी दवाई नहीं लेते 5	

यदि आपके उपरोक्त प्रश्न का उत्तर "4" है तो अगले प्रश्न पर जाएं अन्यथा प्र. e पर जाएं।		
d. *अनियमितता का कारण क्या है?*	*खर्च वहन नहीं कर पाते* 1<br>*सहन नहीं कर पाते* 2<br>*स्वस्थ हो गए हैं* 3<br>*कोई कारण नहीं है* 4<br>*याद नहीं* 5<br>*अन्य (विवरण दें)* 6	***अन्य, विवरण दें।***<br>____________________
e. *क्या आपको लगता है कि आपका मधुमेह अच्छे नियंत्रण में है?*	*हाँ* 1<br>*नहीं* 2<br>*पता नहीं* 3	
f. *क्या आपको डाक्टर कहता है कि आपका मधुमेह अच्छे नियंत्रण में है?*	*हाँ* 1<br>*नहीं* 2<br>*पता नहीं* 3	
g. *आपका खाली पेट और खाने के बाद का ब्लड शुगर का स्तर क्या है जब आपने आखिरी बार जांच करवाई थी?*	*खाली पेट*________________mg/dl<br>*खाने के बाद* ________________mg/dl	
h. *आपने अपने डाक्टर से आखिरी सलाह कब ली थी?*	*एक महीने से कम* 1<br>*एक महीने से ज़्यादा* 2<br>*3 महीने से ज़्यादा* 3<br>*6 महीने से कम* 4<br>*6 महीने से ज़्यादा* 5	
i. *क्या आपके पास उच्चरक्तचाप संबंधी चिकित्सीय विवरण या औषध विधि है?*	*हाँ* 1<br>*नहीं* 2<br>*पता नहीं* 3	
j. *यदि उत्तर हाँ है तो प्रतिभागी से चिकित्सीय विवरण दिखाने को कहिए और नीचे रोग निदान लिखिए?*		
k. ***अत्यंत हाल ही कें चिकित्सीय विवरण या चिकित्सा से परतापित खाली पेट और खाने के बाद के ब्लड शुगर का स्तर को लिखिए?***<br>***खाली पेट*________________________mg/dl**<br>***खाने के बाद* ________________________mg/dl**<br><br>***भाग D भी पूरा करें***		

III. हाई ब्लड कोलेस्ट्रोल<br>यह भाग भरिए यदि भाग A (प्र॰1) हाई ब्लड कोलेस्ट्रोल का उत्तर हाँ मे है।		
a. आपको कितने वर्षों से हाई ब्लड कोलेस्ट्रोल है?	अवधि वर्षो/महिनो में<br>वर्ष महिन`	
b. वर्तमान में आप इसका क्या इलाज ले रहे हैं?<br>**[हाँ=1; नहीं=2]**<br>***पौराणिक औषधि, थैरपी के साथ योगा, आर्युवेद, यूनानी, होमियोपैथिक चीकित्सा, तिब्बतन, प्रकृतिक चीकित्सा, मेडिटेशन्**	निर्धारित खान–पान (आहार) संबंधी सुधार<br>निर्धारित शारीरिक व्यायाम<br>पौराणिक औषधि/ थैरपी*<br>एलोपैथीक ड्रग्स (इंगलिश/माफडर्न)<br>कोई नहीं	
c. आप कितना नियमानुसार दवाई लेते हैं?	नियमित रूप से 1<br>अक्सर भूल जाते हैं 2<br>जब हाई ब्लड कोलेस्ट्रोल महसूस होता है तब ही दवाई लेते हैं 3<br>एक समय में एक महीने से ज़्यादा अनियमित हो जाते है 4<br>कभी भी दवाई नहीं लेते 5	
**यदि आपके उपरोक्त प्रश्न का उत्तर "4" है तो अगले प्रश्न पर जाएं अन्यथा प्र॰ e पर जाएं।**		
d. अनियमितता का कारण क्या है?	खर्च वहन नहीं कर पाते 1<br>सहन नहीं कर पाते 2<br>स्वस्थ हो गए हैं 3<br>कोई कारण नहीं है 4<br>याद नहीं 5<br>अन्य (विवरण दें) 6	अन्य, विवरण दें।<br>____________________
e. क्या आपको लगता है कि आपका हाई ब्लड कोलेस्ट्रोल अच्छे नियंत्रण में है?	हाँ 1<br>नहीं 2<br>पता नहीं 3	
f. क्या आपको डाक्टर कहता है कि आपका हाई ब्लड कोलेस्ट्रोल अच्छे नियंत्रण में है?	हाँ 1<br>नहीं 2<br>पता नहीं 3	
g. आपके पिछले हाई ब्लड कोलेस्ट्रोल की रिकाँर्डिंग क्या थी? (जब आपकी डाक्टर ने जांच की थी)	____________(systolic) / _____________(diastolic) mmHg	
h. आपने अपने डाक्टर से आखिरी सलाह कब ली थी?	एक महीने से कम 1<br>एक महीने से ज़्यादा 2<br>3 महीने से ज़्यादा 3<br>6 महीने से कम 4<br>6 महीने से ज़्यादा 5	

i.	क्या आपके पास हाई ब्लड कोलेस्ट्रोल संबंधी चिकित्सीय विवरण या औषध विधि है?	हाँ 1<br>नहीं 2<br>पता नहीं 3	
j.	यदि उत्तर हाँ है तो प्रतिभागी से चिकित्सीय विवरण दिखाने को कहिए और नीचे रोग निदान लिखिए?		
k.	***अत्यंत हाल ही में चिकित्सीय विवरण या चिकित्सा से परतापित रक्तचाप पर संक्षिप्त लेख लिखिए?***		
**IV.**	हृदय रोग<br>***यह भाग भरिए यदि भाग A (प्र॰1) हृदय रोग का उत्तर हाँ मे है।***		
a.	आपको सबसे पहले कब पता चला कि आपको हृदय रोग है?	<1 year 1<br>1-5 years 2<br>>5 years 3	
b.	डाक्टर ने आपको हृदय रोग के बारे में क्या बताया?	दिल का दौरा 1<br>ऐन्जाइना(कण्ठ शूल) 2<br>हृदय गति का रूकना 3<br>वेल्व डिजीज़ 4<br>हृदय मे छेद 5<br>अन्य* 6<br>समस्या के बारे में सूचीत नहीं किया 7	एक से ज्याद विकल्पो के लिए पृथक बाक्सों का प्रयोग करें।<br>अन्य, विवरण दें।<br>____________________
	यदि आपके उपरोक्त प्रश्न का उत्तर "1" है तो अगले प्रश्न पर जाएं अन्यथा प्र॰ g पर जाएं।		
c.	जब आपको पहली बार हार्ट अटैक हुआ तब आपकी उम्र क्या थी?	वर्ष	
d.	क्या आपको इलाज के लिए अस्पताल में भर्ती किया गया था?	हाँ 1<br>नहीं 2	
e.	क्या आपको दोबारा उसी प्रकार का हार्ट अटैक हुआ?	हाँ 1<br>नहीं 2	
f.	क्या आपको दोबारा का हार्ट अटैक के लिए अस्पताल में भर्ती किया गया था?	हाँ 1<br>नहीं 2	
g.	वर्तमान में आप हृदय रोग का क्या इलाज ले रहे हैं?<br>***[हाँ=1; नहीं=2]***<br>****पौराणिक औषधि, थैरपी के साथ योगा, आर्युवेद, यूनानी, होमियोपैथिक चीकित्सा, तिब्बतन, प्रकृतिक चीकित्सा, मेडिटेशन्***	निर्धारित खान–पान (आहार) संबंधी सुधार<br>निर्धारित शारीरिक व्यायाम<br>पौराणिक औषधि/ थैरपी*<br>एलोपैथीक ड्रग्स (इंगलिश/माफडर्न)<br>कोई नहीं	

h. आप कितना नियमानुसार दवाई लेते हैं?	नियमित रूप से 1<br>अक्सर भूल जाते हैं 2<br>केवल दवाई जबही लेता हूँ जब अस्वस्थ महसूस करता हूँ 3<br>एक समय में एक महीने से ज़्यादा अनियमित हो जाते है 4<br>कभी भी दवाई नहीं लेते 5	
यदि आपके उपरोक्त प्रश्न का उत्तर "4 है तो प्रश्न "i" पर जाएं अन्यथा प्र॰ j पर जाएं।		
i. अनियमितता का कारण क्या है?	खर्च वहन नहीं कर पाते 1<br>सहन नहीं कर पाते 2<br>स्वस्थ हो गए हैं 3<br>कोई कारण नहीं है 4<br>याद नहीं 5<br>अन्य (विवरण दें) 6	अन्य, विवरण दें।<br>________________
j. आपने अपने डाक्टर से आखिरी सलाह कब ली थी?	एक महीने से कम 1<br>एक महीने से ज़्यादा 2<br>3 महीने से ज़्यादा 3<br>6 महीने से कम 4<br>6 महीने से ज़्यादा 5	
k. क्या आपके पास हृदय रोग संबधी चिकित्सीय विवरण या औषध विधि है?	हाँ 1<br>नहीं 2<br>पता नहीं 3	
l. यदि उत्तर हाँ है तो प्रतिभागी से चिकित्सीय विवरण दिखाने को कहिए और नीचे रोग निदान लिखिए?		
**V. घात स्ट्रोक (लकवा)**<br>***यह भाग भरिए यदि भाग A (प्र॰1) घात (लकवे का अटैक) का उत्तर हाँ मे***		
a. आपकी उम्र क्या थी जब आपको घात (लकवे का अटैक) हुआ था?	Years	
b. क्या शरीर के किसी भाग में विकलांगता शेष है?	हाँ 1<br>नहीं 2	

c. यदि हाँ तो इसमें से किस अंग पर इसका असर है? **[Yes=1; No=2]**	टांगो/पैरो का लकवा<br>बाजू/हाथ का लकवा<br>टांगो/पैरो की कमज़ोरी<br>बाजू/हाथ की कमज़ोरी<br>बोलने में परेशानी<br>देखने में परेशानी<br>पेशाब रोकने में असमर्थता<br>अन्य कोई कमज़ोरी<br>विवरण दे ______________________	
d. क्या आपको लकवे कि बाद दवाईयों को लेते रहने की सलाह दी गई है?	हाँ 1<br>नहीं 2	
e. यदि हाँ तो कितना नियमानुसार दवाई लेते हैं?	नियमित रूप से 1<br>अक्सर भूल जाते हैं 2<br>केवल दवाई जबही लेता हूँ जब अस्वस्थ महसूस करता हूँ 3<br>एक समय में एक महीने से ज़्यादा अनियमित हो जाते है 4<br>कभी भी दवाई नहीं लेते 5	
**यदि आपके उपरोक्त प्रश्न का उत्तर "4 है तो प्रश्न 'f" पर जाएं अन्यथा प्र० g पर जाएं।**		
f. अनियमितता का कारण क्या है?	खर्च वहन नहीं कर पाते 1<br>सहन नहीं कर पाते 2<br>स्वस्थ हो गए हैं 3<br>कोई कारण नहीं है 4<br>याद नहीं 5<br>अन्य (विवरण दें) 6	अन्य, विवरण दें।<br>________________
g. आपने अपने डाक्टर से आखिरी सलाह कब ली थी?	एक महीने से कम 1<br>एक महीने से ज़्यादा 2<br>3 महीने से ज़्यादा 3<br>6 महीने से कम 4<br>6 महीने से ज़्यादा 5	
h. क्या आपके पास घात (लकवे का अटैक) संबंधी चिकित्सीय विवरण या औषध विधि है?	हाँ 1<br>नहीं 2<br>पता नहीं 3	
i. यदि उत्तर हाँ है तो प्रतिभागी से चिकित्सीय विवरण दिखाने को कहिए और नीचे रोग निदान लिखिए?		

Participant ID

**VI.** *स्थायी किड्नी डिज़ीज* *यह भाग भरिए यदि भाग A (प्र.1) किडनी डिज़ीज का उत्तर हाँ मे*		
a. *किस उम्र मे आपको किडनी संबधी बीमारी का पता चला?*	*वर्ष*	
b. *वर्तमान में आप इसका क्या इलाज ले रहे हैं?* **[हाँ=1; नहीं=2]** ***पौराणिक औषधि, थेरपी के साथ योगा, आर्युवेद, यूनानी, होमियोपैथिक चीकित्सा, तिब्बतन, प्रकृतिक चीकित्सा, मेडिटेशन्**	*निर्धारित खान–पान (आहार) संबंधी सुधार* *निर्धारित शारीरिक व्यायाम* *पौराणिक औषधि / थैरपी** *एलोपैथीक ड्रग्स (इंगलिश/माफडर्न)* *कोई नहीं*	
c. *आप कितना नियमानुसार दवाई लेते हैं?*	*नियमित रूप से* 1 *अक्सर भूल जाते हैं* 2 *जब किडनी संबधी बीमारी महसूस होता है तब ही दवाई लेते हैं* 3 *एक समय में एक महीने से ज़्यादा अनियमित हो जाते है* 4 *कभी भी दवाई नहीं लेते* 5	
यदि आपके उपरोक्त प्रश्न का उत्तर "4" है तो अगले प्रश्न d पर जाएं अन्यथा प्र. e पर जाएं।		
d. *अनियमितता का कारण क्या है?*	*खर्च वहन नहीं कर पाते* 1 *सहन नहीं कर पाते* 2 *स्वस्थ हो गए हैं* 3 *कोई कारण नहीं है* 4 *याद नहीं* 5 *अन्य (विवरण दें)* 6	*अन्य, विवरण दें।* ________________
e. *क्या आपको लगता है कि आपका किडनी संबधी बीमारी अच्छे नियंत्रण में है?*	*हाँ* 1 *नहीं* 2 *पता नहीं* 3	
f. *क्या आपके पास किडनी संबधी बीमारी का चिकित्सीय विवरण या औषध विधि है?*	*हाँ* 1 *नहीं* 2 *पता नहीं* 3	
g. *यदि उत्तर हाँ है तो प्रतिभागी से चिकित्सीय विवरण दिखाने को कहिए और नीचे रोग निदान लिखिए?*		

Participant ID

PART - C: एन्जाइना (कण्ठशूल) परिधीम नाडी संबधी बीमारी (परिफरल वैस्क्यूलर ड़िजीज) और हृदय गति का रूकना

**I. एन्जाइना**		
a. क्या आपको इनमें से कोई लक्षण है? **[हाँ =1; नहीं =2]**	धडकन महसूस होना (स्पन्दन)<br>छाती में दर्द<br>संस फूलना<br>थकावट/कमज़ोरी<br>छाती में असुविधा/भारीपन/बेचैनी	**"2" for all, skip to Q. m**
b. परिश्रमी गतिविधियाँ जैसे–तेज़ चलना, सीढीयां चढ़ना, वज़न उठाना इत्यादि के दौरान क्या आपको छाती में या छाती के आसपास, भुजाओं, कंधो, गरदन, नीचले जबड़े, पेट से या पीठ के ऊपरी हिस्से में इनमें से कोई लक्षण महसूस होते है? **[हाँ =1; नहीं =2]**	दर्द<br>भारीपन<br>दबाव<br>असुविधा<br>सुन्नता	**"2" for all, skip to Q. d**
c. प्र॰(a) और (b) में बताए गए लक्षणों को आप सबसे ज़्यादा कहाँ महसूस करते हैं? **[Yes=1; No=2]** (नीचे दिए गए चित्र में बताए गए लक्षणो के स्थान को नम्बर से इनगित करे) **(अतिरिक्त न॰ छाती के पिछे = 11, गरदन के पिछे = 12)**	दर्द<br>भारीपन<br>असुविधा<br>सुन्नता	**लक्षण** / **स्थान**

Right 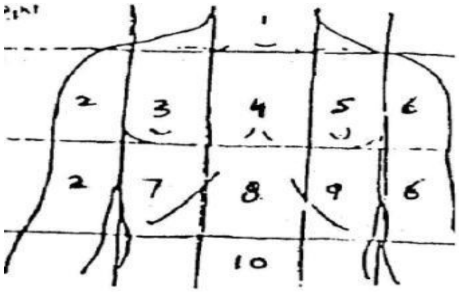 Left

d. ऊपर बताए गए लक्षणो को क्या आपने किसी ओर जगह भी महसूस करते हैं?	हाँ 1<br>नहीं 2<br><br>यदि हॉ विवरण दें।<br>लक्षण: ________________<br>स्थान: ________________	

Fill Q.e to Q.l only if you have noted "1" for any of the symptoms in Q.b and Q.c, OTHERWISE GO TO Q.m

e. जब आप सीढियाँ चढते है या चढाई पर चलते हैं या तेज़ चलते है तो क्या आपको ऊपर वर्णित कोई लक्षण महसूस होता है या आपकी सांस फूल जाता है या आपको दिल की धड़कन महसूस होने लगती हैं?	हाँ 1<br>नहीं 2<br>कभी भी चढाई पर या तेज़ नहीं चलते 3	
f. क्या इनमें से कोई लक्षण आपको समतल ज़मीन पर सामान्य गति से चलने मे भी महसूस होता है?	हाँ 1<br>नहीं 2	
g. आप सामान्य तौर पर क्या करते है यदि इनमें से कोई लक्षण आपको परिश्रम करने के दौरान महसूस होता है?	हाँ 1<br>नहीं 2	
h. क्या इनमें से कोई लक्षण आपको आराम करते समय या खाना खाने के बाद महसूस होता है?	रूक जाते हैं 1<br>धीरे चलते हैं 2<br>उसी गति से चलते रहते हैं 3	
i. यदि उस समय आप रूक जाते हैं या धीरे चलते रहते है तो क्या लक्षण खत्म हो जाते?	हाँ 1<br>नहीं 2	**"2" go to Q.k**
j. प्र॰(i) का उत्तर "हाँ" में है तो सामान्यत लक्षण कितनी देर मे खत्म हो जाते है?	< 3 mins 1<br>3-20 mts 2<br>>20 mts 3	
k. क्या आप सामान्यत लक्षणों से आराम पाने के लिए जीभ के नीचे कोई दवाई रखते हैं?	हाँ 1<br>नहीं 2	**"2" go to Q.m**
l. यदि हाँ तो कितनी देर में आराम मिलता है?	< 2mts 1<br>2-5 mts 2<br>6-10 mts 3<br>>10 mts 4	
m. क्या कभी आपने सीने में (छाती के सामने के हिस्से में) तेज़ दर्द या बेचैनी महसूस की है, जो कि लगातार आधा घंटा या ज़्यादा समय तक बनी रही हो?	हाँ 1<br>नहीं 2	**"2", go to the next section**
n. यदि हाँ तो दर्द और बेचैनी के साथ इनमें से कई अन्यलक्षण होते हैं -<br>***[Yes=1; No=2]***	ठंडे पसीने आना<br>सांस लने मे परेशानी<br>ज़्यादा पसीने आना	
o. ज्ब आपको सबसे पहली बार छाती में दर्द हुआ था तब आपकी उम्र क्या थी?	Years	
p. आपको इस प्रकार के कितने अटैक आ चुके हैं?		
q. क्या आपने कभी ECG कराई है?	हाँ 1<br>नहीं 2	

r. क्या आपने छाती मे दर्द के कारण डाक्टरी सलाह ली है?	हाँ 1<br>नहीं 2	

II. परिधीम नीड़ी संबधी बीमारी (परीफल वैस्कूलर डिज़ीज)		
a. क्या चलते समय आपकी एक या दोनो टांगो मे दर्द होता है?	हाँ 1<br>नहीं 2	**"2" go to the Part III**
b. यदि हाँ तो आप अपनी टांग के किस भाग मे दर्द महसूस करते हैं?	पिंडली के दर्द को शामिल करते है 1<br>पिंडली के दर्द को शामिल नहीं करते है 2	**"2" go to the Part III**
c. क्या यह दर्द सीढियाँ चढते समय या तेज़ चलते समय होता है?	हाँ 1<br>नहीं 2<br>लागू नहीं 3	**"2" go to the Part III**
d. क्या यह दर्द तब भी होता है जब आप समतल ज़मीन पर सामान्य गति से चलते हैं?	हाँ 1<br>नहीं 2	
e. क्या आपको कभी इस दर्द से चलते चलते राहत मिल जाती है?	हाँ 1<br>नहीं 2	**"1" go to the Part III**
f. जब चलते समय आपको दर्द होता है तब आप क्या करते हैं?	रूक जाते हैं 1<br>चलते रहते है 2	**"2" go to the Part III**
g. तब क्या होता है जब आप चलते–चलते रूक जाते है?	आराम मिलता है 1<br>आराम नहीं मिलता है 2	**"2" go to the Part III**
h. यदि आराम मिलता है तो कितने समय मे मिलता है?	10 मिनट या कम 1<br>10 मिनट से ज़्यादा 2	

III. ह्रदय गति का रूकना (हार्टफेलयर)		
a. क्या आप शारिरीक अपंगता के कारण चलने में असमर्थ है?	हाँ 1<br>नहीं 2	**"1" skip to Q. e**
b. जब आप अपने हमउम्र के लोगों के साथ समतल ज़मीन पर चलते है तो क्या आपकी सांस फूलने लगती है?	हाँ 1<br>नहीं 2	
c. जब आप चढ़ाई या सीढ़ियां चढ़ते है तो अपने हमउम्र के लोगों की अपेक्षा आपकी सांस अधिक फूलने लगती है?	हाँ 1<br>नहीं 2	**"2" go to Q. e**
d. क्या अआपको कभी सांस फूलने के कारण चलते–चलते रूकना पड़ा है?	हाँ 1<br>नहीं 2	
e. बीते सालों में सांस ठीक से न आने के कारण क्या आप किसी समय नींद से जागे है?	हाँ 1<br>नहीं 2	**"2" go to Q. g**

f. आपको ये समस्या कितने दिनों से है?	एक साल से कम 1<br>एक साल से अधिक 2	
g. क्या आपके टक्खनों (ऐंगकल) में सूजन है?	हाँ 1<br>नहीं 2	
h. क्या डॉक्टर ने आपको कभी यह बताया हैं कि आपको फेंफड़ों से संबंधित कोई बीमारी है (COPD, अस्थमा आदि)	हाँ 1<br>नहीं 2	
i. क्या आपके शरीर में कोई हृदयंत्र लगा हुआ है?	हाँ 1<br>नहीं 2	
j. यदि हां तो यंत्र का नाम बबताइए?	सामान्य गतिनिर्धारक (स्टेन्डर्ड पेस्मैकर) 1<br>इम्पलैन्टेबल कार्डियोवर्टर डेफीब्रिलेटर (ICD) 2<br>कार्डीएक रिसिनक्रोनाइसेशन थैरेपी डिवाईस डेफीब्रिलेटर के साथ (CRT-D) 3<br>कार्डीएक रिसिनक्रोनाइसेशन थैरेपी डिवाईस डेफीब्रिलेटर रहित (CRT-P) 4	
**PART - D: काम्पीलीकेशन**		
यदि आपने Part –B को भरा है तो निम्नलिखित section को पूरा किजिए !		
**I. पांव को फोडा** (पांव का अहसर) (फुट अहसर) और ऐम्पुटेशन (अंगोच्छेदन)		
a. क्या आपको कभी पांव के न भरने वाला फोडा या घाव हुआ है जिसके भरने में 4 हफ्ते से ज़्यादा समय लगा हो?	हाँ 1<br>नहीं 2	
b. क्या आप यहां वहां नंगे पैर चले है?	हाँ 1<br>नहीं 2	
c. क्या आपने कभी ऐम्पुटेशन् (अंगोच्छेन) करवाया है?	हाँ 1<br>नहीं 2	**“2” go to Part II**
d. यदि हां तो कब	वर्षो पहले (या) महीनो पहले	
e. ऐम्पुटेशन् (अंगोच्छेन) का स्तर पंजा (अंगोच्छेन)	पांव का अंगूठा 1<br>टक्खने के नीचे 2<br>घुटने के नीचे 3<br>घुटने के ऊपर 4	
f. ऐम्पुटेशन् (अंगोच्छेन) का कारण क्या था?	ज़ख्म (चोट) 1<br>मधुमेह 2<br>संक्रमण 3	अन्य, विवरण दें।<br>____________

	अन्य 4	
g. क्या आपके पास चिकित्सीय विवरण या औषध–निधि है?	हाँ 1<br>नहीं 2<br>पता नहीं 3	
h. यदि उत्तर हां में है तो प्रतिभागी से चिकित्सीय विवरण दिखाने के लिए कहिए और नीचे रोग–निदान लिखिए!		
**II. आँखें**		
a. क्या आपको चश्मा लगाने के अलावा अपनी नेत्र दृष्टि में कोई परेशानी है?	हाँ 1<br>नहीं 2	**"2" skip the section**
b. यदि हाँ तो क्या आपको डॉक्टर ने कहाँ है कि आपकी कमजोर नेत्र दृष्टि मधुमेह की समसया के कारण है?	हाँ 1<br>नहीं 2	**"2" skip the section**
c. यदि हाँ तो क्या रोग–निदान था?		
d. क्या आप किसी समय लेज़र थैरपी से गुजरे है?	हाँ 1<br>नहीं 2	
e. क्या आपके पास किड्नी संबधी बीमारी का चिकित्सीय विवरण या औषध विधि है?	हाँ 1<br>नहीं 2<br>पता नहीं 3	
f. यदि उत्तर हाँ है तो प्रतिभागी से चिकित्सीय विवरण दिखाने को कहिए और नीचे रोग निदान लिखिए?		
**PART – E:** श्वास संबंधी बीमारी (रेस्पिरेटरी डिज़ीज)		
**1-** पिछले 12 महीनों के अंदर हफ्ते में, अधिकतम दिन या रात में (लगातार कम से कम 3 महीनों के दौरान) क्या आपको दीर्घस्थायी कफ और बलगम की उत्पति हुई है? **[नहीं = 1; नहीं = 2]**<br>**कफ** से तात्पर्य है कि केवल आप जुकाम से ही पीड़ित नही है!<br>**अधिकतम** से तात्पर्य है कम से कम 4 दिन या रात प्रतिसप्ताह!		
a. यदि हाँ — i. पिछले 12 महिनों में इस तरह के कफ़ ने आपको कितनी बार परेशान किया ?<br>ii. पिछले वर्ष क्या आप किसी ऐसे संक्रमण से पीड़ित थे जिसके लिए आपको चिकित्सीय देख–रेख की आवश्यकता थी?<br>iii. पिछले 12 महिनों में क्या आपको कितनी बार चिकित्सीय देख–रेख की आवश्यकता पड़ी?		
2. पिछले 12 महिनों में क्या आप छाती के संक्रमण (पेस्ट इनफैक्शन) (टी. बी. छोडकर रहित) के लिए डॉक्टर या स्वास्थ्य चिकित्सक से मिलें है? **[हाँ=1; नहीं=2]**		

a. यदि हाँ — i. पिछले 12 महिनों में कितनी बार डॉक्टर से मिलें?<br>ii. डॉक्टर ने निदान कितनी बार किया?<br>iii. यह संक्रमण कितने समय तक रहा?<br>iv. क्या आने इन संक्रमणों के लिए एन्टाइबाइआटिक दवाइयों का सेवन किया था?<br>**[हाँ=1; नहीं=2]**	 <br>Yrs Mnts
3. पिछले 12 महिनों में क्या आप छाती के संक्रमण या निमोनिया के लिए अस्पताल में भर्ती थे?<br>**[हाँ=1; नहीं=2]**	
a. यदि हाँ, रूकने की अवधि!	दिन<br>हफ्ते<br>महिने
4. क्या आप वर्तमान में दमें से पीड़ित है?<br>**[हाँ=1; नहीं=2]**	
a. यदि हाँ — i. पिछले 12 महिनों में आपको कितनी बार अस्थमें/दमे का अटैक आया है?<br>ii. पिछले वर्ष क्या आप किसी ऐसे संक्रमण से पीड़ित है जिसके लिए आपको चिकित्सीय देख–रेख की आवश्यकता थी?<br>iii. पिछले 12 महिनों में क्या आपको कितनी बार चिकित्सीय देख–रेख की आवश्यकता पड़ी?	

PART – F: फैमली हिस्ट्री (परिवार का विवरण)

1. क्या आपके परिवार मे से कोई भी 60 साल की उम्र से पहले नित्नलिखित बीमारीयों में से किसी बीमारी से पीड़ित था?<br>**[हाँ=1; नहीं=2; पता नहीं=3]**	उच्चरक्तचाप<br>हृदय रोग*<br>मधुमेह<br>घात (लकवे का अटैक)<br>***एन्जाइना, दिल का दौरा, हृदय गति का स्कना**	

2. नीचे दी गई तालिका भरें

**परिवार के सदस्यों से संबध**	**रोग के प्रकार (नीचे दी गए कोड से उल्लेख)**	**उम्र निदान के समय (वर्षो में)**	**यदि मृत्यु हुई तो किस उम्र में**
पिता			
माता			
पुत्र			

पुत्री			
दादा			
दादी			
नाना			
नानी			
भाई			
बहन			
चाचा			
चाची			
मामा			
मामी			
अन्य के लिए कृपया प्रतिभागी का संबंध और आवश्यक विवरण नीचे लिखें।			

**** रोग की दशा (स्थिति): मधुमेह = 1, हृदय रोग = 2, उच्चरक्तचाप = 3, घात (लकवे का अटैक) = 4***

SECTION – 6: *इलाज (चिकित्सा) का विवरण और खर्च (प्यय)*

PART A: *बाहरी रोगी*

1. *क्या आप निम्नलिखित में से किसी कारण के लिए बाहरी (बाह्रय) रोगी के रूप्प में इलाज करा रहे है?*<br><br>***[Yes=1; No=2]***	*हृदय रोग*<br>*लकवा*<br>*मधुमेह*<br>*मधुमेह पैयीदगी (संक्रमण, रेटिनोपैथी, येफोपैथी आदि)*<br>*उच्चरक्तचाप*<br>*किड़नी डिज़ीज*	

यदि ऊपर दिए गए विकल्पों में से किसी का उत्तर हाँ में है, तो अगले भाग में जाए अन्यथा भाग– ठ छोड़ दें।

Participant ID

निम्नलिखित प्रश्नों में केवल पिछले 12 महीनों के लिए प्रतिभागी के इलाज का विवरण और खर्च के बारे में पूछिए।		
2. पिछले 12 महीनों में आपने कितनी बार चिकित्सीय सुविधा/डॉक्टर/ थेरेपिस्ट का दौरा किया?		
3. चिकित्सीय सुविधा/डॉक्टर/थेरेपिस्ट के प्रकार सरकारी, गैर सरकारी धमार्थ संगठन, अन्य (विवरण दें)	सरकारी 1<br>गैर–सरकारी 2<br>धमार्थ 3<br>अन्य 4	अन्य, विवरण दें।<br>__________

4. प्रत्येक तालिका में पृथक–पृथक ऊपर उल्लिखित बीमारियों (प्र0 –1) के खर्चो का सूचीबद्ध कीजिए!

4.i. रोग ______________________

व्यय का स्वरूप	आवृति	कुल प्रयुक्त राशि रू0 में प्रति दौरा/ जांच/ वेतन (गृह नर्स या देख रेख करने वाले के लिए)
डॉक्टर के पास दौरा लगाया (फीस)		
नर्स या देख रेख करने वाले का किराये पर रखना (महीने में)		
जांच		
शारीरिक या व्यवसायिक पुनःस्थापन		
अन्य (विवरण दें)______________________________		
दवाईया, (पिछले 12 महीनों में ऊपर दी गई उल्लिखित बीमारियों के लिए औसतन कुल प्रयुक्त राशि)		
**पिछले 12 महीनों में कुल व्यय**		

4.ii. रोग ______________________

व्यय का स्वरूप	आवृति	कुल प्रयुक्त राशि रू0 में प्रति दौरा/ जांच/ वेतन (गृह नर्स या देख रेख करने वाले के लिए)
डॉक्टर के पास दौरा लगाया (फीस)		
नर्स या देख रेख करने वाले का किराये पर रखना (महीने में)		
जांच		
शारीरिक या व्यवसायिक पुनःस्थापन		
अन्य (विवरण दें)______________________________		
दवाईया, (पिछले 12 महीनों में ऊपर दी गई उल्लिखित बीमारियों के लिए औसतन कुल प्रयुक्त राशि)		
**पिछले 12 महीनों में कुल व्यय**		

4.iii. रोग ____________________

व्यय का स्वरूप	आवृति	कुल प्रयुक्त राशि रू0 में प्रति दौरा/ जांच/ वेतन (गृह नर्स या देख रेख करने वाले के लिए)
डॉक्टर के पास दौरा लगाया (फीस)		
नर्स या देख रेख करने वाले का किराये पर रखना (महीने में)		
जांच		
शारीरिक या व्यवसायिक पुनःस्थापन		
अन्य (विवरण दें)____________________________		
दवाईया, (पिछले 12 महीनों में ऊपर दी गई उल्लिखित बीमारियों के लिए औसतन कुल प्रयुक्त राशि)		
**पिछले 12 महीनों में कुल व्यय**		

4.iv. रोग ____________________

व्यय का स्वरूप	आवृति	कुल प्रयुक्त राशि रू0 में प्रति दौरा/ जांच/ वेतन (गृह नर्स या देख रेख करने वाले के लिए)
डॉक्टर के पास दौरा लगाया (फीस)		
नर्स या देख रेख करने वाले का किराये पर रखना (महीने में)		
जांच		
शारीरिक या व्यवसायिक पुनःस्थापन		
अन्य (विवरण दें)____________________________		
दवाईया, (पिछले 12 महीनों में ऊपर दी गई उल्लिखित बीमारियों के लिए औसतन कुल प्रयुक्त राशि)		
**पिछले 12 महीनों में कुल व्यय**		

5. क्या आपको बीमा से कोई क्षतिपूर्ति मिला?	हाँ 1<br>नही 2<br>जानते नही 3<br>कोई बीमा नही करवाया 4	
6. यदि हाँ तो ऊपर उल्लिखित खर्चो में कितनी क्षतिपूर्ति मिली (रू0 में)	RS ______________	
7. चिकित्सीय सुविधा/डॉक्टर/ थेरेपिस्ट के पास पहुँचने में जिया गया समय ———	__ __ : __ __<br>घंटें मिनट	
8. ऊपर दी गई उल्लिखित चिकित्सीय सुविधा/डॉक्टर/थेरेपिस्ट का दौरा करने के लिए परिवहन लागत*	RS ______________	

*यदि प्रतिभागी के पास अपना वाहन है तो उससे कहिए की यात्रा में प्रयुक्त ईधन का अनुमानित खर्च बताए!

9. चिकित्सीय सुविधा में प्रयुक्त औसतन समय।	HR : MIN		
10. मैं सही चिकित्सीय सुविधा प्राप्त कर रहा हूँ?	चिकित्सीय सुविधा प्राप्त करने में आपको क्या रूकावट है?	0	अन्य, विवरण दें। ________________
	उपलब्ध नही है	1	
	प्राप्त करने के लिए कोई मदद नही करता	2	
	बहुत दूर है	3	
	बहुत खर्चीला है	4	
	खर्च नही करना चाहते	5	
	रोगी की असरल चिकित्सा प्रक्रिया	6	
	बहुत अधिक प्रतिक्षा करना	7	
	यात्रा करने में बहुत कमजोर है	8	
	चिकित्सीय देखरेख में विश्वास नही	9	
	नही जानते कहाँ जाना है	10	
	अन्य (विवरण दें)	11	
11. आप अपने इलाज और दौरे का भुगतान कैसे करते है? **[हाँ=1; नहीं=2]**	अपनी बचत से	1	
	परिवार के सदस्य भुगतान करते है	2	
	नियोक्ता द्वारा भुगतान	3	
	दोस्तो, रिश्तेदारो और नियोक्ता से उधार लेकर	4	
	बैंक से	5	
	मकान–जमीन या अन्य सम्पत्ति बेचकर	6	
	स्वास्थ्य बीमा द्वारा	7	
	अन्य (विवरण दें) ________________	8	
12. ऊपर उल्लिखित साधनों से आप कि औसतन किस अनुपात में राशि (प्रतिशत में) अपने इलाज और दौरे के लिए खर्च करते हैं?	अपनी बचत से	1	__________%
	परिवार के सदस्य भुगतान करते है	2	__________%
	नियोक्ता द्वारा भुगतान	3	__________%
	दोस्तो, रिश्तेदारो और नियोक्ता से उधार लेकर	4	__________%
	बैंक से से उधार लेकर	5	__________%
	मकान–जमीन या अन्य सम्पत्ति बेचकर	6	__________%
	स्वास्थ्य बीमा द्वारा	7	__________%
	अन्य (विवरण दें) ________________	8	__________%

PART B: INPATIENT

1. पिछले 12 महीनें में किसी बीमारी के लिए क्या आप अस्पताल में भर्ती थे?	हाँ	1	**“2” go to Q.4**
	नही	2	
	याद नहीं	3	

Participant ID

2. यदि हाँ तो कितने समय के लिए?		
3. क्या आप निम्नलिखित कारणों में से किसी कारण के लिए अस्पताल में भर्ती थे?<br><br>**[हाँ=1; नहीं=2]**	हृदय रोग<br>लकवा<br>मधुमेह<br>मधुमेह संबंधी जटिलता (संक्रमण, रिटीनोपैथी नेफरोपैथी आदि)<br>उच्च रक्तचाप<br>स्थायी किड्नी रोग	
4. पिछले 12 महीनें में क्या आप किसी शल्य चिकित्सा प्रक्रिया से गुजरे है? (यदि नही)	हाँ 1<br>नही 2<br>याद नहीं 3	**"2" go to Q.6**
5. यदि हाँ तो प्रक्रिया क्या थी?<br><br>**[हाँ=1; नहीं=2]**	रिवेस्क्यूलेराईसेशन/बाईपास<br>वेल्व रिपेयर/रिपलेसमेंट<br>पैस्मेकर<br>एमप्यूटेशन<br>एब्सश<br>रिनेल ट्रान्सप्लानटेशन<br>हार्ट ट्रान्सप्लानट<br>रिटिनल फोटोकॉग्यूलेशन<br>अन्य (विवरण_____________________)	
6. क्या आपके पास अस्पताल में भर्ती या शल्य चिकित्सा प्रक्रिया से संबंधित विवरण दे?	हाँ 1<br>नही 2	

यदि उत्तर हाँ में है तो प्रतिभागी को उसके चिकित्सीय विवरण दिखाने के लिए कहिए और बीमारी के कारण अस्पताल में भर्ती की प्रक्रिया और शल्य चिकित्सा प्रक्रिया का अलग–अलग क्रमकाल के अनुसार नीचे दी गई खाली जगह में निदान लिखिए।

अस्पताल में भर्ती की प्रक्रिया

____

____

____

____

____

शल्य चिकित्सा प्रक्रिया

____

____

____

____

____

टिप्पणी

PART C: *अस्पताल में भर्ती की लागत (खर्च)*

यह भाग केवल जब ही भरिए यदि, प्रतिभागी बीमारी के कारण अस्पताल में भर्ती की प्रक्रिया या प्र0–1 और प्र0–4 (Part –B) *में उल्लिखित प्रक्रिया से गुजरे है, अन्यथा साक्षात्कार समाप्त कीजिए और प्रतिभागी को धन्यवाद कहिए!*

पिछले 12 महीनों में प्रथम बार अस्पताल में भर्ती होने की प्रक्रिया से शुरूकर प्रत्येक प्रक्रिया का निम्नलिखित विवरण दें। यदि आप अस्पताल में 3 से ज्यादा बार भर्ती हुए है तो विवरण को पूर्ण करने के लिए दूसरे फार्म का प्रयोग करे।

क्र0 सं0	प्रश्न	1	2	3
1	आप अस्पताल में भर्ती कब हुए थे?	महिना	महिना	महिना
2	आपने अस्पताल में कितने दिन बिताए?	दिन	दिन	दिन
3	अस्पताल का स्वरूप?<br><br>**[हाँ=1; नहीं=2]**	सरकारी<br>गैर सरकारी<br>धर्मार्थ संगठन<br>अन्य (विवरण दें)	सरकारी<br>गैर सरकारी<br>धर्मार्थ संगठन<br>अन्य (विवरण दें)	सरकारी<br>गैर सरकारी<br>धर्मार्थ संगठन<br>अन्य (विवरण दें)
4	अस्पताल का नाम–पता			

5	आप किस प्रकार के इलाज/प्रक्रिया/शल्य चिकित्सा से गुजरे है?<br><br>**(चिकित्सीय विवरण के साथ क्रॉस चैक कीजिए और Part –A से सूचना लीजिए)**<br><br>**[हाँ=1; नहीं=2]**	दवाईयॉ<br>थ्रोम्बोलिसिस<br>एन्जीयोग्रम<br>एन्जीयोप्लास्टी<br>बाईपास सर्जरी<br>ब्रेकीथैरेपी<br>पैस्मेकर<br>हार्ट ट्रांसप्लान्ट<br>एमप्यूटेशन<br>इकोकार्डीयोग्रफी<br>न्यूरो इमेजिंग<br>डॉयलिसिस<br>किड्नी ट्रांसप्लान्ट<br>आब्ज़र्वेशन के लिए<br>अन्य प्रोसीजर<br>विवरण __________	दवाईयॉ<br>थ्रोम्बोलिसिस<br>एन्जीयोग्रम<br>एन्जीयोप्लास्टी<br>बाईपास सर्जरी<br>ब्रेकीथैरेपी<br>पैस्मेकर<br>हार्ट ट्रांसप्लान्ट<br>एमप्यूटेशन<br>इकोकार्डीयोग्रफी<br>न्यूरो इमेजिंग<br>डॉयलिसिस<br>किड्नी ट्रांसप्लान्ट<br>आब्ज़र्वेशन के लिए<br>अन्य प्रोसीजर<br>विवरण __________	दवाईयॉ<br>थ्रोम्बोलिसिस<br>एन्जीयोग्रम<br>एन्जीयोप्लास्टी<br>बाईपास सर्जरी<br>ब्रेकीथैरेपी<br>पैस्मेकर<br>हार्ट ट्रांसप्लान्ट<br>एमप्यूटेशन<br>इकोकार्डीयोग्रफी<br>न्यूरो इमेजिंग<br>डॉयलिसिस<br>किड्नी ट्रांसप्लान्ट<br>आब्ज़र्वेशन के लिए<br>अन्य प्रोसीजर<br>विवरण __________
6	इलाज पर प्रयुक्त कुल राशि (अस्पताल में भर्ती होने का खर्च, अस्पताल में यकने के दौरान खरीदी गई दवाईयाँ)	Rs _______________	Rs _______________	Rs _______________
7	अस्पताल में सहायक (ऐटेन्डेंट) ने आपके साथ कितने दिन बिताये	दिन	दिन	दिन
8	सहायक के रूकने की लागत (खाना, आवास सुविधा और यात्रा)	Rs ______________	Rs ___________	Rs ___________
9	घर से अस्पताल तक की दूरी ।	Kms	Kms	Kms
10	घर से अस्पताल तक का यात्रा खर्च ।<br>(ऐम्बुलेन्स का खर्च अलग से, यदि कोई हैं)।	Rs _________________	Rs _________________	Rs _________________

11	आपने किस प्रकार का मैडिकल बीमा करवाया है! **[हाँ=1; नहीं=2]**	मुफ़्त मैडिकल इलाज<br>अवसायिक बीमा<br>कोई नहीं<br>स्वयं भुगतान<br>अन्य (________________)	मुफ़्त मैडिकल इलाज<br>अवसायिक बीमा<br>कोई नहीं<br>स्वयं भुगतान<br>अन्य (________________)	मुफ़्त मैडिकल इलाज<br>अवसायिक बीमा<br>कोई नहीं<br>स्वयं भुगतान<br>अन्य (____________)
12	स्वास्थ्य बीमा से कुल से कुल राशि की क्षतिपूर्ति, यदि कोई हो!	Rs __________________	Rs __________________	Rs __________________
13	आप अस्पताल मे भर्ती की लागत का कैसे भुगतान करते है **[हाँ=1; नहीं=2]**	अपनी बचत से<br>परिवार के सदस्य भुगतान करते है<br>नियोक्ता द्वारा भुगतान<br>दोस्तो, रिश्तेदारो और नियोक्ता से उधार लेकर<br>बैंक से से उधार लेकर<br>मकान–जमीन या अन्य सम्पत्ति बेचकर<br>स्वास्थ्य बीमा द्वारा<br>अन्य (विवरण दें) ________________	अपनी बचत से<br>परिवार के सदस्य भुगतान करते है<br>नियोक्ता द्वारा भुगतान<br>दोस्तो, रिश्तेदारो और नियोक्ता से उधार लेकर<br>बैंक से से उधार लेकर<br>मकान–जमीन या अन्य सम्पत्ति बेचकर<br>स्वास्थ्य बीमा द्वारा<br>अन्य (विवरण दें) ________________	अपनी बचत से<br>परिवार के सदस्य भुगतान करते है<br>नियोक्ता द्वारा भुगतान<br>दोस्तो, रिश्तेदारो और नियोक्ता से उधार लेकर<br>बैंक से से उधार लेकर<br>मकान–जमीन या अन्य सम्पत्ति बेचकर<br>स्वास्थ्य बीमा द्वारा<br>अन्य (विवरण दें) ________________
14	ऊपर उल्लिखित साधनों से आप कि औसतन किस अनुपात में राशि (प्रतिशत में) अपने इलाज और दौरे के लिए खर्च करते हैं?	अपनी बचत से________ %<br>परिवार के सदस्य भुगतान करते है ________________%<br>नियोक्ता द्वारा भुगतान______ %<br>दोस्तो, रिश्तेदारो और नियोक्ता से उधार लेकर __________%<br>बैंक से से उधार लेकर _____%<br>मकान–जमीन या अन्य सम्पत्ति बेचकर ________________%<br>स्वास्थ्य बीमा द्वारा ____%<br>अन्य ________________%<br>(विवरण दें ___________)	अपनी बचत से________ %<br>परिवार के सदस्य भुगतान करते है ________________%<br>नियोक्ता द्वारा भुगतान______ %<br>दोस्तो, रिश्तेदारो और नियोक्ता से उधार लेकर __________%<br>बैंक से से उधार लेकर ______%<br>मकान–जमीन या अन्य सम्पत्ति बेचकर ______________%<br>स्वास्थ्य बीमा द्वारा ____%<br>अन्य ________________%<br>(विवरण दें ___________)	अपनी बचत से________ %<br>परिवार के सदस्य भुगतान करते है ________________%<br>नियोक्ता द्वारा भुगतान______ %<br>दोस्तो, रिश्तेदारो और नियोक्ता से उधार लेकर __________%<br>बैंक से से उधार लेकर ______%<br>मकान–जमीन या अन्य सम्पत्ति बेचकर __________%<br>स्वास्थ्य बीमा द्वारा ____%<br>अन्य ________________%<br>(विवरण दें ___________)

15. साक्षात्कार का समय समाप्त : HR MIN

Center of Excellence - Center for Cardio-metabolic Risk Reduction in South Asia (COE-CARRS)

Questionnaire for Individuals Aged 20 Years & above

جنوبی ایشیاء میں دل کی بیماریوں کے متعلق تحقیق

ڈیپارٹمنٹ آف کمیونٹی ہیلتھ سائنسز آغا خان یونیورسٹی کراچی

انٹرویو لینے والے کیلئے ہدایات:۔ جب تک اجازت نامہ پر دستخط نہ ہو جائیں انٹرویو شروع نہ کریں

شرکت کنندہ کا نمبر ☐☐☐☐☐ گھرانہ نمبر ☐☐☐☐☐

انٹرویور کا نمبر ☐☐☐☐ کلسٹر نمبر ☐☐☐

انٹرویو شروع کرنے کا وقت منٹ ☐☐ گھنٹے ☐☐ انٹرویو کی تاریخ ☐☐☐☐☐☐

SECTION-1

DEMOGRAPHIC, SOCIO-ECONOMIC AND RESIDENTIAL DETAILS

آبادیاتی، سماجی، اقتصادی اور رہائشی تفصیلات

نمبر شمار	سوالات	کوڈز	جوابات
1	شرکت کنندہ کا پورا نام:............		
2	والد/شوہر کا پورا نام:............		
3	پتہ:............		
5	فون نمبر:............	گھر کا:<br>دفتر کا..<br>موبائل:	☐☐☐☐☐☐☐☐☐☐☐<br>☐☐☐☐☐☐☐☐☐☐☐<br>☐☐☐☐☐☐☐☐☐☐☐
6	ای میل ایڈریس:............		
7	جائے پیدائش:............	ضلع<br>صوبہ	
8	عمر:۔ (مکمل سالوں میں)............		☐☐☐
9	تاریخ پیدائش:۔ (اگر معلوم ہو)............		☐☐☐☐☐☐☐☐
10	جنس:............	1۔مرد<br>2۔عورت	☐
11	آپکی ازدواجی حیثیت کیا ہے؟............	1غیر شادی شدہ<br>2۔شادی شدہ<br>3۔ بیوہ/ رنڈوہ<br>4۔علیحدگی/طلاق یافتہ<br>5۔دیگر	☐<br>(دیگر کی وضاحت کریں)<br>............

12	آپکی مادری زبان کونسی ہے؟.....................	2۔ بلوچی<br>3۔ بنگالی<br>4۔ گجراتی<br>6۔ ہندکو<br>8۔ کشمیری<br>11۔ مراٹھی<br>12۔ میمنی<br>13۔ پشتو<br>14۔ پنجابی<br>15۔ سندھی<br>18۔ اردو<br>19۔ دیگر	☐☐<br>(دیگر کی وضاحت کریں)<br>................................
13	آپ کس مذہب سے تعلق رکھتے ہیں؟.....................	1۔ ہندو<br>2۔ مسلم<br>3۔ سکھ<br>4۔ عیسائی<br>7۔ کوئی مذہب نہیں<br>8۔ دیگر<br>9۔ جواب نہیں دیا	☐<br>(دیگر کی وضاحت کریں)<br>................................
14	کیا آپکا کسی خاص قبیلے یا ذات سے تعلق ہے؟	1۔ ہاں<br>2۔ نہیں<br>3۔ پتہ نہیں<br>4۔ بتانا نہیں چاہتے<br>5۔ لاگو نہیں ہوتا	☐
15	آپ نے *باقاعدہ تعلیم کتنے سال حاصل کی؟.....................<br>*کسی بھی تعلیمی ادارے سکول، کالج، مدرسہ، یونیورسٹی وغیرہ)		سال ☐☐
16	آپکی تعلیمی حیثیت کیا ہے؟.....................<br>* وہ شخص جو کوئی بھی زبان سمجھ کر پڑھنا اور لکھنا جانتا ہو لیکن کوئی باقاعدہ تعلیم حاصل نہیں کی۔<br>** وہ شخص جو نہ لکھ، پڑھ سکتا ہو یا صرف پڑھ سکتا ہو لیکن کسی بھی زبان میں لکھ نہیں سکتا ہو۔<br>3	1۔ پوسٹ گریجویٹ (پروفشینل)<br>2۔ گریجویٹ، ڈپلومہ B.A,B.Com,B.Sc<br>3۔ سیکنڈری سکول/ انٹر<br>4۔ ہائی اسکول (مڈل جماعت)<br>5۔ پرائمری اسکول (پانچویں جماعت تک)<br>6*۔ پڑھ لکھ سکتے ہیں لیکن کسی ادارے سے باقاعدہ تعلیم حاصل نہیں کی۔<br>7**۔ غیر تعلیم یافتہ<br>8۔ دیگر	☐<br>(دیگر کی وضاحت کریں)<br>................................

17	آپکا روزگار/نوکری کیا ہے؟..	1۔ملازم پیشہ<br>2۔طالبعلم<br>3۔گھریلو خاتون<br>4۔ریٹائرڈ<br>5۔بے روزگار	☐<br>اگر جواب 1 ہو تو سوال نمبر 17a پوچھیں ورنہ سوال نمبر 18 پوچھیں
17a	اگر آپ برسر روزگار ہیں تو آپ کا موجودہ پیشہ کیا ہے؟....................<br>(نیچے دیئے گئے کوڈز میں سے اپنے پیشے کے مطابق قریب ترین کوڈ استعمال کریں)		☐
18	کیا آپ پچھلے دس سالوں میں کسی اور پیشے سے منسلک رہے ہیں؟..........................	1۔ہاں<br>2۔نہیں	☐<br>(اگر جواب 2 ہے تو سوال نمبر 19 پر جائیں)
18a	اگر ہاں تو پیشے کا نام بتائیں؟..<br>(نیچے دیئے گئے کوڈز میں سے جو پیشہ آپ نے زیادہ عرصہ کے لئے اپنایا ہوا اپنے پیشے کے مطابق قریب ترین کوڈ استعمال کریں)		☐
ملازمت کے کوڈز:<br>1۔پروفیشنل، زمیندار، یونیورسٹی استاد، کلاس 1 آفیسر، وکیل<br>2 ۔کلرک، متوسط درجہ کا کاروبار، متوسط درجہ کا کاشتکار، استاد، انچارج/منیجر<br>3۔تربیت یافتہ مزدور، کم آمدن والا کاروبار کم رقبہ پر کاشت کرنے والا کاشتکار<br>4۔کم تربیت یافتہ مزدور، کم رقبہ والا زمیندار، رکشہ ڈرائیور، فوجی جوان، پلمبر، بڑائی (کارپینٹر)<br>5۔غیر تربیت یافتہ مزدور، بے زمین ہاری			
19	آپکے گھرانے کی کل ماہانہ آمدنی کتنی ہے؟...<br>(برائے مہربانی ان سب افراد کی آمدنی شامل کریں جو گھر کے اخراجات میں حصہ ڈالتے ہیں)	1۔3000 سے کم<br>2۔3000 سے 10,000<br>3۔10,001 سے 20,000<br>4۔20,001 سے 30,000<br>5۔30,001 سے 40,000<br>6۔40,001 سے 50,000<br>7۔50,000 سے زائد<br>8۔بتانے سے انکار کر دیا<br>9۔پتہ نہیں	☐
20	کیا آپکے گھر میں کھانا پکانے کیلئے الگ جگہ (کچن) ہے؟.....................................	1۔ہاں<br>2۔نہیں	☐
21	کھانا پکانے کیلئے کونسا ایندھن استعمال کرتے ہیں؟...<br>(جو ایندھن سب سے زیادہ استعمال کرتے ہیں وہ نوٹ کریں۔)	1۔مٹی کا تیل، کوئلہ<br>2۔بجلی، ایل پی جی گیس، سورج کی روشنی سے، سی این جی گیس<br>3۔لکڑی، گوبر<br>4۔دیگر	☐<br>(دیگر کی وضاحت کریں)<br>..

22	آپکے گھر میں پینے کے پانی کا کیا ذریعہ ہے؟..................<br>(اگر ایک سے زیادہ ذرائع ہیں تو جو سب سے زیادہ ذریعہ استعمال کیا جاتا ہے اسے نوٹ کریں۔)	1۔ گھر میں لگا سرکاری نلکہ (پائپ والا)<br>2۔ محلے میں لگا مشترکہ نلکہ (پائپ والا)<br>3۔ گھر میں لگا ذاتی نلکہ (ہینڈ پمپ)<br>4۔ بوتل کا پانی (منرل)<br>5۔ فلٹر کا پانی (گھر کا یا باہر کا)<br>6۔ دیگر	☐<br>(دیگر کی وضاحت کریں)<br>..................................
23	آپ کونسا بیت الخلاء (ٹوئلیٹ / باتھ روم) استعمال کرتے ہیں؟..................	1۔ سرکاری بیت الخلاء<br>2۔ مشترکہ بیت الخلاء (محلے میں)<br>3۔ ذاتی بیت الخلاء (گھر کے اندر)<br>4۔ دیگر	☐<br>(دیگر کی وضاحت کریں)<br>..................................
24	کیا آپکے گھر میں درج ذیل اشیاء موجود ہیں؟..................	1۔ہاں 2۔نہیں<br>a ۔ ٹی وی<br>b۔ فریج<br>c۔ واشنگ مشین<br>d۔ مائیکرو ویو آون<br>e۔ مکسر گرائینڈر<br>f۔ موبائل فون<br>g۔ ڈی وی ڈی پلیئر<br>h۔ کمپیوٹر<br>i۔ گاڑی / کار<br>j۔ موٹر سائیکل / سکوٹر<br>k۔ سائیکل	☐<br>☐<br>☐<br>☐<br>☐<br>☐<br>☐<br>☐<br>☐<br>☐<br>☐
25	کیا ایک یا دو سال کے درمیان آپ کا موجودہ رہائش سے کہیں اور منتقل ہونے کا کوئی امکان ہے؟..................	1۔ ہاں<br>2۔ نہیں<br>3۔ پتہ نہیں	☐
26	اگر آپ موجودہ رہائش سے کہیں اور منتقل ہوتے ہیں تو ہم کن افراد سے رابطہ کر کے آپکی نئی رہائش کا پتہ اور ٹیلیفون نمبر حاصل کر سکتے ہیں؟..................<br>(دو افراد کی تفصیلات لیں۔)	1۔ پڑوسی<br>2۔ رشتہ دار<br>3۔ دوست<br>4۔ جن کے ہاں کام کرتے ہیں (ایمپلائر)<br>5۔ کوئی نہیں<br>6۔ دیگر	پہلا ☐<br>دوسرا ☐<br>(دیگر کی وضاحت کریں)<br>..................................
27	پہلے فرد کا نام جس سے رابطہ کیا جا سکے؟..................		
28	پتہ:..................		

29	فون نمبر:............................	فون نمبر گھر کا:<br>فون نمبر دفتر کا :<br>موبائل نمبر :	
30	دوسرے فرد کا نام جس سے رابطہ کیا جا سکے ؟		
31	پتہ:............................		
32	فون نمبر :............................	فون نمبر گھر کا:<br>فون نمبر دفتر کا :<br>موبائل نمبر :	

SECTION-2

TOBACCO AND ALCOHOL CONSUMPTION, DIETARY HABITS, PHYSICAL ACTIVITY AND SLEEP

تمباکو نوشی، شراب نوشی، خوراک، ورزش اور نیند کے بارے میں معلومات

PART-A

تمباکو کا استعمال

1	کیا آپ نے کبھی تمباکو کا استعمال کیا ہے؟............................	1-ہاں<br>2۔ نہیں	(اگر جواب 2ہو تو سوال نمبر 8 پر جائیں)
2	آپ نے تمباکو کا استعمال کس طرح کیا ہے؟............................	1-ہاں 2-نہیں<br>a ۔ تمباکو نوشی کی صورت میں<br>b۔چبانے کی صورت میں<br>c۔ کسی بھی اور صورت میں (نسوار، ٹوتھ پیسٹ ، گل وغیرہ	
3	کیا آپ آجکل (پچھلے 6 مہینوں میں) تمباکو کا استعمال کرتے ہیں؟............................	1-ہاں<br>2۔ نہیں	(اگر جواب 2ہو تو سوال نمبر 5 پر جائی)
4	اگر ہاں تو کتنا استعمال کرتے ہیں؟............................	1۔ باقاعدگی سے (ہفتہ میں ایک یا ایک سے زیادہ مرتبہ)<br>2۔ کبھی کبھی (ہفتہ میں ایک سے کم)<br>3۔ نہیں<br>9۔لاگو نہیں ہوتا	تمباکو نوشی کی صورت میں<br>چبانے کی صورت میں<br>کسی اور صورت میں

5	تمباکو کے استعمال کی مقدار اور مدت۔<br>(موجودہ اور سابقہ تمباکو استعمال کرنے والے دونوں کیلئے)							
	تمباکو کی قسم	برانڈ کا نام		مدت استعمال		مہینے میں استعمال<br>*تعداد **دفعہ ***انداز گرام	اگر استعمال کرنا چھوڑ دیا ہو تو کتنے عرصے سے چھوڑا ہے	
				مہینے	سال		مہینے	سال
1	*سگریٹ							
2	*بیڑی							
3	*سگار							
4	**حقہ/چیلم/پائپ							
5	***چبانے والا تمباکو							
6	***پان تمباکو والا							
7	***پان مصالحہ							
8	**نسوار							
9	***گٹکا							
10	دیگر (دیگر کی وضاحت)							

6	آپ نے کس عمر سے باقاعدگی سے تمباکو نوشی شروع کی؟	اگر لاگو نہیں ہوتا تو (99) لکھیں	سال ☐☐
7	آپ نے کس عمر سے چبانے والی اشیاء، تمباکو والے پان، نسوار، گٹکا یا سٹی وغیرہ کا استعمال باقاعدگی سے شروع کیا؟..	اگر لاگو نہیں ہوتا تو (99) لکھیں	سال ☐☐
8	کیا آپ اکثر ایسے ماحول میں رہتے ہیں جہاں دوسرے لوگ تمباکو نوشی کرتے ہیں (مثلاً گھر میں، کام کی جگہ پر، سفر کے دوران یا کسی اور جگہ پر)*ہفتہ میں کم از کم ایک دفعہ	1-ہاں<br>2-نہیں	☐<br>(اگر جواب 2 ہو تو پارٹ B پر جائیں)
9	اگر ہاں تو	ہفتے میں کتنے دن ☐<br>دن میں کتنا وقت منٹ ☐☐ گھنٹے ☐☐	

PART-B

ALCOHOL USE

شراب کا استعمال

1	کیاآپ نے کبھی شراب استعمال کی ہے؟..	1-ہاں<br>2-نہیں	☐<br>اگر جواب 2ہو تو PART,C پر جائیں
2	آپ شراب والی مشروبات کا کتنا استعمال کرتے ہیں؟............................	1-آجکل باقاعدگی سے شراب استعمال کرتے ہیں<br>2-*آجکل کبھی کبھار شراب کا استعمال کرتے ہیں*(ہفتے میں ایک دفعہ سے کم)<br>3-ماضی میں شراب کا استعمال کرتے تھے(6ماہ سے زیادہ ہوئے ہیں)<br>4-حال ہی میں شراب کا استعمال چھوڑا ہے(6 ماہ سے کم ہوئے ہیں)<br>5- کبھی شراب کا استعمال نہیں کیا	☐<br>اگر جواب 5ہو تو PART,C پر جائیں

3 شراب کے استعمال کی تفصیل(موجودہ اور ماضی میں شراب استعمال کرنے والوں کیلئے)

	شراب کی اقسام	استعمال کا دورانیہ		ہفتے میں استعمال کی تعداد	مقدار*	اگر چھوڑ دی ہے تو کتنا عرصہ ہوا	
		سال	مہینے			سال	مہینے
a	دیسی شراب(کوپی، ٹھرا، کچی شراب)						
b	شراب						
c	بیئر						
d	وائین						

*متبادل مقدار:-ایک چھوٹا پیگ=30ml -ایک بڑا پیگ=60ml- ایک بہت بڑا پیگ=90ml-ایک گلاس بیئر=325ml ایک گلاس وائین=100ml (برائے مہربانی مقامی حساب سے مقدار کا تعین کریں)

PART-C

PHYSICAL ACTIVITY

جسمانی مشقت کے بارے میں

اب ہم جسمانی مشقت کے بارے میں جاننے کے لئے چند سوالات پوچھیں گے جو لوگوں کی روزمرہ زندگی کا حصہ ہیں- برائے مہربانی اگر آپ سمجھتے ہیں کہ آپ کوئی جسمانی مشقت نہیں کرتے تب بھی سارے سوالوں کے جواب دیں- اس جسمانی مشقت کے بارے میں سوچیں جو آپ کام کے دوران، گھر میں، ایک جگہ سے دوسری جگہ جاتے ہوئے اور اپنے فارغ اوقات میں مشغلہ کے طور پر کرتے ہیں یا کوئی ورزش یا کھیل جس میں آپ حصہ لیتے ہیں- اب آپ اس مشقت کے بارے میں سوچیں جو آپ معمول کے ایک ہفتے میں کرتے ہیں یہ وہ سخت مشقت ہے جس سے آپکی سانس بہت تیز ہو جاتی ہو جیسے بھاری سامان اٹھانا، کھدائی کرنا، ورزش کرنا، پیدل تیز چلنا یا سائیکل چلانا- صرف ان کاموں کے بارے میں سوچیں جو آپ کم از کم 10 منٹ کیلئے ایک وقت میں مسلسل کرتے ہوں-

1	معمول کے سات دنوں میں کتنے دن آپ سخت جسمانی مشقت کرتے ہیں؟........	1- دن فی ہفتہ (اگر 0 ہو تو سوال 3 پر جائیں)<br>2- بتانے سے انکار کردیا (سوال 3 پر جائیں)<br>3- پتہ نہیں/اندازہ نہیں (سوال 3 پر جائیں)<br>4- کوئی سخت جسمانی مشقت نہیں کرتے ⟶ (سوال 3 پر جائیں)		☐<br>☐☐
2	ان دنوں میں کسی ایک دن آپ کتنے وقت کیلئے سخت جسمانی مشقت کرتے ہیں صرف ان کاموں کے بارے میں سوچیں جو آپ کم از کم 10 منٹ تک ایک ہی وقت میں کرتے ہوں..	1- گھنٹہ/ منٹ فی دن<br>2- بتانے سے انکار کردیا (سوال نمبر 2a پر جائیں)<br>3- پتہ نہیں/اندازہ نہیں (سوال نمبر 2a پر جائیں)	گھنٹے<br>منٹ	☐<br>☐☐<br>☐☐

انٹرویو لینے والے کیلئے مزید معلومات

اگر روزانہ مختلف دورانیہ کیلئے سخت جسمانی مشقت کرنے کی وجہ سے جواب دینے والے (Respondent) کیلئے جواب دینا مشکل ہو تو کہیں، مجھے دلچسپی ہے کسی بھی ایک دن کے اوسط وقت سے جس میں آپ سخت جسمانی مشقت کرتے ہیں-

2a	کیا آپ مجھے بتا سکتے ہیں کے آپ معمول کے 7 دنوں میں کل کتنا وقت سخت جسمانی مشقت کرتے ہوئے گزارتے ہیں؟..............................	1- گھنٹہ/ منٹ فی دن<br>2- بتانے سے انکار کردیا<br>3- پتہ نہیں/اندازہ نہیں	گھنٹے<br>منٹ	☐<br>☐☐<br>☐☐

اب آپ اُن تمام درمیانی درجے کی مشقت والے کاموں کے بارے میں سوچیں جو آپ معمول کے 7 دنوں میں کرتے ہیں- درمیانی درجے کی جسمانی مشقت وہ ہے جس سے آپ معمول سے تھوڑی تیز اور گہری سانس لینے لگتے ہیں- ان میں پیدل چلنا شامل ہے- صرف اُن کاموں کے بارے میں سوچیں جو آپ کم از کم 10 منٹ کے لئے مسلسل (ایک وقت میں) کرتے ہیں

3	معمول کے 7 دنوں میں کتنے دن آپ درمیانی درجے کی جسمانی مشقت کم از کم 10 منٹ کرتے ہیں؟..............................<br>(مثال کے طور پر:- بھاری چیزیں اٹھانا، ایک خاص رفتار سے سائیکل چلانا، ٹینس کھیلنا، ہاتھ سے کپڑے دھونا، فرش پر پونچھا لگنا، باغبانی، 3 سال سے کم عمر کے بچوں کا خیال رکھنا، گاڑی یا موٹر سائیکل دھونا، بازار سے سبزیاں اور دیگر اشیاء اٹھا کر گھر تک پیدل چلنا، سیڑھیاں چڑھنا (3 یا زیادہ منزلیں) اور پتھر سے چٹنی پیسنا-	1- دن فی ہفتہ (اگر 0 ہو تو سوال 5 پر جائیں)<br>2- بتانے سے انکار کردیا (سوال 5 پر جائیں)<br>3- پتہ نہیں/اندازہ نہیں (سوال 5 پر جائیں)<br>4- کوئی درمیانی درجے کی جسمانی مشقت نہیں کرتے (سوال 5 پر جائیں)	☐<br>☐☐

4	ان دنوں میں کسی ایک دن آپ کتنے وقت کیلئے درمیانی درجے کی جسمانی مشقت کرتے ہیں صرف ان جسمانی مشقت کے بارے ہی سوچیں جو آپ کم از کم 10 منٹ تک ایک ہی وقت میں کرتے ہوں۔	1۔ گھنٹہ / منٹ فی دن<br>2۔ بتانے سے انکار کر دیا (سوال نمبر 4a پر جائیں)<br>3۔ پتہ نہیں / اندازہ نہیں (سوال نمبر 4a پر جائیں)	گھنٹے<br>منٹ	

انٹرویو لینے والے کیلئے مزید معلومات

اگر روزانہ مختلف دورانیہ کیلئے درمیانی درجے کی جسمانی مشقت کرنے کی وجہ سے جواب دینے والے (Respondent) کیلئے جواب دینا مشکل ہو تو کہیں، مجھے دلچسپی ہے کسی بھی ایک دن کے اوسط وقت سے جس میں آپ درمیانی درجے کی جسمانی مشقت کرتے ہیں۔

4a	معمول کے 7 دنوں میں کتنا وقت آپ درمیانی درجے کی جسمانی مشقت کرتے ہوئے گزارتے ہیں؟	1۔ گھنٹہ / منٹ فی دن<br>2۔ بتانے سے انکار کر دیا<br>3۔ پتہ نہیں / اندازہ نہیں	گھنٹے<br>منٹ	

اب آپ اس وقت کے بارے میں سوچیں جو آپ معمول کے 7 دنوں میں پیدل چلتے ہوئے گزارتے ہیں اس میں کام کے دوران، گھر میں اور ایک جگہ سے دوسری جگہ پیدل چل کے جانا شامل ہیں۔ اس میں وہ پیدل چلنا بھی شامل ہے جو آپ صرف تفریح کے لئے، کھیل میں یا ورزش کرتے ہوئے کرتے ہیں مثلاً بس اسٹاپ تک، کام کی جگہ تک، یا مارکیٹ تک کم از کم 10 منٹ تک پیدل چل کے جانا۔

5	معمول کے 7 دنوں ہی کتنے دن آپ کم از کم 10 منٹ تک ایک وقت میں پیدل چلتے ہیں۔ صرف اس پیدل چلنے کے بارے میں سوچیں جو کم از کم 10 منٹ تک ایک ہی وقت میں ہو۔	1۔ دن فی ہفتہ (اگر 0 ہو تو سوال 7 پر جائیں)<br>2۔ بتانے سے انکار کر دیا (سوال 7 پر جائیں)<br>3۔ پتہ نہیں / اندازہ نہیں (سوال 7 پر جائیں)<br>4۔ پیدل نہیں چلتے (سوال 7 پر جائیں)		
6	ان دنوں میں کسی ایک دن آپ عموماً کتنا وقت پیدل چلتے ہوئے گزارتے ہیں۔	1۔ گھنٹہ / منٹ فی دن (سوال نمبر 7 پر جائیں)<br>2۔ بتانے سے انکار کر دیا (سوال 6a نمبر پر جائیں)<br>3۔ پتہ نہیں / اندازہ نہیں (سوال نمبر 6a پر جائیں)	گھنٹے<br>منٹ	

انٹرویو لینے والے کیلئے مزید معلومات

اگر روزانہ مختلف دورانیہ کیلئے پیدل چلنے کی وجہ سے جواب دینے والے (Respondent) کیلئے جواب دینا مشکل ہو تو کہیں، مجھے دلچسپی ہے کسی بھی ایک دن کے اوسط وقت سے جو آپ ان دنوں میں سے کسی ایک دن پیدل چل کے گزارتے ہیں

6a	کیا آپ مجھے بتا سکتے ہیں کہ آپ معمول کے 7 دنوں میں کل کتنا وقت پیدل چلتے ہوئے گزارتے ہیں؟	1۔ گھنٹہ / منٹ فی دن<br>2۔ بتانے سے انکار کر دیا<br>3۔ پتہ نہیں / اندازہ نہیں	گھنٹے<br>منٹ	

اب آپ اس وقت کے بارے میں سوچیں جو آپ معمول کے 7 دنوں (چھٹی کے دنوں کے علاوہ) میں بیٹھ کر گزارتے ہیں اس میں وہ وقت شامل ہے جو آپ کام کے دوران، گھر میں یا تفریح کے دوران بیٹھ کر گزارتے ہیں اس میں وہ وقت بھی شامل ہے جو آپ دفتر میں بیٹھ کر کام کرتے ہوئے گزارتے ہیں، دوستوں کے ہاں بیٹھتے ہیں، پڑھتے ہوئے یا بیٹھ / لیٹ کر ٹی۔وی دیکھنا، بچوں کو پڑھاتے ہوئے، سبزیاں کاٹتے ہوئے، سلائی کڑھائی کرتے ہوئے، نماز پڑھتے ہوئے، فون پر بات کرتے ہوئے یا کمپیوٹر پر کام کرتے ہوئے گزارتے ہیں۔

7	معمول کے 7 دنوں میں عموماً کام کے دنوں میں کتنا وقت بیٹھ کر گزارتے ہیں؟<br>(رات کو سونے کا وقت شامل نہ کریں)	1۔ گھنٹہ / منٹ فی دن (سوال نمبر 8 پر جائیں)<br>2۔ بتانے سے انکار کر دیا (سوال نمبر 7a پر جائیں)<br>3۔ پتہ نہیں / اندازہ نہیں (سوال نمبر 7a پر جائیں)	گھنٹے<br>منٹ	

انٹرویو لینے والے کیلئے مزید معلومات اگر روزانہ مختلف دورانیہ کیلئے بیٹھ کر وقت گزارنے کی وجہ سے جواب دینے والے کیلئے جواب دینا مشکل ہو تو کہیں، مجھے دلچسپی ہے اس اوسط وقت سے جو آپ روز بیٹھ کر گزارتے ہیں ؟			
7a	کیا آپ مجھے بتا سکتے ہیں کہ آپ نے پچھلی بدھ کے دن کتنا وقت بیٹھ کر گزارا تھا.............	1۔گھنٹہ/ منٹ بدھ کے دن<br>2۔بتانے سے انکار کردیا<br>3۔پتہ نہیں/اندازہ نہیں	گھنٹے ☐☐<br>منٹ ☐☐
8	مزید تبصرہ:۔		

PART-D

SLEEP

نیند کے بارے میں

1	آپ کو رات کو سونے کیلئے عماً کتنے گھنٹے ملتے ہیں ؟.......................<br>(ہر رات سونے کے اوسط گھنٹے)	کام کے دنوں میں<br>چھٹی کے دنوں میں	گھنٹے ☐☐<br>گھنٹے ☐☐
2	عام طور پہ ایک ہفتہ میں آپ کتنی دفعہ 5منٹ یا اس سے زیادہ قیلولہ (دن کے وقت سونا) کرتے/کرتی ہیں؟<br>(اگر بتانے والا کوئی قیلولہ نہیں کرتا تو00لکھیں)		☐☐
3	برائے مہربانی بتائیں کہ آپ کو مندرجہ ذیل کیفیتوں کا تجربہ کتنی دفعہ ہوا ہے؟......<br>(نیچے دیئے گئے کوڈز کے مطابق بتائیں)<br>1۔ کبھی نہیں<br>2۔ بہت کم(مہینے میں ایک دفعہ یا اس سے کم)<br>3۔ بعض دفعہ (مہینے میں 2سے4مرتبہ)<br>4۔اکثر(مہینے میں5سے15مرتبہ)<br>5۔ تقریباً ہمیشہ(مہینے میں16سے30مرتبہ)		
A	نیند مشکل سے آتی ہے		☐
B	رات کو سوتے میں جاگ جاتے ہیں اور پھر دوبارہ مشکل سے نیند آتی ہے		☐
C	صبح بہت جلدی جاگ جاتے ہیں اور پھر نیند نہیں آتی		☐
D	آپ دن میں بے سکونی محسوس کرتے		☐
E	نیند ناکافی ہوتی ہے		☐
F	سونے کے لیے نیند کی گولیاں یا کوئی اور دوائی استعمال کرتے ہیں		☐
سوال نمبر 4سے10تک نیند میں خراٹے مارنے اور سانس لینے کے بارے میں ہیں۔ان سوالات کا جواب دیتے ہوئے وہ بتائیں جو آپ اپنے بارے میں جانتے ہیں یا کسی اور نے آپ کو بتایا ہو۔			
4	کیا آپ نے کبھی نیند میں خراٹے لیئے ہیں(ابھی یا کبھی ماضی میں)..........................	1۔ہاں<br>2۔ نہیں<br>8۔پتہ نہیں	☐<br>اگر جواب2یا8ہو تو سوال نمبر 7پر جائیں

5	آجکل آپ کس کثرت سے خراٹے لیتے ہیں؟	0-اب خراٹے نہیں لیتے<br>1-بہت کم (ہفتہ میں ایک رات سے کم)<br>2-کبھی کبھار (ہفتہ میں 1سے 2راتیں)<br>3-اکثر (ہفتہ میں 3سے 5راتیں)<br>4-ہمیشہ یا تقریبا ہمیشہ (ہفتہ میں 3سے 5راتیں)<br>8-پتہ نہیں	☐<br>اگر جواب 0ہو تو سوال نمبر 7پر جائیں
6	آپ کے خراٹوں کی آواز کتنی بلند ہے؟	1-گہری سانسوں سے کچھ زیادہ<br>2-تقریبا اتنی بلند جیسے باتیں کرنا<br>3-باتیں کرنے سے زیادہ<br>4-بہت زیادہ بلند (بند دروازے سے باہر سنی جاسکتی ہے)<br>8-پتہ نہیں	☐
7	جو آپ نے محسوس کیا ہو یا گھر والوں نے آپکو بتایا ہو اس کی بنیاد پر کیا ایسے مواقع آئے ہیں کہ آپ نیند میں سانس لینا بند کردیتے ہیں؟	1-ہاں<br>2-نہیں<br>8-پتہ نہیں	☐<br>اگر جواب 2یا 8ہو تو سوال نمبر 9پر جائیں)
8	ہفتے میں کتنی دفعہ آپکو ایسا محسوس ہوتا ہے جیسے نیند میں آپکی سانس رک گئی ہو؟	1-بہت کم (ہفتہ میں ایک رات سے کم)<br>2-کبھی کبھار (ہفتہ میں 1سے 2راتیں<br>3-اکثر (ہفتہ میں 3سے 5راتیں<br>4-ہمیشہ یا تقریبا ہمیشہ (ہفتہ میں 3سے 5راتیں<br>8-پتہ نہیں	☐
9	کیا آپکو کبھی کسی ڈاکٹر نے بتایا ہے کہ آپکو ایسی بیماری ہے جس میں نیند میں سانس رک جاتی ہے؟	1-ہاں<br>2-نہیں<br>8-پتہ نہیں	☐<br>(اگر جواب 1ہو تو سوال نمبر 11پر، اگر جواب 2یا 8ہو تو سوال نمبر 10پر جائیں)
10	کیا آپکو کبھی کسی ڈاکٹر نے بتایا ہے کہ آپکو نیند کی کوئی اور بیماری ہے؟	1-ہاں<br>2-نہیں<br>8-پتہ نہیں	☐<br>(اگر جواب 2یا 8ہو تو سوال نمبر 11پر جائیں)
10a	اگر اوپر والے سوال کا جواب ہاں ہے تو برائے مہربانی اس بیماری کی وضاحت کریں		
11	کیا آپ عموما نیند کے دوران آکسیجن تھراپی کرتے ہیں؟ ....<br>(ماسک یا ناک میں کینولا کے ذریعے آکسیجن فراہم ہونا)	1-ہاں<br>2-نہیں	☐
12	پچھلے ایک سال کے دوران کتنی دفعہ آپکے گھر کا کوئی فرد یا افراد اس کمرے کے قریب رہے ہیں جہاں آپ سوئے ہوئے ہوں؟	1-کبھی نہیں<br>2-کبھی کبھار<br>3-عموما	☐

13	کتنا امکان ہے کہ مندرجہ ذیل حالات میں آپکو نیند آجائے اور آپکی آنکھ لگ جائے؟ (صرف تھکاوٹ نہیں) مندرجہ ذیل کوڈز کے مطابق بتائیں۔<br>1۔ کوئی امکان نہیں 2۔ تھوڑا سا امکان ہے 3۔ پچاس فیصد امکان ہے 4۔ بہت زیادہ امکان ہے<br>(اگر آپ کبھی اسی حالت سے نہیں گزرے تو برائے مہربانی اپنے اندازے سے بتائیں)		
A	بیٹھ کر پڑھنا		☐
B	ٹی۔وی دیکھنا		☐
C	کسی جگہ پر آرام سے بیٹھنا (جیسے تھیٹر یا کس میٹنگ میں)		☐
D	گاڑی میں سواری کے طور پر ایک گھنٹے تک بغیر رکے سفر کرنا		☐
E	اگر حالات اجازت دیں تو دوپہر میں آرام کیلئے لیٹنا۔		☐
F	بیٹھ کر کسی سے باتیں کرنا۔		☐
G	دوپہر کے کھانے کے بعد خاموشی سے بیٹھنا۔		☐
H	گاڑی میں، جب گاڑی چند منٹ کیلئے ٹریفک میں رکی ہو۔		☐
I	رات کے کھانے کے وقت کھانے کی میز پر		☐
J	گاڑی چلاتے ہوئے		☐
14	کیا آپ کبھی ایسپرین یا ایسی دوائی جس میں ایسپرین ہو استعمال کرتے ہیں؟.........	1۔ کبھی نہیں<br>2۔ ہفتے میں ایک دفعہ سے کم<br>3۔ ہفتے میں ایک یا دو دفعہ<br>4۔ ہر دوسرے دن<br>5۔ روزانہ<br>8۔ پتہ نہیں<br>9۔ ماضی میں استعمال کرتے تھے (پچھلے 12 مہینوں میں)	☐
15	کیا آپ گاڑی چلاتے ہیں؟	1۔ ہاں<br>2۔ نہیں	☐
16	اگر اوپر والے سوال کا جواب ہاں ہے تو مندرجہ ذیل سوالات پوچھیں ورنہ پارٹ E پر جائیں۔		
A	کتنے سالوں سے آپ گاڑی چلا رہے ہیں؟		☐☐
B	کتنے وقت کے لئے آپ گاڑی چلاتے ہیں؟	1۔ روزانہ<br>2۔ کبھی کبھار<br>3۔ بہت کم / کبھی نہیں	☐
C	جب سے آپ گاڑی چلانا شروع کی ہے تب سے اب تک آپ گاڑی چلاتے ہوئے کتنے حادثات کا شکار ہوئے ہیں؟		☐☐
D	پچھلے ایک سال کے دوران گاڑی چلاتے ہوئے کتنے حادثات کا شکار ہوئے ہیں؟		☐☐

PART-E

DIET

غذا/خوراک کے بارے میں

1	کیا آپ صرف سبزیاں کھاتے ہیں؟(کسی قسم کا گوشت یا انڈے بالکل نہیں کھاتے)	1-ہاں<br>2-نہیں	☐
2	کیا آپ انڈے کھاتے ہیں؟..	1-ہاں<br>2-نہیں	☐
3	کیا آپ کسی خاص قسم کی غذا کھاتے ہیں؟(مثلاً پرہیزی کھانا)	1-ہاں<br>2-نہیں	☐<br>اگر جواب 2 ہو سوال نمبر 6 پر جائیں
4	اگر ہاں تو آجکل آپ کس قسم کی غذا کھا رہے ہیں؟................................	1-ہاں 2-نہیں<br>شوگر/ذیابیطس کی مخصوص غذا<br>کم چربی/ کم چکنائی والی غذا<br>زیادہ فائبر والی غذا<br>کم نمک والی غذا<br>وزن کم کرنے والی غذا<br>دیگر	☐<br>☐<br>☐<br>☐<br>☐<br>☐<br>دیگر کی وضاحت کریں----------
5	کتنے عرصہ سے آپ یہ (مندرجہ بالا) مخصوص غذا کھا رہے ہیں؟..............................	زیادہ سے زیادہ عرصہ لکھیں	☐☐ ☐☐<br>مہینے سال
6	آپ کتنی دفعہ استعمال شدہ تیل کو دوبارہ استعمال کرتے ہیں؟........................	1-روزانہ<br>2-ہر دوسرے دن<br>3-ہفتہ میں 1 یا 2 مرتبہ<br>4-ہفتہ میں 1 دفعہ سے کم<br>5-کبھی نہیں	☐

5-پچھلے ایک سال کے دوران آپ نے کھانے پینے کی مندرجہ ذیل اشیاء کا استعمال کس حساب سے کیا ہے

نمبر شمار	کھانے پینے کی اشیاء	کبھی نہیں کھایا یا (✓) مہینے میں ایک دفعہ سے کم	مہینے میں ایک دفعہ	ہفتے میں ایک دفعہ	روزانہ
1	گوشت (بکرے، دنبے، گائے، بھینس، خرگوش کا گوشت اور ان کا سالن)				
2	پولٹری (مرغی، بطخ، بیٹر اور ان کا سالن)				
3	مغز، نہاری، پائے وغیرہ (جگر، گردے، مغز، دل، نہاری، پائے)				
4	مچھلی (میٹھے پانی اور سمندری پانی کی، ڈبے والی نمکین، سوکھی ہوئی مچھلی)				

نمبر شمار	کھانے پینے کی اشیاء	کبھی نہیں کھایا یا (✓) مہینے میں ایک دفعہ سے کم	مہینے میں ایک دفعہ	ہفتے میں ایک دفعہ	روزانہ
5	جھینگا، کیکڑا وغیرہ (جھینگا، کیکڑا، مچھلی کے انڈے)				
6	انڈے (مرغی اور بطخ کے انڈے)				
7	دودھ اور دودھ سے بنی ہوئی اشیاء (دودھ، دہی، پنیر، ،لسی، رائتہ، دودھ والی مشروبات)				
8	دودھ سے بنی ہوئی میٹھی غذا (کسٹرڈ، کھویا، فرنی، کھیر، پڈنگ، رس گلہ، رس ملائی، آئس کریم، اور دیگر)				
9	تلی ہوئی اشیاء مغربی طرز کے کھانے (فرنچ فرائز، چپس، آلو کے چپس، پیاز، چکن نگٹس)				
10	تلی ہوئی اشیاء دیسی طرز کے کھانے (سموسے، پاپڑ، پکوڑے، نمک پارے، انڈے والے رول، پوری، کچوری)				
11	مغربی طرز کے میٹھے کھانے (کیک، پیسٹری، چاکلیٹ، ٹافیاں، بسکٹ)				
12	مٹھائی (برفی، لڈو، گلاب جامن، حلوہ)				
13	ٹھنڈے مشروبات (گیس والی مشروبات، شربت)				
14	پھل(1) (سٹرابری، پائن ایپل، جامن، سیب)				
15	پھل(2) (اوپر دیئے گئے پھلوں کے سوا تمام موسمی پھل)				
16	پھلوں کا رس / جوس (ہر قسم کے، گھر میں بنے، بازار سے خریدے گئے، تازہ اور جمے ہوئے)				
17	ڈرائی فروٹ (مونگ پھلی، بادام، پستہ، اخروٹ، سورج مکھی کے بیج)				
18	سبز پتے والی سبزیاں (تمام تازہ سبز پتے والی سبزیاں ساگ، پالک، شلجم)				
19	دیگر کچی سبزیاں (ہر قسم کی کچی سبزیاں جو اوپر دیئے گروپ میں شامل نہیں)				
20	دالیں (تمام دالیں، چنا، چھولے)				
21	اچار اور اچار والے کھانے (اچار، چٹنی)				
22	دیگر پکی ہوئی سبزیاں (تمام پکی سبزیاں جو اوپر دیئے گروپ میں شامل نہیں)				
23	چاول اور کم فائبر والی دیگر اشیاء (ابلے چاول، تلے ہوئے چاول، بریانی، پلاؤ ،ادلی، ڈوسہ، سوجی، ساگو دانہ، پاستا، شیرمال، تافتان، نان)				
24	روٹی اور دیگر مکمل اناج (بھوسی والے آٹے کی روٹی، رس، دلیہ، براون بریڈ)				
25	چائے (قہوہ اور دوسرے اقسام کی چائے)				
26	کافی (دودھ اور بلیک)				

SECTION-3

FEMALE REPRODUCTIVE HISTORY

خواتین کی تولیدی معلومات

(یہ سیکشن صرف خواتین سے پوچھا جائے گا- مردوں کیلئے یہ سیکشن چھوڑ کر سیکشن 4 سے پوچھیں)

1	آپ کے اب تک کتنے حمل ہوئے ہیں؟..		
2	کس عمر میں آپکو ماہواری آنا شروع ہوئی؟..		
3	آج کل آپ کو ماہواری آتی ہے؟	1-ہاں<br>2-نہیں	اگر جواب 1 ہو تو سوال نمبر 6 پر جائیں
4	اگر نہیں تو کیا وجہ ہے؟	1-حمل سے ہیں<br>2-بچے کو دودھ پلاتی ہیں<br>3-ماہواری آنا بند ہو گئی ہے<br>4-آپریشن کے ذریعے ماہواری بند کروائی ہے<br>5-دیگر وجوہات	(دیگر کی وضاحت کریں)<br>....................................
5	اگر ماہواری آنا بند ہو گی ہے تو کتنے عرصے سے؟..		سال<br>مہینے
6	حمل روکنے والی کوئی گولیاں یا دوائی استعمال کرتی ہیں؟		
	ماضی میں کبھی استعمال کی<br>اگر ہاں تو کتنے عرصہ تک	1-ہاں 2-نہیں	سال<br>مہینے
	آجکل استعمال کرتی ہیں؟..	1-ہاں 2-نہیں	
	اگر ہاں تو کتنے عرصہ تک		سال<br>مہینے

SECTION-4

QUALITY OF LIFE

معیارزندگی

برائے مہربانی نیچے دیئے گئے کوڈز کے مطابق ان جملوں کی نشاندہی کیجئے جو آپکی موجودہ صحت کی بہترین عکاسی کرتے ہیں۔

1	چلنا پھرنا	1۔مجھے چلنے پھرنے میں کوئی مشکل نہیں<br>2۔مجھے چلنے پھرنے میں کوئی مشکل ہوتی ہے<br>3۔میں بالکل چل پھر نہیں سکتا/سکتی	☐
2	اپنی دیکھ بھال خود کرنا	1۔مجھے اپنی دیکھ بھال خود کرنے میں کوئی مشکل نہیں<br>2۔مجھے خود نہانے دھونے اور کپڑے پہننے میں کچھ مشکل ہوتی ہے<br>3۔میں خود نہا دھو اور کپڑے پہن نہیں سکتا/سکتی	☐
3	روزمرہ کی معملات<br>(مثلاً کام، پڑھائی، گھریلو کام کاج، خاندانی اور تفریحی مصروفیات)	1۔مجھے اپنے روزمرہ کے کام کاج میں کوئی مشکل نہیں ہوتی<br>2۔مجھے اپنے روزمرہ کے کام کاج میں کچھ مشکل ہوتی ہے<br>3۔میں اپنے روزمرہ کے کام کاج نہیں کر سکتا/سکتی	☐
4	درد/پریشانی	1۔مجھے کوئی درد یا پریشانی نہیں<br>2۔مجھے کچھ درد یا پریشانی ہے<br>3۔مجھے شدید درد یا پریشانی ہے	☐
5	تشویش/ مایوسی	1۔مجھے کوئی تشویش یا مایوسی نہیں ہے<br>2۔مجھے کچھ تشویش یا مایوسی ہے<br>3۔مجھے شدید تشویش یا مایوسی ہے	☐

آپکی اچھی یا خراب صحت کا اندازہ کرنے کیلئے آپکی سہولت کیلئے ہم نے ایک پیمانہ (تھرمامیٹر) کی طرح بنایا ہے اس پیمانے پہ سو(100) آپکی بہترین اور صفر(0) آپکی بد ترین صحت کی نشاندہی کرتا ہے ہم چاہتے ہیں کہ آپ اس پیمانے پر نشاندہی کریں کہ آج کل آپ کے خیال میں آپکی صحت کیسی ہے (بہترین یا بد ترین)۔ نیچے دیئے گئے خانے سے ایک لائن لگائیں جو پیمانے پر موجود اس نشان تک ہو جو آپکی موجودہ صحت کی صیح نشاندہی کرتی ہو۔

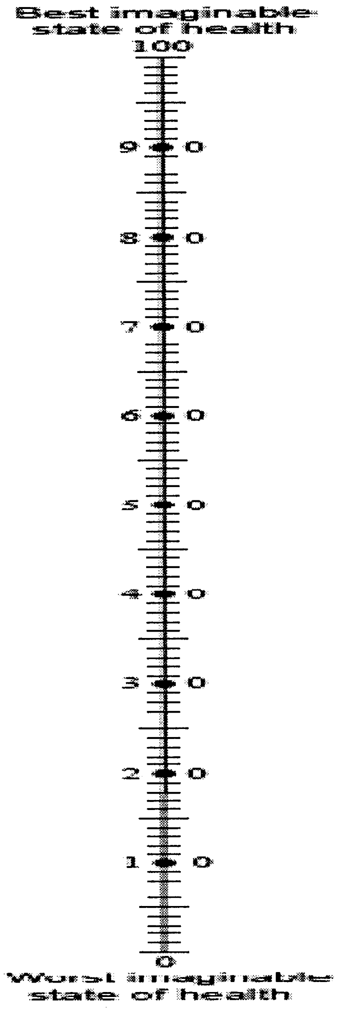

آپکی موجودہ صحت

SECTION-5

MEDICAL HISTORY

طبی معلومات

PART-A

CARDIOMETABOLIC DISEASES AND THEIR RISK FACTORS

امراض قلب اور ان کا سبب بننے والے عوامل

1	کیاآپکو کبھی کسی معالج نے بتایا ہے کہ آپکو مندرجہ ذیل میں سے کوئی بیماری / بیماریاں ہیں؟(دوران حمل ہونے والے بلڈ پریشر اور شوگر کو شامل نہ کریں)		
		1.ہاں 2.نہیں 3.پتہ نہیں<br>بلڈ پریشر<br>شوگر/ذیابیطس<br>کولسٹرول کی زیادتی<br>دل کی بیماری<br>فالج<br>گردوں کی دائمی/Chronic بیماری	☐<br>☐<br>☐<br>☐<br>☐<br>☐

اگر مندرجہ بالا سوال میں کسی کا جواب ,ہاں, ہے توPART-B پر جائیں ورنہ پوراPART-B چھوڑ کرPART-C پر جائیں۔

PART-B

DISEASE SPECIFIC QUESTIONS

بیماریوں کے متعلق مخصوص سوالات

I- HIGH BLOOD PRESSURE

بلڈ پریشر

(اگرPART-A کے سوال نمبر1 میں بلڈ پریشر کیلئے جواب ہاں ہے تو یہ سیکشن پوچھیں)

a	آپکو کتنے عرصے سے بلڈ پریشر کی بیماری ہے؟..		☐☐<br>☐☐
b	اس کیلئے آپ فی الحال کیا علاج کروا رہے ہیں؟...<br>*(ان میں یوگا، یونانی، ہومیوپیتھک، حکیمی اور جڑی بوٹیوں وغیرہ سے علاج شامل ہے)	1.ہاں 2.نہیں<br>خوراک میں تجویز کردہ تبدیلی<br>تجویز کردہ ورزش<br>*یونانی، حکیمی یا دیسی علاج<br>انگریزی دوائیاں<br>کچھ نہیں	☐<br>☐<br>☐<br>☐<br>☐

c	آپ یہ علاج کتنی پابندی سے کرتے ہیں؟	1- پابندی کے ساتھ<br>2- کبھی کبھار بھول جاتے ہیں<br>3- جب بلڈ پریشر بڑھا ہوا محسوس ہوتا ہے تو دوائی لیتے ہیں<br>4- ایک ماہ سے زیادہ کیلئے چھوڑ دیتے ہیں<br>5- کبھی دوا استعمال نہیں کی	☐<br>اگر جواب 4 ہے تو سوال نمبر d کا جواب دیں ورنہ سوال نمبر e پر جائیں
d	دوا/علاج چھوڑ دینے کی کیا وجہ ہے؟	1- خرید نہیں سکتے<br>2- برداشت نہیں کر سکتے<br>3- طبیعت صحیح ہو گئی ہے<br>4- کوئی وجہ نہیں<br>5- یاد نہیں<br>6- دیگر	☐<br>دیگر کی وضاحت کریں<br>..
e	کیا آپ کے خیال میں آپ کا بلڈ پریشر پوری طرح قابو میں ہے؟	1- ہاں<br>2- نہیں<br>3- پتہ نہیں	☐
f	کیا آپ کے معالج آپ سے کہتے ہیں کہ آپ کا بلڈ پریشر پوری طرح قابو میں ہے؟	1- ہاں<br>2- نہیں<br>3- پتہ نہیں	☐
g	آخری مرتبہ ڈاکٹر سے چیک اپ کروانے پر آپ کا بلد پریشر کتنا تھا؟	1- کتنا<br>2- پتہ نہیں<br>3- یاد نہیں	Systolic..............<br>Diastolic............<br>☐
h	آپ نے آخری مرتبہ معالج سے کب رجوع کیا تھا؟	1- 1ماہ سے کم<br>2- 1ماہ سے زیادہ<br>3- 3ماہ سے زیادہ<br>4- 6ماہ سے کم<br>5- 6ماہ سے زیادہ	☐
i	کیا آپکے پاس بلڈ پریشر سے متعلق طبی ریکارڈ یا نسخے ہیں؟	1-- ہاں<br>2- نہیں<br>3- پتہ نہیں	☐
j	اگر جواب ہاں ہے تو جواب دہندہ سے طبی ریکارڈ یا نسخے دیکھانے کیلئے کہیں اور تشخیص نوٹ کریں۔	تشخیص:-	
k	حالیہ نسخے میں لکھی گئی بلڈ پریشر کو نوٹ کریں۔		Systolic..............<br>Diastolic............

II- DIABETES

شوگر/ذیابیطس

(اگر PART-A کے سوال نمبر 1 میں شوگر کیلئے جواب ہاں ہے تو یہ سیکشن پوچھیں)

a	آپکو کتنے عرصے سے شوگر/ذیابیطس کی بیماری ہے		مہینے<br>سال
b	اس کیلئے آپ فی الحال کیا علاج کروارہے ہیں<br>*(ان میں یوگا، یونانی، ہومیوپیتھک، حکیمی اور جڑی بوٹیوں وغیرہ سے علاج شامل ہے)<br>1-ہاں 2-نہیں	خوراک میں تجویز کردہ تبدیلی<br>تجویز کردہ ورزش<br>*یونانی، حکیمی یا دیسی علاج<br>انگریزی دوائیاں<br>کچھ نہیں	
c	آپ یہ دوائیاں کتنی پابندی سے لیتے ہیں؟..	1-پابندی کے ساتھ<br>2-کبھی کبھار بھول جاتے ہیں<br>3-جب شوگر بڑی ہوئی ہے تو دوائی لیتے ہیں<br>4-ایک ماہ سے زیادہ کیلئے چھوڑ دیتے ہیں<br>5-کبھی دوا استعمال نہیں کی	اگر جواب 4 ہے تو سوال نمبر d کا جواب دیں ورنہ سوال نمبر e پر جائیں)
d	علاج/دوا چھوڑ دینے کی کیا وجہ ہے؟..	1-خرید نہیں سکتے<br>2-برداشت نہیں کرسکتے<br>3-طبیعت صحیح ہو گئی ہے<br>4-کوئی وجہ نہیں<br>5-یاد نہیں<br>6-دیگر	دیگر کی وضاحت کریں<br>..
e	کیا آپ کے خیال میں آپکی شوگر/ذیابیطس صحیح طرح قابو میں ہے؟..........................	1-ہاں<br>2-نہیں<br>3-پتہ نہیں	
f	کیا آپ کے معالج آپ سے کہتے ہیں کہ آپ کی شوگر/ذیابیطس پوری طرح قابو میں ہے؟	1-ہاں<br>2-نہیں<br>3-پتہ نہیں	
g	آخری مرتبہ معائنہ کرنے پر آپ کی نہار منہ اور کھانے کے بعد کی شوگر کتنی تھی؟		نہار منہ----------<br>کھانے کے بعد-----
h	آپنے آخری مرتبہ معالج سے کب رجوع کیا تھا؟....................................	1-1ماہ سے کم<br>2-1ماہ سے زیادہ<br>3-3ماہ سے زیادہ<br>4-6ماہ سے کم<br>5-6ماہ سے زیادہ	

i	کیاآپکے پاس شوگر/ذیابیطس سے متعلق طبی ریکارڈ یا نسخے ہیں؟........................	1-ہاں<br>2-نہیں<br>3-پتہ نہیں	☐
j	اگر جواب ہاں ہے تو جواب دہندہ سے طبی ریکارڈ یا نسخے دیکھانے کیلئے کہیں اور تشخیص نوٹ کریں-	تشخیص:-	
k	حالیہ نسخے میں لکھی گئی نہار منہ اور کھانے کے بعد کی شوگر نوٹ کریں		نہار منہ----------<br>کھانے کے بعد-----
Part-D بھی مکمل کریں-			

III- HIGH BLOOD CHOLESTEROL

خون میں چکنائی

(اگر PART-A کے سوال نمبر 1 میں کولیسٹرول کی زیادتی کیلئے جواب ہاں ہے تو یہ سیکشن پوچھیں)

a	آپکو کتنے عرصے سے کولیسٹرول کی زیادتی کی بیماری ہے؟............................		مہینے ☐☐<br>سال ☐☐
b	اس کیلئے آپ فی الحال کیاعلاج کروارہے ہیں؟.............................<br>*(ان میں یوگا، یونانی، ہومیوپیتھک، حکیمی اور جڑی بوٹیوں وغیرہ سے علاج شامل ہے)	1.ہاں 2.نہیں<br>خوراک میں تجویز کردہ تبدیلی<br>تجویز کردہ ورزش<br>*یونانی، حکیمی یا دیسی علاج<br>انگریزی دوائیاں<br>کچھ نہیں	☐<br>☐<br>☐<br>☐<br>☐
c	آپ یہ دوائیاں کتنی پابندی سے لیتے ہیں؟..............................	1-پابندی کے ساتھ<br>2-کبھی کبھار بھول جاتے ہیں<br>3-جب کولیسٹرول بڑھا ہوا ہوتا ہے<br>4-ایک ماہ سے زیادہ کیلئے چھوڑ دیتے ہیں<br>5-کبھی دوا استعمال نہیں کی	☐<br>(اگر جواب 4 ہے تو سوال نمبر d کا جواب دیں ورنہ سوال نمبر e پر جائیں)
d	دوا چھوڑ دینے کی کیا وجہ ہے؟..	1-خرید نہیں سکتے<br>2-برداشت نہیں کر سکتے<br>3-طبیعت صحیح ہو گئی ہے<br>4-کوئی وجہ نہیں<br>5-یاد نہیں<br>6-دیگر	☐<br>(دیگر کی وضاحت کریں)<br>....................................
e	کیاآپ کے خیال میں آپکا کولیسٹرول صحیح طرح قابو میں ہے؟..........................	1-ہاں<br>2-نہیں<br>3-پتہ نہیں	☐

f	کیاآپ کے معالج آپ سے کہتے ہیں کہ آپ کا کولیسٹرول پوری طرح قابو میں ہے؟....	1-ہاں<br>2-نہیں<br>3-پتہ نہیں	☐
g	آخری مرتبہ چیک کرنے پر آپ کے کولیسٹرول کی مقدار کتنی تھی؟................		-----------mg/dl
h	آپنے آخری مرتبہ معالج سے کب رجوع کیا تھا؟..........................	1-1ماہ سے کم<br>2-1ماہ سے زیادہ<br>3-3ماہ سے زیادہ<br>4-6ماہ سے کم<br>5-6ماہ سے زیادہ	☐
i	کیاآپکے پاس کولیسٹرول کی زیادتی سے متعلق طبی ریکارڈ یانسخے ہیں؟................	1-ہاں<br>2-نہیں<br>3-پتہ نہیں	☐
j	اگر جواب ہاں ہے تو جواب دہندہ سے طبی ریکارڈ یانسخے دیکھانے کیلئے کہیں اور تشخیص نوٹ کریں	تشخیص:-	
k	حالیہ نسخے میں لکھی گئی کولیسٹرول کی مقدار نوٹ کریں		-----------mg/dl

IV-HEART DISEASE

دل کی بیماری

(اگر part-A کے سوال نمبر 1 میں دل کی بیماری کیلئے جواب ہاں ہے تو یہ سیکشن پوچھیں)

A	آپکو پہلی مرتبہ کب معلوم ہوا کہ آپکو دل کی بیماری ہے؟..................	1-1سال سے کم ہوا ہے<br>2-1سے5سال ہوئے ہیں<br>3-5سال سے زیادہ ہوئے ہیں	☐
b	اپکے معالج نے آپکو کیا بتایا تھا کہ یہ کونسی بیماری ہے؟..................<br>(ایک سے زائد جوابات آسکتے ہیں)	1-دل کا دورہ<br>2-انجائنا<br>3-دل کی کمزوری/ہارٹ فیل ہونا<br>4-دل کے والو بند ہیں<br>5-دل میں سوراخ<br>6-دیگر<br>7-معالج نے نہیں بتایا	☐<br>☐<br>☐<br>(دیگر کی وضاحت کریں)<br>..............................<br>اگر جواب 1ہو تو سوال نمبر c جائیں ورنہ g پر جائیں
c	کس عمر میں آپکو دل کا پہلا دورہ پڑا؟....................................		☐☐ سال
d	کیاآپ علاج کیلئے ہسپتال میں داخل رہے؟..............................	1-ہاں<br>2-نہیں	☐

e	کیاآپکو دوبارہ دل کے دورے پڑے؟...	1-ہاں<br>2-نہیں	
f	کیا آپ دوسرے دورے کے علاج کیلئے ہسپتال میں داخل رہے؟...........................	1-ہاں<br>2-نہیں	
g	دل کی بیماری کیلئے آپ فی الحال کیا علاج کر رہے ہیں؟...................<br>*(ان میں یوگا، یونانی، ہومیوپیتھک، حکیمی اور جڑی بوٹیوں وغیرہ سے علاج شامل ہے)	**1-ہاں 2-نہیں**<br>خوراک میں تجویز کردہ تبدیلی<br>تجویز کردہ ورزش<br>*یونانی، حکیمی یا دیسی علاج<br>انگریزی دوائیاں<br>کچھ نہیں	
h	آپ یہ دوائیاں کتنی پابندی سے لیتے ہیں؟.....................................	1-پابندی کے ساتھ<br>2-کبھی کبھار بھول جاتے ہیں<br>3-جب طبیعت ٹھیک نہ ہو<br>4-ایک ماہ سے زیادہ کیلئے چھوڑ دیتے ہیں<br>5-کبھی دوا استعمال نہیں کی	اگر جواب 4 ہے تو سوال نمبر i کا جواب دیں ورنہ سوال نمبر j پر جائیں)
i	دوا چھوڑ دینے کی کیا وجہ ہے؟...	1-خرید نہیں سکتے<br>2-برداشت نہیں کر سکتے<br>3-طبیعت صحیح ہو گئی ہے<br>4-کوئی وجہ نہیں<br>5-یاد نہیں<br>6-دیگر	(دیگر کی وضاحت کریں)<br>..
j	آپ نے آخری مرتبہ معالج سے کب رجوع کیا تھا؟.....................................	1-1ماہ سے کم<br>2-1ماہ سے زیادہ<br>3-3ماہ سے زیادہ<br>4-6ماہ سے کم<br>5-6ماہ سے زیادہ	
k	کیا آپکے پاس دل کی بیماری سے متعلق طبی ریکارڈ یا نسخے موجود ہیں؟.................	1-ہاں<br>2-نہیں<br>3-پتہ نہیں	
l	اگر جواب ہاں ہے تو جواب دہندہ سے طبی ریکارڈ یا نسخے دیکھانے کیلئے کہیں اور تشخیص نوٹ کریں	تشخیص:-	

V- STROKE

فالج

(اگر PART-A کے سوال نمبر 1 میں فالج کیلئے جواب ہاں ہے تو یہ سیکشن پوچھیں)

a	جب آپکو فالج کا اثر ہوا تو اس وقت آپکی کیا عمر تھی؟........................		سال ☐☐
b	کیا جسم کے کسی حصہ میں معذوری باقی ہے؟..........................	1۔ہاں<br>2۔نہیں	☐
c	اگر ہاں تو کونسا حصہ متاثر ہے؟..	1۔ہاں 2۔نہیں<br>ٹانگ/پاوں کا فالج<br>ہاتھ/بازو کا فالج<br>ٹانگ/پاوں کی کمزوری<br>ہاتھ/بازو کی کمزوری<br>گفتگو کی خرابی<br>نظر کی خرابی<br>پیشاب کو کنٹرول نہ کرپانا<br>ان کے علاوہ جسم کے کسی دیگر حصے کی کمزوری (وضاحت کریں)	☐<br>☐<br>☐<br>☐<br>☐<br>☐<br>☐<br>☐<br>..
d	کیا فالج کے اثر کے بعد آپکو کسی دوا کو جاری رکھنے کا مشورہ دیا گیا؟....................	1۔ہاں<br>2۔نہیں	☐
e	اگر ہاں تو آپ یہ دوائیاں کتنی پابندی سے لیتے ہیں؟..............................	1۔پابندی کے ساتھ<br>2۔کبھی کبھار بھول جاتے ہیں<br>3۔جب طبیعت خراب ہو تب لیتے ہے<br>4۔ایک ماہ سے زیادہ کیلئے چھوڑ دیتے ہیں<br>5۔کبھی دوا استعمال نہیں کی	☐<br>اگر جواب 4 ہے تو سوال نمبر f کا جواب دیں ورنہ سوال نمبر g پر جائیں)
f	دوا چھوڑ دینے کی کیا وجہ ہے؟..	1۔خرید نہیں سکتے<br>2۔برداشت نہیں کرسکتے<br>3۔طبیعت صحیح ہو گئی ہے<br>4۔کوئی وجہ نہیں<br>5۔یاد نہیں<br>6۔دیگر	☐<br>(دیگر کی وضاحت کریں)<br>..

g	آپنے آخری مرتبہ معالج سے کب رجوع کیا تھا؟..............................	1۔1ماہ سے کم<br>2۔1ماہ سے زیادہ<br>3۔3ماہ سے زیادہ<br>4۔6ماہ سے کم<br>5۔6ماہ سے زیادہ	☐
h	کیا آپکے پاس فالج سے متعلق طبی ریکارڈ یا نسخے موجود ہیں؟..............................	1۔ہاں<br>2۔نہیں<br>3۔پتہ نہیں	☐
i	اگر جواب ہاں ہے تو جواب دہندہ سے طبی ریکارڈ یا نسخے دیکھانے کیلئے کہیں اور تشخیص نوٹ کریں	تشخیص:-	

VI- KIDNEY DISEASE

گردوں کی دائمی (Chronic) بیماری

(اگر PART-A کے سوال نمبر 1میں گردوں کی دائمی بیماری کیلئے جواب ہاں ہے تو یہ سیکشن پوچھیں)

a	کس عمر میں آپکو گردوں کی دائمی بیماری کی تشخیص ہوئی؟........................		سال ☐☐
b	گردوں کی دائمی بیماری کیلئے آپ فی الحال کیا علاج کررہے ہیں؟..................<br>*(ان میں یوگا، یونانی، ہومیوپیتھک، حکیمی اور جڑی بوٹیوں وغیرہ سے علاج شامل ہے)	1۔ہاں 2۔نہیں<br>خوراک میں تجویز کردہ تبدیلی<br>تجویز کردہ ورزش<br>*یونانی، حکیمی یا دیسی علاج<br>انگریزی دوائیاں<br>کچھ نہیں	☐<br>☐<br>☐<br>☐<br>☐
c	یہ علاج آپ کتنی باقاعدگی سے کررہے ہیں؟..............................	1۔پابندی کے ساتھ<br>2۔کبھی کبھار بھول جاتے ہیں<br>3۔جب طبیعت خراب ہو تب لیتے ہے<br>4۔ایک ماہ سے زیادہ کیلئے چھوڑ دیتے ہیں<br>5۔کبھی دوا استعمال نہیں کی	☐<br>اگر جواب 4ہے تو سوال نمبرdکا جواب دیں ورنہ سوال نمبرeپر جائیں)
d	دوا چھوڑ دینے کی کیا وجہ ہے؟..............................	1۔خرید نہیں سکتے<br>2۔برداشت نہیں کرسکتے<br>3۔طبیعت صیح ہو گئی ہے<br>4۔کوئی وجہ نہیں<br>5۔یاد نہیں<br>6۔دیگر	☐<br>(دیگر کی وضاحت کریں)<br>..............................

e	آپنے آخری مرتبہ معالج سے کب رجوع کیا تھا؟...	1-1ماہ سے کم<br>2-1ماہ سے زیادہ<br>3-3ماہ سے زیادہ<br>4-6ماہ سے کم<br>5-6ماہ سے زیادہ	☐
f	کیا آپکے پاس گردوں کی دائمی بیماری سے متعلق طبی ریکارڈ یا نسخے موجود ہیں؟.......	1-ہاں<br>2-نہیں<br>3-پتہ نہیں	☐
g	اگر جواب ہاں ہے تو جواب دہندہ سے طبی ریکارڈ یا نسخے دیکھانے کیلئے کہیں اور تشخیص نوٹ کریں	تشخیص:-	

PART-C

ANGINA, PERIPHERAL VASCULAR DISEASE AND HEART FAILURE

انجائنا، رگوں کی بیماری اور دل کی خرابیاں (ہارٹ فیل)

I- ANGINA

انجائنا

a	آپکو ان میں سے کوئی علامت موجود ہے؟..		
		1.ہاں 2.نہیں<br>دل کی دھڑکن محسوس کرنا<br>سینے میں درد<br>سانس کا پھولنا<br>تھکن /کمزوری<br>سینے میں دباو/بھاری پن /بے چینی	☐<br>☐<br>☐<br>☐<br>☐
	اگر سب کے لئے جواب 2 ہو سوال نمبر m پر جائیں		
b	کیا *مشقت کے دوران آپکو مندرجہ ذیل کوئی علامت سینے کے آس پاس، بازو، کاندھے، گردن، نچلے جبڑے میں، پیٹ یا کمر کے اوپر کے حصے میں محسوس ہوئی- *تیز چلنا، سیڑھیاں چڑھنا، وزن اٹھانا وغیرہ		
	اگر سب کے لئے جواب 2 ہو سوال نمبر d پر جائیں	1.ہاں 2.نہیں<br>درد<br>بوجھل پن / بھاری پن<br>دباو<br>بے چینی<br>بے حسی / سن ہونا	☐<br>☐<br>☐<br>☐<br>☐

c علامات زیادہ تر کس حصے میں محسوس ہوتی ہیں (برائے مہربانی جگہ کا تعین نیچے دی گئی تصویر سے شناخت کریں)
اضافی نمبر سینے کے پیچھے-11 گردن کے پیچھے-12

	درد<br>بوجھل پن / بھاری پن<br>بے چینی<br>بے حسی / سن ہونا	1-ہاں 2-نہیں<br>علامت	جگہ

Right Left

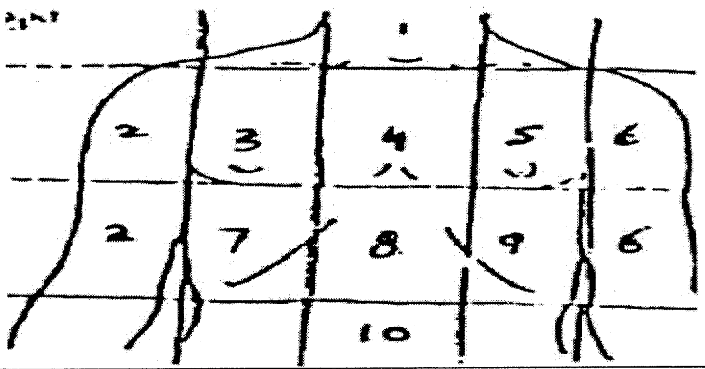

d	کیا اوپر دی گئی علامات آپکو کسی اور حصے میں محسوس ہوئی ہیں؟....................	1-ہاں<br>2-نہیں	
	اگر ہاں تو وضاحت کریں		علامت----------<br>جگہ-----------

سوال نمبر e سے f تک صرف اس صورت میں پوچھیں اگر سوال نمبر b اور c میں کسی کا جواب 1 ہے ورنہ سوال نمبر m پر جائیں۔

e	کیا اوپر دی گئی علامات یا سانس کا پھولنا یا دل کی دھڑکن کا محسوس ہونا آپکو چڑھائی/بلندی پر چلنے یا سیڑھیاں چڑھتے یا تیز چلنے سے محسوس ہوتی ہیں؟............	1-ہاں<br>2-نہیں<br>3-چڑھائی/بلندی پر نہیں چلتے/تیز نہیں چلتے	
f	کیا یہ علامات آپکو ہموار زمین پر معمول کی رفتار سے چلنے پر محسوس ہوتی ہیں؟................	1-ہاں<br>2-نہیں	
g	کیا یہ علامات آپکو آرام کے دوران یا کھانے کے بعد محسوس ہوتی ہیں؟................	1-ہاں<br>2-نہیں	
h	اگر آپکو مشقت کے دوران یہ علامات محسوس ہوتی ہیں تو آپ کیا کرتے ہیں؟...............	1-رک جاتے ہیں<br>2-رفتار کم کر دیتے ہیں<br>3-اسی رفتار سے چلتے رہتے ہیں	

i	رفتار کم کرنے سے یا رک جانے سے کیا یہ علامت چلی جاتی ہیں؟	1۔ہاں<br>2۔ نہیں	☐<br>(اگر جواب 2، ہے تو سوال نمبر k پر جائیں)
j	اگر ہاں تو عام طور پر یہ علامت کتنی جلدی چلی جاتی ہے؟	1۔ تین منٹ سے کم<br>2۔ تین سے بیس منٹ<br>3۔ بیس منٹ سے زیادہ	☐
k	کیا آپ اس علامت سے آرام حاصل کرنے کیلئے عموماً زبان کے نیچے گولی رکھتے ہیں؟..	1۔ہاں<br>2۔ نہیں	☐<br>(اگر جواب 2، ہے تو سوال نمبر m پر جائیں۔)
l	اگر ہاں ہے تو کتنی جلدی یہ علامت دور ہو جاتی ہے؟	1۔دو منٹ سے کم<br>2۔دو سے پانچ منٹ<br>3۔ چھ سے دس منٹ<br>4۔دس منٹ سے زیادہ	☐
m	کیا آپکو کبھی سینے کے سامنے والے حصے میں شدید درد یا بے چینی محسوس ہوئی ہے جو 30منٹ یا اس سے زیادہ رہی ہو؟	1۔ہاں<br>2۔ نہیں	☐<br>(اگر جواب 2، ہے تو اگلے سیکشن پر جائیں)
n	اگر ہاں تو کیا اس درد یا بے چینی کے ساتھ مندرجہ ذیل علامات بھی تھیں؟........		
		1۔ہاں 2۔نہیں<br>جسم کا ٹھنڈا پڑ جانا<br>سانس میں مشکل<br>پسینے آنے	☐<br>☐<br>☐
o	جب آپکو سینے میں شدید درد ہوا اس وقت آپ کی کیا عمر تھی؟........................		سال ☐☐
p	اسطرح کے دورے آپکو کتنی مرتبہ ہوئے ہیں؟..		تعداد ☐☐
q	کیا آپنے کبھی ECG/دل کی پٹی نکلوائی ہے؟............................	1۔ہاں<br>2۔ نہیں	☐
r	کیا آپنے درد کے لئے کسی معالج سے رابطہ کیا ہے؟............................	1۔ہاں<br>2۔ نہیں	☐

II– PERIPHERAL VASCULAR DISEASE

رگوں کی بیماری (جوڑوں کا درد شامل نہیں)

a	کیا آپکو چلنے کے دوران کسی ٹانگ میں درد محسوس ہوتا ہے؟............................	1۔ہاں<br>2۔ نہیں	☐<br>(اگر جواب 2، ہے تو سیکشن III پر جائیں)
b	اگر ہاں تو ٹانگ کے کس حصہ میں محسوس ہوتا ہے؟............................	1۔ ٹانگ کے پچھلے حصے میں<br>2۔ ٹانگ کے پچھے والا حصہ متاثر نہیں ہوتا	☐<br>(اگر جواب 2، ہے تو سیکشن III پر جائیں)

☐ (اگر جواب ،2 ہے تو سیکشن III پر جائیں)	1-ہاں<br>2-نہیں<br>3-لاگو نہیں ہوتا	کیا یہ درد آپکو سیڑھیاں چڑھنے یا تیز چلنے سے ہوتا ہے؟..	c
☐	1-ہاں<br>2-نہیں	کیا ہموار زمین پر معمول کی رفتار سے چلنے سے یہ درد ہوتا ہے؟..............................	d
☐ (اگر جواب ،1 ہے تو سیکشن III پر جائیں)	1-ہاں<br>2-نہیں	کیا یہ درد چلنے کے دوران ہی غائب ہو جاتا ہے؟..............................	e
☐ (اگر جواب ،2 ہے تو سیکشن III پر جائیں)	1-رک جاتے ہیں یا آہستہ ہو جاتے ہیں<br>2-چلنا جاری رکھتے ہیں	اگر یہ درد چلنے کے دوران ہو تو آپ کیا کرتے ہیں؟..............................	f
☐ (اگر جواب ،2 ہے تو سیکشن III پر جائیں)	1-درد ٹھیک ہو جاتا ہے<br>2-ٹھیک نہیں ہوتا	اگر درد ہو اور آپ ایک جگہ کھڑے رہیں تو کیا ہوتا ہے؟..............................	g
☐	1-دس منٹ یا کم<br>2-دس منٹ سے زیادہ	اگر درد ٹھیک ہو جاتا ہے تو کتنی جلدی؟..	h

III- HEART FAILURE

دل کی خرابی (ہارٹ فیل)

☐ (اگر جواب ،1 ہے تو سوال e پر جائیں)	1-ہاں<br>2-نہیں	کیا آپ جسمانی معذوری کے باعث چل نہیں پاتے؟..............................	a
☐	1-ہاں<br>2-نہیں	کیا آپکی سانس اس وقت پھولتی ہے جب اپ ہموار سطح پر معمول کی رفتار سے اپنے ہم عمر لوگوں کے ہمراہ چلتے ہیں	b
☐ (اگر جواب ،2 ہے تو سوال e پر جائیں)	1-ہاں<br>2-نہیں	کیا آپکی سانس آپکے ہم عمر لوگوں کی نسبت زیادہ پھولتی ہے جب چڑھائی پر چل رہے ہوں یا سیڑھیاں چڑھ رہے ہوں؟..............................	c
☐	1-ہاں<br>2-نہیں	کیا آپکو کبھی سانس پھولنے کی وجہ سے رکنا پڑا ہے؟..............................	d
☐ (اگر جواب ،2 ہے تو سوال g پر جائیں)	1-ہاں<br>2-نہیں	پچھلے کچھ سالوں میں کیا آپکی کبھی رات میں سانس پھولنے کی وجہ سے آنکھ کھلی ہے؟....	e
☐	1-ایک سال سے کم<br>2-ایک سال سے زیادہ	یہ مسئلہ آپکو کتنے عرصہ سے ہے؟..............................	f
☐	1-ہاں<br>2-نہیں	کیا آپکو ٹخنوں میں سوجن ہوتی ہیں؟..............................	g
☐	1-ہاں<br>2-نہیں	کیا آپکو کبھی کسی معالج نے بتایا ہے کہ آپکو پھیپھڑوں کی بیماری لاحق ہے مثلاً دمہ، COPD، وغیرہ..............................	h

i	کیا آپکے دل میں کوئی آلہ لگا ہوا ہے؟............	1-ہاں<br>2-نہیں	☐
j	اگر ہاں تو کونسا آلہ لگا ہوا ہے؟............	1-سٹینڈرڈ پیس میکر<br>2-آئی، سی، ڈی<br>3-سی آر ٹی، ڈی	☐

PART-D

COMPLICATIONS

پیچیدگیاں

نیچے دیئے گئے سیکشن صرف اسی صورت میں مکمل کریں اگر ذیابیطس / شوگر سے متعلق Part-B سیکشن II پر کیا ہو۔

I- FOOT ULCERS AND AMPUTATION

پاوں کا زخم اور ضائع ہونا

a	کیا آپکو کبھی پاوں میں ایسا زخم یا چھالہ ہوا ہے جسکو ٹھیک ہونے میں چار ہفتے سے زیادہ وقت لگا ہو؟............	1-ہاں<br>2-نہیں	☐
b	کیا آپ ننگے پاوں چلتے پھرتے ہیں؟............	1-ہاں<br>2-نہیں	☐
c	کیا آپکے جسم کا کوئی حصہ کاٹا گیا ہے؟............	1-ہاں<br>2-نہیں	☐<br>(اگر جواب 2 ہے تو part II پر جائیں)
d	اگر ہاں تو کب............		☐☐ سال پہلے<br>☐☐ مہینے پہلے
e	کونسا حصہ کاٹا گیا؟............	1-انگوٹھا (پاوں کا)<br>2-ٹخنوں سے نیچے<br>3-گھٹنے سے نیچے<br>4-گھٹنے کے اوپر	☐<br>موجودہ حالت لکھیں
f	کاٹنے کی کیا وجہ تھی؟............	1-چوٹ لگنا<br>2-شوگر / ذیابیطس<br>3-انفیکشن<br>4-دیگر	☐ ☐<br>دیگر کی وضاحت کریں<br>............
g	کیا آپکے پاس طبی ریکارڈ یا نسخے موجود ہیں؟............	1-ہاں<br>2-نہیں<br>3-پتہ نہیں	☐
h	اگر جواب ہاں ہے تو جواب دہندہ سے طبی ریکارڈ یا نسخے دیکھانے کیلئے کہیں اور تشخیص نوٹ کریں	تشخیص:-	

II- EYES
آنکھیں

a	کیاآپکو دیکھنے میں کوئی مشکل یا دشواری ہوتی ہے (علاوہ آپکے عام چشموں کے)	1-ہاں<br>2-نہیں	☐<br>(اگر جواب 2، ہے تو سیکشن چھوڑ دیں)
b	اگر ہاں تو کیا آپکو بتایا گیا ہے کہ آپکی نظر کی کمزوری شوگر / ذیابیطس کی پیچیدگیوں کی وجہ سے ہے؟	1-ہاں<br>2-نہیں	☐<br>(اگر جواب 2، ہے تو سیکشن چھوڑ دیں)
c	اگر ہاں تو کیا تشخیص تھی؟....................................		
d	کیا آپ کو آپکی آنکھوں کا کبھی شعاوں(laser) سے علاج کروانا پڑا؟..................<br>(موتیا کے آپریشن کے علاوہ)	1-ہاں<br>2-نہیں	☐
e	کیا آپکے پاس طبی ریکارڈ یا نسخے موجود ہیں؟....................................	1-ہاں<br>2-نہیں<br>3-پتہ نہیں	☐
f	اگر جواب ہاں ہے تو جواب دہندہ سے طبی ریکارڈ یا نسخے دیکھانے کیلئے کہیں اور تشخیص نوٹ کریں	تشخیص:-	

PART-E
RESPIRATORY DISEASE
سانس کی بیماریاں

1	پچھلے ایک سال میں آپکو ایسی کھانسی یا بلغم بننے کی شکایت رہی ہے جو تین ماہ سے زیادہ رہی ہو؟<br>* کھانسی کا مطلب ہے بغیر نزلے، زکام کے کھانسی رہنا ہفتے کی زیادہ تر دنوں اور راتوں میں	1-ہاں<br>2-نہیں	☐<br>(اگر جواب 2، ہے تو سوال 2 پر جائیں)
a	اگر ہاں تو....................................		
i	پچھلے بارہ مہنیوں میں اسطرح کی کھانسی کتنی دفعہ ہوئی؟....................		☐☐
ii	کیا پچھلے بارہ مہنیوں میں آپکو کوئی انفیکشن ہوا جس کیلئے آپکو طبی توجہ کی ضرورت پڑی ہو؟....................................	1-ہاں<br>2-نہیں	☐
iii	پچھلے بارہ مہنیوں میں آپ نے کتنی بار معالج سے رجوع کیا؟....................		☐☐
2	کیا پچھلے بارہ مہنیوں میں آپ نے کسی طبی ماہر یا معالج سے سینے کے انفیکشن (علاوہ ٹی-بی) کیلئے رجوع کیا ہے؟....................................	1-ہاں<br>2-نہیں	☐<br>(اگر جواب 2، ہے تو سوال 3 پر جائیں)
a	اگر ہاں تو....................................		
i	پچھلے بارہ مہنیوں میں اسطرح کتنی دفعہ ہوا؟....................................		☐☐
ii	ان میں سے کتنے، کسی طبی ماہر / ڈاکٹر نے تشخیص کیئے؟....................		تعداد ☐☐
iii	یہ انفیکشن آپکو کتنے عرصے سے ہو رہے ہیں؟....................................		سال ☐☐<br>مہینے ☐☐

Iv	کیاآپ نے ان انفیکشن کیلئے کوئی انٹی بائیٹک ادویات استعمال کی؟..........................	1-ہاں<br>2-نہیں<br>3-پتہ نہیں	☐
3	کیا پچھلے بارہ مہنیوں میں کبھی سینے کے انفیکشن / نمونیہ کیلئے ہسپتال میں داخل ہوئے ہیں؟..............................	1-ہاں<br>2-نہیں	☐<br>(اگر جواب ,2,ہے تو سوال 4پر جائیں)
	اگرہاں توکتنے عرصہ کیلئے..............................		دن ☐☐<br>ہفتے ☐☐<br>مہینے ☐☐
4	کیاآپکو آجکل دمے(Asthma) کی شکایت ہے؟..............................	1-ہاں<br>2-نہیں	☐<br>(اگر جواب ,2,ہے تو سوال 5پر جائیں)
a	اگرہاں تو..............................		
I	پچھلے بارہ مہنیوں میں دمے کے کتنے دورے پڑے ہیں؟..............................		☐☐
II	کیا پچھلے بارہ مہنیوں میں آپکو کوئی انفیکشن رہا جس کیلئے طبی ماہر / ڈاکٹر سے رجوع کرنا پڑا؟..............................	1-ہاں<br>2-نہیں	☐
III	پچھلے بارہ مہنیوں میں آپکو کوئی کتنی دفعہ طبی ماہر / ڈاکٹر سے رجوع کرنا پڑا؟..........		☐☐
5	کیا پچھلے 5سالوں میں آپکو کبھی ٹی۔بی کی تشخیص کی گئی؟..............................	1-ہاں<br>2-نہیں<br>3-یاد نہیں	☐

PART-F

FAMILY HISTORY

اہل وعیال (خاندان) سے متعلق سوالات

1	کیااپکے خاندان میں کسی کوان میں سے کوئی بیماری 60سال کی عمر سے پہلے ہوئی ہے؟		
		**1-ہاں 2-نہیں 3-پتہ نہیں**<br>بلڈ پریشر<br>*دل کی بیماری<br>شوگر/ذیابیطس<br>فالج<br>*انجائنا،دل کا دورہ، اور دل کی خرابی (ہارٹ فیل ہونا)	☐<br>☐<br>☐<br>☐

2 نیچے دیئے گئے ٹیبل کو پر کریں

	فردسے رشتہ	*بیماری (نیچے دئے گئے کوڈز سے رجوع کریں)	تشخیص کے وقت عمر	اگروفات پاچکے ہیں تو کس عمر میں فوت ہوئے
	والد			
	والدہ			
	بیٹا			

				بیٹی
				دادا
				دادی
				نانا
				نانی
				بھائی
				بہن
				چچا
				پھوپھی
				ماموں
				خالہ

ان کے علاوہ مزید رشتہ داروں سے رشتہ اور معلومات نیچے درج کریں۔
*1۔شوگر/ ذیابیطس 2۔دل کی بیماری 3۔بلڈ پریشر 4۔فالج

SECTION-6

TREATMENT HISTORY AND EXPENDITURES

علاج معالجے کی تفصیلات اور اخراجات

PART-A

OUTPATIENT

بیرونی مریض

		کیاآپ ان بیماریوں میں سے کسی کیلئے علاج کروارہے ہیں؟..	1
☐<br>☐<br>☐<br><br>☐<br><br>☐<br>☐	1.ہاں 2.نہیں<br>دل کی بیماری<br>فالج<br>شوگر/ذیابیطس<br><br>شوگر/ذیابیطس کی پیچیدگیاں(انفیکشن آنکھوں اور گردوں کامسئلہ وغیرہ)<br>بلڈ پریشر<br>گردوں کی دائمی (chronic) بیماری		

	اگر اوپر میں سے کسی کا جواب ہاں ہے تو اگلے سیکشن پر جائیں ورنہ Part-B پر جائیں		
	اب میں آپ سے پچھلے بارہ مہینوں کے علاج اور اس کے اخراجات کے بارے میں پوچھوں گا/گی۔		
2	پچھلے بارہ مہینوں میں آپ کتنی مرتبہ کسی مرکز صحت، اہسپتال یا کسی ڈاکٹر کے کلینک گئے ہیں؟		☐☐
3	آپ کونسے مرکز صحت، اہسپتال یا کسی ڈاکٹر کے پاس گئے؟..................................	1-سرکاری<br>2-نجی (پرائیویٹ)<br>3-خیراتی<br>4-دیگر	☐<br>دیگر کی وضاحت کریں<br>....................................
4	اب آپ اس خرچے کی تفصیل بتائیں جا آپ کا اوپر بتائے گئے ہسپتال، مرکز صحت یا ڈاکٹر کے کلینک جانے پر ہوا۔ سوال نمبر 1- میں درج بیماریوں کے خرچے کی تفصیل الگ الگ نوٹ کریں۔		
4i	بیماری..		
	خرچے کی نوعیت	کتنی مرتبہ	خرچہ (روپوں میں)
	ڈاکٹر کی فیس		
	نرس یا کسی دیکھ بھال کرنے والے فرد<br>کو کتنے مہینے تک گھر میں رکھا		
	ٹیسٹ		
	جسمانی بحالی (مثلاً فزیو تھراپی)		
	دیگر (وضاحت کریں)		
	ادویات (پچھلے بارہ مہینوں میں اوپر بتائی گئی بیماری کی ادویات پر خرچ ہونے والی اوسط رقم)		
	پچھلے بارہ مہینوں کا کل خرچہ		
4ii	بیماری..		
	خرچے کی نوعیت	کتنی مرتبہ	خرچہ (روپوں میں)
	ڈاکٹر کی فیس		
	نرس یا کسی دیکھ بھال کرنے والے فرد<br>کو کتنے مہینے تک گھر میں رکھا		
	ٹیسٹ		
	جسمانی بحالی (مثلاً فزیو تھراپی)		
	دیگر (وضاحت کریں)		
	ادویات (پچھلے بارہ مہینوں میں اوپر بتائی گئی بیماری کی ادویات پر خرچ ہونے والی اوسط رقم)		
	پچھلے بارہ مہینوں کا کل خرچہ		
4iii	بیماری..		
	خرچے کی نوعیت	کتنی مرتبہ	خرچہ (روپوں میں)
	ڈاکٹر کی فیس		
	نرس یا کسی دیکھ بھال کرنے والے فرد<br>کو کتنے مہینے تک گھر میں رکھا		

	ٹیسٹ		
	جسمانی بحالی (مثلاً فزیو تھراپی)		
	دیگر (وضاحت کریں)		
	ادویات (پچھلے بارہ مہینوں میں اوپر بتائی گئی بیماری کی ادویات پر خرچ ہونے والی اوسط رقم)		
	پچھلے بارہ مہینوں کا کل خرچہ		
4iv	بیماری..		
	خرچے کی نوعیت	کتنی مرتبہ	خرچہ (روپوں میں)
	ڈاکٹر کی فیس		
	نرس یا کسی دیکھ بھال کرنے والے فرد کو کتنے مہینے تک گھر میں رکھا		
	ٹیسٹ		
	جسمانی بحالی (مثلاً فزیو تھراپی)		
	دیگر (وضاحت کریں)		
	ادویات (پچھلے بارہ مہینوں میں اوپر بتائی گئی بیماری کی ادویات پر خرچ ہونے والی اوسط رقم)		
	پچھلے بارہ مہینوں کا کل خرچہ		
5	کیا آپکو اوپر بتائے گئے خرچے میں سے بیمہ کمپنی کی طرف سے کچھ رقم واپس کی گئ؟..................	1-ہاں<br>2-نہیں<br>3-پتہ نہیں<br>4-کوئی بیمہ نہیں	☐
6	اگر ہاں تو اوپر بتائے خرچے میں سے کتنی رقم آپکو واپس کی گئی		رقم..............................
7	آپکو گھر سے ہسپتال، مرکز صحت یا ڈاکٹر کے کلینک تک پہنچنے میں عام طور پر کتنا وقت لگا؟............		منٹ ☐☐<br>گھنٹے ☐☐
8	اوپر بتائے گئے ہسپتال، مرکز صحت یا ڈاکٹر کے کلینک تک جانے کا کرایہ یا گاڑی کا خرچہ*<br>*(اگر سٹڈی میں شرکت کرنے والے کے پاس اپنی گاڑی ہے تو اس سے پوچھیں کہ مندرجہ بالا جگھوں تک جانے کیلئے پیٹرول، ڈیزل یا گیس کا کتنا خرچہ ہوا)		رقم..............................
9	ہسپتال، مرکز صحت یا ڈاکٹر کے کلینک پر اوسط وقت کتنا لگا؟......................................		منٹ ☐☐<br>گھنٹے ☐☐
10	کیا آپکو صحیح علاج کی سہولت مل رہی ہے؟..	1-ہاں<br>2-نہیں	☐

10a	اگر نہیں تو		
	کس وجہ سے آپکو صحیح علاج کی سہولت نہیں مل رہی ؟..	1-دستیاب نہیں<br>2-مجھے وہاں تک لے جانے والا کوئی نہیں<br>3-بہت دور ہے<br>4-بہت مہنگا ہے<br>5-پیسے خرچ نہیں کرنا چاہتے<br>6-علاج کرانے کا طریقہ کار بہت پیچیدہ ہے<br>7-بہت زیادہ انتظار کرنا پڑتا ہے<br>8-اتنے زیادہ بیمار ہیں کہ وہاں تک جا نہیں سکتے<br>9-طبی سہولتوں کی فراہمی پر اعتماد نہیں کرتے<br>10-پتہ نہیں کہ کہاں پر جانا ہے<br>11-دیگر	☐ ☐ ☐ ☐<br>(دیگر کی وضاحت کریں)<br>..
11	آپنے اپنے علاج اور علاج کیلئے آنے جانے کا خرچہ کہاں سے ادا کیا؟..	**1-ہاں 2-نہیں**<br>1-اپنی بچت سے<br>2-گھر والوں نے دیا<br>3-جہاں کام کرتے ہیں انھوں نے دیا<br>4-دوست، رشتہ دار یا جہاں کام کرتے ہیں ان سے قرضہ لیا<br>5-بینک سے قرضہ لیا<br>6-گھر، زمین یا کوئی چیز بیچی<br>7-صحت کا بیمہ<br>8-دیگر	☐ ☐ ☐ ☐ ☐ ☐ ☐ ☐<br>(دیگر کی وضاحت کریں)<br>..

12	آپنے اپنے علاج اور علاج کیلئے آنے جانے کا خرچہ کتنے فیصد آپنے مندرجہ بالا ذرائع سے ادا کیا؟..............	1-اپنی بچت سے<br>2-گھر والوں نے دیا<br>3-جہاں کام کرتے ہیں انھوں نے دیا<br>4-دوست، رشتہ دار یا جہاں کام کرتے ہیں ان سے قرضہ لیا<br>5-بینک سے قرضہ لیا<br>6-گھر، زمین یا کوئی چیز بیچی<br>7-صحت کا بیمہ<br>8-دیگر	کتنے فیصد ☐<br>کتنے فیصد ☐<br>کتنے فیصد ☐<br>کتنے فیصد ☐<br>کتنے فیصد ☐<br>کتنے فیصد ☐<br>کتنے فیصد ☐<br>کتنے فیصد ☐<br>(دیگر کی وضاحت کریں)<br>....................................

PART-B

INPATIENT

ہسپتال میں داخل مریض

1	کیا آپ پچھلے 12 مہینوں میں کسی بیماری کیلئے ہسپتال مہیں داخل ہوئے تھے؟..............................	1-ہاں<br>2-نہیں<br>3-یاد نہیں	☐<br>(اگر جواب 2 ہے تو سوال 4 پر جائیں)
2	اگر ہاں تو کتنی دفعہ		دفعہ ☐☐
3	کیا آپ مندرجہ ذیل میں سے کسی بیماری کیلئے ہسپتال میں داخل ہوئے تھے؟..............................		
		1-ہاں 2-نہیں<br>دل کی بیماری<br>فالج<br>شوگر/ذیابیطس<br>شوگر/ذیابیطس کی پیچیدگیاں (انفیکشن آنکھوں اور گردوں کا مسئلہ وغیرہ)<br>بلڈ پریشر<br>گردوں کی دائمی (chronic) بیماری	☐<br>☐<br>☐<br>☐<br>☐<br>☐

4	کیا پچھلے 12 مہینوں میں آپ کا کوئی آپریشن ہوا ہے؟	1-ہاں<br>2-نہیں<br>3-یاد نہیں	☐<br>(اگر جواب 2 ہے، part,c پر جائیں)
5	اگر ہاں تو کونسا آپریشن ہوا تھا؟	**1.ہاں 2.نہیں**<br>1-بائی پاس<br>2-دل کے والو کی تبدیلی/مرمت<br>3-پیس میکر<br>4-کسی عضا کا کاٹا جانا<br>5-پھوڑا (پیپ والا)<br>6-گردے کی تبدیلی<br>7-دل کی تبدیلی<br>8-آنکھ کا آپریشن<br>9-دیگر	☐<br>☐<br>☐<br>☐<br>☐<br>☐<br>☐<br>☐<br>☐<br>(دیگر کی وضاحت کریں)<br>..
6	کیا آپکے پاس آپکے ہسپتال میں داخلے / آپریشن کا طبی ریکارڈ موجود ہے؟	1-ہاں<br>2-نہیں	☐
	اگر جواب ہاں ہے تو آپ اپنا طبی ریکارڈ دکھائیں۔ ریکارڈ دیکھ کر تشخیص نوٹ کر لیں۔ ہر دفعہ کی بیماری کی وجہ سے ہسپتال میں داخلے اور آپریشن کا الگ الگ اندراج کریں۔		
	ہسپتال میں داخلہ:-		
	آپریشن:-		
	تبصرہ:-		

PART-C

TREATMENT HISTORY AND EXPENDITURES

ہسپتال میں داخلے کی لاگت (خرچہ)

یہ حصہ صرف اس صورت میں پر کریں اگر اسٹڈی میں شرکت کرنے والا کبھی ہسپتال میں داخل ہوا ہو کسی بھی بیماری کی وجہ سے یا Part-B کے سوال نمبر 3 اور 5 میں درج وجوہات کی وجہ سے، بصورت دیگر انٹرویو ختم کردیں اور اسٹڈی میں شرکت کرنے والے کا شکریہ ادا کریں۔

پچھلے بارہ مہینوں کے دوران ہر دفعہ ہسپتال میں داخلہ کیلئے مندرجہ ذیل تفصیلات نوٹ کریں۔ اگر 3 سے زیادہ مرتبہ ہسپتال میں داخل ہوئے ہیں تو تفصیل لکھنے کیلئے دوسرا فارم استعمال کریں۔

نمبر شمار	سوالات	1	2	3
1	آپ کب ہسپتال میں داخل ہوئے؟...........................	مہینہ ☐☐	مہینہ ☐☐	مہینہ ☐☐
2	آپ کتنے دن ہسپتال میں رہے؟...........................	دن ☐☐	دن ☐☐	دن ☐☐
3	کس قسم کا ہسپتال میں تھا؟...........................	1.ہاں 2.نہیں<br>سرکاری ☐<br>نجی/پرائیویٹ ☐<br>خیراتی ☐<br>دیگر ☐	1.ہاں 2.نہیں<br>سرکاری ☐<br>نجی/پرائیویٹ ☐<br>خیراتی ☐<br>دیگر ☐	1.ہاں 2.نہیں<br>سرکاری ☐<br>نجی/پرائیویٹ ☐<br>خیراتی ☐<br>دیگر ☐
4	ہسپتال کا نام (پتہ)			
5	کس قسم کا علاج یا آپریشن ہوا؟<br>(طبی ریکارڈ اور پارٹ A کی معلومات کے ساتھ چیک کریں)	1.ہاں 2.نہیں<br>ادویات ☐	1.ہاں 2.نہیں<br>ادویات ☐	1.ہاں 2.نہیں<br>ادویات ☐
		تھرومبولیسز ☐<br>Thrombolysis	تھرومبولیسز ☐<br>Thrombolysis	تھرومبولیسز ☐<br>Thrombolysis
		انجیوگرام ☐<br>Angiogram	انجیوگرام ☐<br>Angiogram	انجیوگرام ☐<br>Angiogram
		انجیوپلاسٹی ☐<br>Angioplasty	انجیوپلاسٹی ☐<br>Angioplasty	انجیوپلاسٹی ☐<br>Angioplasty
		بائی پاس ☐<br>Bypass	بائی پاس ☐<br>Bypass	بائی پاس ☐<br>Bypass
		بریکی تھراپی ☐<br>Brachytherapy	بریکی تھراپی ☐<br>Brachytherapy	بریکی تھراپی ☐<br>Brachytherapy
		پیس میکر ☐<br>Pacemaker	پیس میکر ☐<br>Pacemaker	پیس میکر ☐<br>Pacemaker
		دل کی تبدیلی ☐	دل کی تبدیلی ☐	دل کی تبدیلی ☐

		عضو کا کاٹا جانا ☐<br>Amputation	عضو کا کاٹا جانا ☐<br>Amputation	عضو کا کاٹا جانا ☐<br>Amputation
		ایکو کارڈیو گرفی ☐<br>Echocardiography	ایکو کارڈیو گرفی ☐<br>Echocardiography	ایکو کارڈیو گرفی ☐<br>Echocardiograph<br>y
		نیوروامیجنگ ☐<br>Neuro-imaging	نیوروامیجنگ ☐<br>Neuro-imaging	نیوروامیجنگ ☐<br>Neuro-imaging
		ڈئی لیسز ☐<br>Dialysis	ڈئی لیسز ☐<br>Dialysis	ڈئی لیسز ☐<br>Dialysis
		گردے کی تبدیلی ☐	گردے کی تبدیلی ☐	گردے کی تبدیلی ☐
		مشاہدہ کے لیے ☐<br>Observation	مشاہدہ کے لیے ☐<br>Observation	مشاہدہ کے لیے ☐<br>Observation
		دیگر وجوہات ☐	دیگر وجوہات ☐	دیگر وجوہات ☐
		دیگر کی وضاحت کریں<br>..................................	دیگر کی وضاحت کریں<br>..................................	دیگر کی وضاحت کریں<br>..................................<br>...
6	علاج پر کل خرچ شدہ رقم<br>(ہسپتال میں داخلے کا خرچہ +داخلے کے دنوں میں خریدی گئ ادویات کا خرچہ)	--------روپے	--------روپے	--------روپے
7	آپ کا کوئی ساتھی یا دیکھ بھال کرنے والا کتنے دِن تک آپ کے ساتھ ہسپتال میں ٹھہرا؟	دِن ☐☐	دِن ☐☐	دِن ☐☐
8	ساتھی /دیکھ بھال کرنے والے کا ہسپتال میں خرچہ<br>(خوراک، رہائش اور آنے جانے کا خرچہ)	--------روپے	--------روپے	--------روپے
9	آپ کے گھر سے ہسپتال تک کا فاصلہ کتنا ہے ؟....................	کلومیٹر ☐☐☐	کلومیٹر ☐☐☐	کلومیٹر ☐☐☐
10	گھر سے ہسپتال تک آنے کا ٹرانسپورٹ کا خرچہ<br>(ایمبولینس کے خرچہ کے علاوہ)	--------روپے	--------روپے	--------روپے
11	آپ کے پاس طبی بیمہ کس قسم کا ہے ؟..................................	1-ہاں 2-نہیں<br>مفت علاج ☐<br>تجارتی بیمہ ☐<br>کوئی نہیں ☐<br>خود پیسے دیتے ہیں ☐<br>دیگر ☐<br>دیگر کی وضاحت کریں<br>..................................	1-ہاں 2-نہیں<br>مفت علاج ☐<br>تجارتی بیمہ ☐<br>کوئی نہیں ☐<br>خود پیسے دیتے ہیں ☐<br>دیگر ☐<br>دیگر کی وضاحت کریں<br>..................................	1-ہاں 2-نہیں<br>مفت علاج ☐<br>تجارتی بیمہ ☐<br>کوئی نہیں ☐<br>خود پیسے دیتے ہیں ☐<br>دیگر ☐<br>دیگر کی وضاحت کریں<br>..................................
12	رقم جو آپ کو صحت کے بیمہ سے واپس ادا کردی گئ ہو؟	--------روپے	--------روپے	--------روپے

13	ہسپتال میں داخلے کا خرچہ آپ کس طرح ادا کرتے ہیں؟............	1-ہاں 2-نہیں<br>اپنی بچت سے ☐<br>گھر والوں نے دیا ☐<br>جہاں کام کرتے ہیں اُنہوں نے دیا ☐<br>کسی دوست، رشتہ دار، یا جہاں کام کرتے ہیں اُن سے قرضہ لیا ☐<br>بینک سے قرضہ لیا ☐<br>گھر زمین یا کوئی اور چیز بیچی ☐<br>صحت کا بیمہ ☐<br>دیگر ☐<br>(دیگر کی وضاحت کریں)<br>....................................	1-ہاں 2-نہیں<br>اپنی بچت سے ☐<br>گھر والوں نے دیا ☐<br>جہاں کام کرتے ہیں اُنہوں نے دیا ☐<br>کسی دوست، رشتہ دار، یا جہاں کام کرتے ہیں اُن سے قرضہ لیا ☐<br>بینک سے قرضہ لیا ☐<br>گھر زمین یا کوئی اور چیز بیچی ☐<br>صحت کا بیمہ ☐<br>دیگر ☐<br>(دیگر کی وضاحت کریں)<br>....................................	1-ہاں 2-نہیں<br>اپنی بچت سے ☐<br>گھر والوں نے دیا ☐<br>جہاں کام کرتے ہیں اُنہوں نے دیا ☐<br>کسی دوست، رشتہ دار، یا جہاں کام کرتے ہیں اُن سے قرضہ لیا ☐<br>بینک سے قرضہ لیا ☐<br>گھر زمین یا کوئی اور چیز بیچی ☐<br>صحت کا بیمہ ☐<br>دیگر ☐<br>(دیگر کی وضاحت کریں)<br>....................................
14	اپنے ہسپتال میں داخلہ کے خرچہ کا کتنے فیصد آپنے مندرجہ بالا ذرائع سے ادا کیا؟	اپنی بچت سے...........%<br>گھر والوں نے دیا.........%<br>جہاں کام کرتے ہیں اُنہوں نے دیا...........%<br>کسی دوست، رشتہ دار، یا جہاں کام کرتے ہیں اُن سے قرضہ لیا.............%<br>بینک سے قرضہ لیا.....%<br>گھر زمین یا کوئی اور چیز بیچی........%<br>صحت کا بیمہ.......%<br>دیگر........%<br>(دیگر کی وضاحت کریں)<br>....................................	اپنی بچت سے........%<br>گھر والوں نے دیا.......%<br>جہاں کام کرتے ہیں اُنہوں نے دیا........%<br>کسی دوست، رشتہ دار، یا جہاں کام کرتے ہیں اُن سے قرضہ لیا........%<br>بینک سے قرضہ لیا.......%<br>گھر زمین یا کوئی اور چیز بیچی.......%<br>صحت کا بیمہ.......%<br>دیگر.......%<br>(دیگر کی وضاحت کریں)<br>....................................	اپنی بچت سے........%<br>گھر والوں نے دیا......%<br>جہاں کام کرتے ہیں اُنہوں نے دیا........%<br>کسی دوست، رشتہ دار، یا جہاں کام کرتے ہیں اُن سے قرضہ لیا........%<br>بینک سے قرضہ لیا.......%<br>گھر زمین یا کوئی اور چیز بیچی.......%<br>صحت کا بیمہ.......%<br>دیگر.......%<br>(دیگر کی وضاحت کریں)<br>....................................
15	انٹرویو ختم ہونے کا وقت	منٹ ☐☐	گھنٹے ☐☐	

FORM - A

CARRS: SURVEILLANCE STUDY
BLOOD PRESSURE AND ANTHROPOMETRY

Participant ID ☐☐☐☐☐

Interviewer I ☐☐☐☐

Date Completed: ☐☐☐☐☐☐

DD / MM / YY

I. BLOOD PRESSURE AND PULSE

Instrument ID ☐☐

Type of Measurement	1st Reading	2nd Reading	Difference between 1st and 2nd	Tolerance	3rd Reading (If necessary)
Systolic BP	☐☐☐	☐☐☐	☐☐☐	10 mm Hg	☐☐☐
Diastolic BP	☐☐☐	☐☐☐	☐☐☐	6 mm Hg	☐☐☐
Pulse rate	☐☐☐	☐☐☐			

II. ANTHROPOMETRIC MEASUREMENTS

**1. Height (cm)**		Instrument ID ☐☐
Standing Height	I__I__I__I . I__I	Comments:

**2. Body circumferences (cm)**		Instrument ID ☐☐			
Mid-arm	Clothing (√)	Waist	Clothing (√)	Hip	Clothing (√)
I__I__I__I . I__I	None ☐<br>Light ☐<br>Heavy ☐	I__I__I__I . I__I	None ☐<br>Light ☐<br>Heavy ☐	I__I__I__I . I__I	None ☐<br>Light ☐<br>Heavy ☐

**3. Skinfold Thickness (mm)**		Instrument ID ☐☐			
**Type of Measurement**	**1st Reading**	**2nd Reading**	**Difference between 1st and 2nd**	**Tolerance**	**3rd Reading (If necessary)**
Triceps	I__I__I . I__I	I__I__I . I__I	I__I . I__I	1 mm	I__I__I . I__I
Supra-scapular	I__I__I . I__I	I__I__I . I__I	I__I . I__I	1 mm	I__I__I . I__I
Supra-patellar	I__I__I . I__I	I__I__I . I__I	I__I . I__I	1 mm	I__I__I . I__I

Attach the print-out of body composition / bio-impedance measurement of the participant along with this form. Note any specific comments in the space below.

____

____

____

____

SECTION-7

LABORATORY TESTS RESULTS

A	**Lab #**				
1	Fasting Glucose		2	Serum Cholesterol	
3	HDL		4	LDL	
5	VLDL		6	Triglycerides	
7	Serum Urea		8	Serum Creatinine	
9	Hemoglobin A1c	gm			
10	Urine Microalbuminuria(Quantitative)				
11	Salivary Cotinine				

Data Collector Comments
